# International Union of Basic and Clinical Pharmacology. CV. Somatostatin Receptors: Structure, Function, Ligands, and New Nomenclature

**DOI:** 10.1124/pr.117.015388

**Published:** 2018-10

**Authors:** Thomas Günther, Giovanni Tulipano, Pascal Dournaud, Corinne Bousquet, Zsolt Csaba, Hans-Jürgen Kreienkamp, Amelie Lupp, Márta Korbonits, Justo P. Castaño, Hans-Jürgen Wester, Michael Culler, Shlomo Melmed, Stefan Schulz

**Affiliations:** Institute of Pharmacology and Toxicology, Jena University Hospital, Friedrich-Schiller-University, Jena, Germany (T.G., A.L., S.S.); Unit of Pharmacology, Department of Molecular and Translational Medicine, University of Brescia, Brescia, Italy (G.T.); PROTECT, INSERM, Université Paris Diderot, Sorbonne Paris Cité, Paris, France (P.D., Z.C.); Cancer Research Center of Toulouse, INSERM UMR 1037-University Toulouse III Paul Sabatier, Toulouse, France (C.B.); Institute of Human Genetics, University Medical Center Hamburg-Eppendorf, Hamburg, Germany (H.-J.K.); Centre for Endocrinology, William Harvey Research Institute, Barts and London School of Medicine, Queen Mary University of London, London, United Kingdom (M.K.); Maimonides Institute for Biomedical Research of Cordoba, Córdoba, Spain (J.P.C.); Department of Cell Biology, Physiology, and Immunology, University of Córdoba, Córdoba, Spain (J.P.C.); Reina Sofia University Hospital, Córdoba, Spain (J.P.C.); CIBER Fisiopatología de la Obesidad y Nutrición, Córdoba, Spain (J.P.C.); Pharmaceutical Radiochemistry, Technische Universität München, Munich, Germany (H.-J.W.); Culler Consulting LLC, Hopkinton, Massachusetts (M.C.); and Pituitary Center, Department of Medicine, Cedars-Sinai Medical Center, Los Angeles, California (S.M.)

## Abstract

Somatostatin, also known as somatotropin-release inhibitory factor, is a cyclopeptide that exerts potent inhibitory actions on hormone secretion and neuronal excitability. Its physiologic functions are mediated by five G protein–coupled receptors (GPCRs) called somatostatin receptor (SST)1–5. These five receptors share common structural features and signaling mechanisms but differ in their cellular and subcellular localization and mode of regulation. SST_2_ and SST_5_ receptors have evolved as primary targets for pharmacological treatment of pituitary adenomas and neuroendocrine tumors. In addition, SST_2_ is a prototypical GPCR for the development of peptide-based radiopharmaceuticals for diagnostic and therapeutic interventions. This review article summarizes findings published in the last 25 years on the physiology, pharmacology, and clinical applications related to SSTs. We also discuss potential future developments and propose a new nomenclature.

## I. Introduction and Historical Perspective

**Fig. 1.**
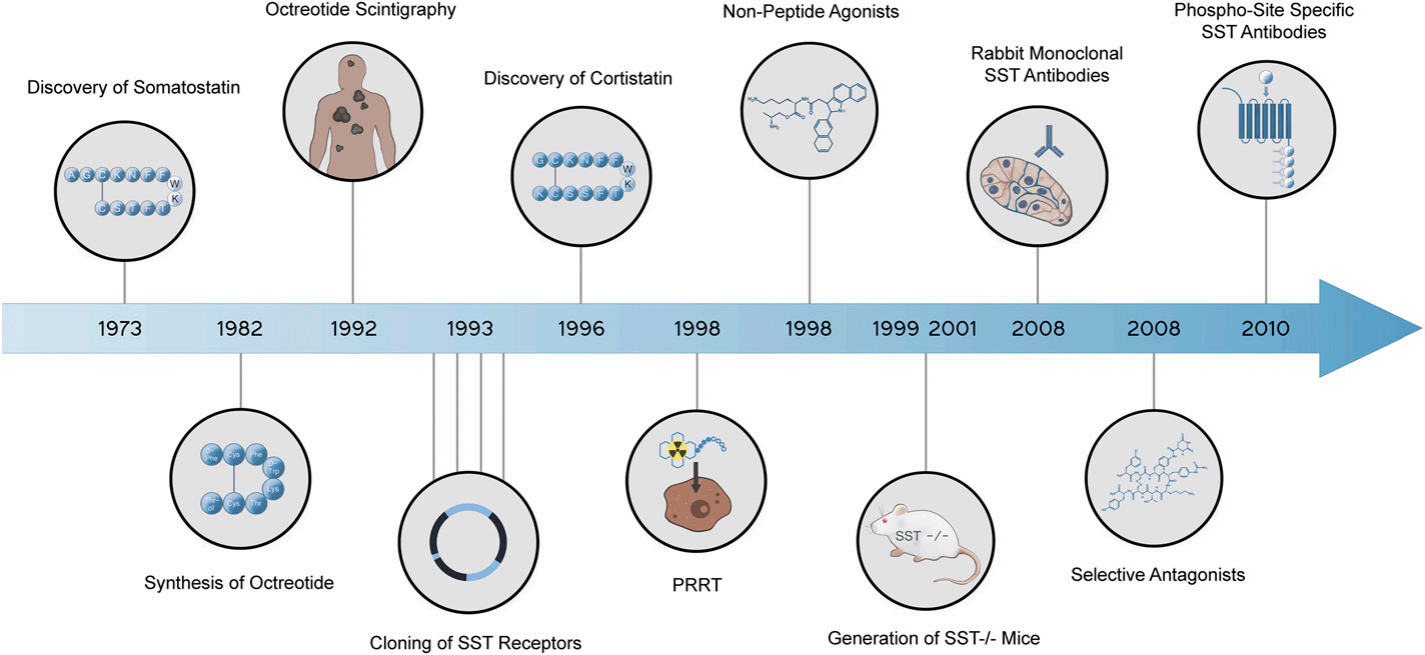
Historical perspective of somatostatin and somatostatin receptor research.

Since their discovery, research on somatostatin and its receptors has remained active with more than 700 papers published annually. Somatostatin—also known as somatotropin release-inhibiting factor (SRIF)—was originally discovered in 1973 as a hypothalamic neuropeptide based on its ability to inhibit growth hormone (GH) release from the anterior pituitary (Fig. 1) (Brazeau et al., 1973). SRIF occurs in two forms, SRIF-14 and SRIF-28, with broad antisecretory activity on many hormones, including GH, insulin, glucagon, gastrin, cholecystokinin (CCK), and ghrelin. In the original report, it was suggested that SRIF could have potential for treatment of acromegaly. However, due to its short circulating half-life (*t*_1/2_) (<3 minutes), the therapeutic potential of natural SRIF-14 is limited. Consequently, highly potent and metabolically stable SRIF analogs were synthesized in 1982 (Bauer et al., 1982). The first SRIF analog approved for clinical use was octreotide (Lamberts et al., 1996). About 10 years later, the first SRIF-based radiopharmaceuticals were synthesized by conjugating a chelator to octreotide, followed by radiolabeling with a *γ*-emitter, which paved the way for in vivo imaging of human tumors (Bakker et al., 1991a,b; Krenning et al., 1993). In the early 1990s, five subtypes of somatostatin receptors (SSTs) termed SST_1_ to SST_5_ were cloned in mice, rats, and humans (Meyerhof et al., 1991, 1992; Bruno et al., 1992; Kluxen et al., 1992; Li et al., 1992; O’Carroll et al., 1992; Yamada et al., 1992a,b, 1993; Yasuda et al., 1992; Rohrer et al., 1993; Panetta et al., 1994; Schwabe et al., 1996; Lublin et al., 1997). Given that only two SRIF tissue binding sites could be identified using ligands available at that time, the subsequent discovery of five different SSTs was surprising and triggered in-depth research into binding properties, localization, and regulation of the ligand. This led to classification of the clinically used SRIF analogs octreotide and lanreotide as SST_2_-prefering ligands, which in turn stimulated the search for novel compounds that bind either more broadly or more selectively to individual SSTs. In 1996, a structurally related neuropeptide termed cortistatin (CST) with a more restricted distribution in the cerebral cortex and hippocampus was identified (de Lecea et al., 1996). In the late 1990s, knockout (KO) mice and selective nonpeptide agonists were developed for all five SSTs, which helped to define their physiologic functions (Zheng et al., 1997; Kreienkamp et al., 1999; Strowski et al., 2003; Tirone et al., 2003; Qiu et al., 2005, 2008; Tallent et al., 2005; Einstein et al., 2010). In 1998, the development of octreotide conjugates radiolabeled with a *β*-emitter provided proof-of-principle for peptide-receptor radiotherapy (PRRT) (Stolz et al., 1998). A few years later, the capacity of SSTs to form homodimers and heterodimers with other G protein**–**coupled receptors (GPCRs) was observed, which stimulated the search for bitopic chimeric compounds (Rocheville et al., 2000a; Pfeiffer et al., 2001, 2002). In the early 2000s, the search for multireceptor ligands led to the discovery of pasireotide, which was the first pituitary-directed drug approved for therapy of Cushing’s disease (Bruns et al., 2002; Colao et al., 2012). In 2008, high-affinity peptide antagonists with utility for SST imaging and treatment were synthesized, suggesting that receptor internalization is not an absolute requirement for tumor imaging (Cescato et al., 2008). Also in 2008, the generation of highly specific rabbit monoclonal antibodies (mAbs) facilitated detection of SSTs in human tissues and enabled correlation of SST_2_ and SST_5_ receptor expression with octreotide and pasireotide responses (Fischer et al., 2008; Lupp et al., 2011). Shortly thereafter, development of phosphosite-specific antibodies provided molecular insights into mechanisms for SST activation by octreotide and pasireotide (Poll et al., 2010; Petrich et al., 2013; Lehmann et al., 2016). In 2009, a novel truncated variant of SST_5_ generated by aberrant splicing was identified [SST_5_ transmembrane domain (SST_5_TMD4)] and shown to be overexpressed in several hormone-related tumors, wherein the variant increases aggressiveness (Durán-Prado et al., 2009, 2012b; Gahete et al., 2010a; Hormaechea-Agulla et al., 2017). In the past few years, orally available and subtype-selective SST agonists and antagonists have been synthesized. Some of these substances may become lead compounds for potential new therapeutic indications directed toward individual SSTs (He et al., 2014; Hirose et al., 2017).

## II. Endogenous Ligands

### A. Somatostatin

#### 1. Somatostatin Gene and Peptide Structure

**Fig. 2.**
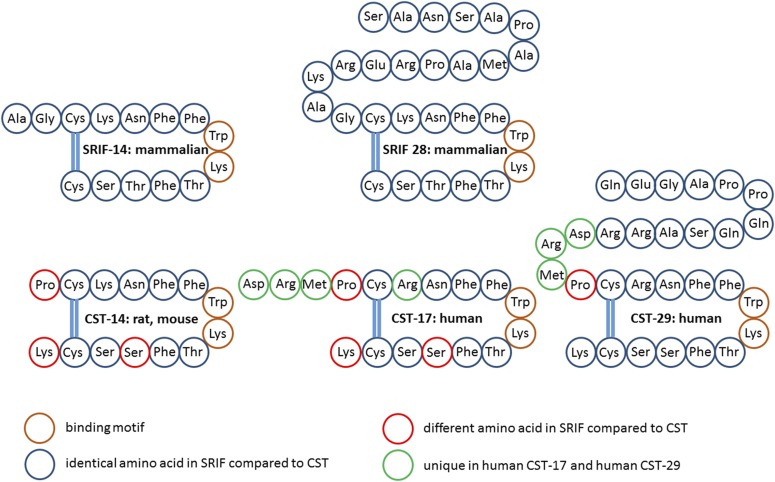
Primary and secondary amino acid structure of mammalian SRIF and CST isoforms. Color code: brown, binding motif; blue, identical in SRIF and CST; red, different in CST compared with SRIF; green, not present in rat/mouse CST-14.

SRIF, a cyclic neuroendocrine peptide, was first isolated and identified as a hypothalamic factor that inhibited GH secretion from anterior pituitary cells (Brazeau et al., 1973). SRIF exists in two main bioactive isoforms: the tetradecapeptide (SRIF-14) isolated from the hypothalamus and the 28-amino-acid isoform (SRIF-28), generated from the same prepro-SRIF precursor through post-translational processing at a distinct cleavage site and which differs from the shorter isoform by an N-terminal extension (Fig. 2) (Esch et al., 1980; Pradayrol et al., 1980; Shen and Rutter, 1984). Both isoforms are expressed at variable amounts in the same tissue areas. It is not clear whether the two peptides are coexpressed by the same cells or by separate cells. The family of somatostatin peptides includes CST, a highly similar peptide reviewed below, and which is structurally and functionally related to the urotensin II peptide family. The two families (somatostatin and urotensin II) as well as those of their respective GPCRs may derive from a single ancestral ligand–receptor pair. The duplication, generating the two families, likely occurred before the emergence of vertebrates. Subsequently, each family expanded during evolution, through whole-genome duplications, followed by local duplications and gene losses (Tostivint et al., 2014). Despite their evolutional divergence, the two families conserve close functional links (Malagon et al., 2008). The vertebrate SRIF family is composed of at least six paralogous genes named *SS1* to *SS6* (Liu et al., 2010). In mammals, SRIF-14 and SRIF-28 both derive from the *SS1* gene, localized on chromosome 3q27.3 in humans. The SRIF-14 primary structure is highly conserved in vertebrates, and cleavage sites generating SRIF-14 and its extended isoform have been fully conserved during evolution (Conlon et al., 1997). An additional product of the mammalian processing of prepro-SRIF is a 13-amino-acid noncyclic amidated peptide, neuronostatin, which immediately follows the signal peptide (Samson et al., 2008; Yosten et al., 2015). Bioinformatic analyses of evolutionary conserved sequences suggest the occurrence of neuronostatin in other vertebrates. A novel peptide showing structural similarity to SRIF-28 and isolated from monkey ileum comprises amino acid sequences matching the N-terminal 13 amino acids of SRIF-28. This peptide is expressed in enteric neurons and may play a possible role in food intake control (Ensinck et al., 2002, 2003).

#### 2. Regulation of Gene Expression and Peptide Release

The structure of rat and human SRIF genes, as well as the transcriptional unit and upstream regulatory elements of the rat gene, has been characterized (Montminy et al., 1984; Shen and Rutter, 1984). Expression of the SRIF precursor gene is regulated by growth factors and cytokines, including GH, insulin-like growth factor 1 (IGF-1), insulin, leptin, and inflammatory cytokines, and by glucocorticoids, testosterone, and estradiol. cAMP is a potent activator of SRIF transcription, and SRIF release from neurons and peripheral secretory cells is triggered by membrane depolarization and increased cytosolic calcium concentrations. Several neurotransmitters, neuropeptides, hormones, and nutrients, some also altering gene transcription, affect SRIF release in the central nervous system (CNS) and in peripheral tissues (Montminy et al., 1996; Müller et al., 1999; Patel, 1999; Eigler and Ben-Shlomo, 2014). Characterization of neurotransmitter, neuropeptide, and hormone modulation of hypothalamic SRIFergic neurons has raised interest in light of the key role played by SRIF as a distal mediator for neuroendocrine and metabolic control of the GH axis activity in health and disease (Müller et al., 1999).

#### 3. Anatomic Framework

Abundant SRIF immunoreactivity is evident in the mediobasal hypothalamus and median eminence, amygdala, preoptic area, hippocampus, striatum, cerebral cortex, olfactory regions, and the brainstem (Johansson et al., 1984). Three main categories of SRIFergic neurons can be distinguished: hypophysiotropic neurons, long-projecting GABAergic neurons, and GABAergic interneurons acting within microcircuits (Viollet et al., 2008; Urban-Ciecko and Barth, 2016).

In the rat CNS, SRIFergic neurons regulating pituitary function are located within the periventricular nucleus and the parvocellular part of the paraventricular nucleus and send axonal projections to the median eminence at the base of the hypothalamus. SRIF-producing neuronal cell bodies are also found in the arcuate (ARC) and ventromedial nuclei. Hypophysiotropic SRIFergic neuronal axons descend toward the pituitary stalk and release SRIF into the portal blood vessel system, thereby reaching anterior pituitary cells. Some axons travel through the neural pituitary stalk into the neurohypophysis. Other fibers project outside the hypothalamus to areas such as the limbic system or may interact, through interneurons, with other hypothalamic nuclei, including the ARC where GH-releasing hormone (GHRH) is expressed, the preoptic nucleus, the ventromedial nucleus, and the suprachiasmatic nucleus, which exhibits circadian pacemaker activity (Müller et al., 1999; Eigler and Ben-Shlomo, 2014). SRIF is ubiquitously expressed in mammalian brain. Extrahypothalamic SRIF immunoreactivity is found in the amygdala, preoptic area, hippocampus, striatum, cerebral cortex, sensory regions, and brainstem. SRIF neurons are classified into two main categories: interneurons acting locally within microcircuits and long-range–projecting neurons whose fibers reach distant areas. SRIF frequently colocalizes with GABA. SRIFergic interneurons likely play a role in regulation of distal dendrite excitability, and long-range–projecting SRIFergic neurons may participate in coordinating activity between distant brain regions. Accordingly, nonpyramidal cells located in the hippocampus and targeting the medial septum and the medial entorhinal cortex form inhibitory synapses on GABAergic interneurons, and may cooperate in generation and synchronization of rhythmic oscillatory activity in these areas (Viollet et al., 2008; Melzer et al., 2012). Recently, brain-wide SRIF neuron maps have established the precise cell number, density, and somatic morphology with anatomic references for SRIF-related specific functions (Kim et al., 2017; Zhang et al., 2017).

Peripheral SRIF is produced by secretory cells in gastrointestinal mucosa and by *δ*-cells in pancreatic islets, as well as by a subpopulation of C cells within the thyroid gland. In addition to SRIF-producing neuroendocrine cells, inflammatory and immune response cells and tumor cells may also express SRIF (Patel, 1999). The peptide has been immunolocalized in human epidermis, with low expression levels in keratinocytes and higher expression in subsets of Merkel and dendritic cells (Vockel et al., 2010, 2011). SRIF is a neurotransmitter and neuromodulator, an endocrine hormone and a paracrine factor acting in the same tissue where it is expressed. Circulating levels of SRIF are very low because the peptide is rapidly degraded by ubiquitous peptidases (Rai et al., 2015). In the retina, SRIF was detected by immunohistochemical studies in amacrine and ganglion cells, as well as in interplexiform cells, whereas electrophysiological studies support the view that it may function as a neurotransmitter, neuromodulator, or trophic factor (Cervia et al., 2008).

#### 4. Functions

Within the hypothalamus–pituitary system, SRIF is the main regulatory element exerting inhibitory control on both basal and stimulated GH secretion and reduces prolactin and thyroid-stimulating hormone (TSH) secretion in normal subjects (Müller et al., 1999). It can also suppress release of adrenocorticotropic hormone (ACTH) from tumor cells (Hofland et al., 2010). SRIF brain actions are mediated by presynaptic or postsynaptic mechanisms. SRIF modulates neuronal excitability, and in the hippocampus, cortex, and hypothalamus it also induces presynaptic inhibition of excitatory neurotransmission (Peineau et al., 2003). In other brain areas, SRIF also decreases GABA release. Postsynaptic mechanisms of action include membrane hyperpolarization via activation of potassium ion currents (K^+^ currents), in particular voltage-gated K^+^ currents, noninactivating potassium currents (M currents), and voltage-insensitive leak currents (Moore et al., 1988; Schweitzer et al., 1998; Jiang et al., 2003; Qiu et al., 2008). SRIF is coreleased with GABA from hippocampal neurons and from axonal terminals in other brain areas (Olias et al., 2004). SRIF inhibits dopamine release from the midbrain as well as hypothalamic release of noradrenaline, thyrotropin-releasing hormone, and corticotropin-releasing hormone (CRH) (Patel, 1999). Activation of brain SRIF signaling may alleviate endocrine, autonomic, and behavioral responses to stress mediated by central CRH and CRH receptors (Stengel and Taché, 2017). SRIF has a role in cognitive functions, learning and memory processes, control of locomotor activity, control of food intake, nociception, and autonomic functions. SRIF is highly expressed in brain regions associated with seizures and has been suggested as an endogenous antiepileptic (Olias et al., 2004; Tallent and Qiu, 2008; Stengel et al., 2015).

Peripheral SRIF actions include inhibition of hormone secretion, exocrine secretion, and cell proliferation. In the gastrointestinal (GI) tract (GIT), SRIF exerts a generalized inhibitory effect on release of gut hormones [including gastrin, CCK, gastric inhibitory polypeptide, vasoactive intestinal peptide, enteroglucagon, motilin], gastric acid, digestive enzymes, bile, and colonic fluid. SRIF also negatively affects gallbladder contraction, small intestinal segmentation, and gastric emptying. In pancreatic islets, release of SRIF from *δ*-cells inhibits secretion of insulin, glucagon, and other peptides from neighboring cells. SRIF reduces TSH-induced release of triiodothyronine (T3) and thyroxine as well as calcitonin release. In the adrenals, SRIF inhibits angiotensin II–stimulated aldosterone secretion and acetylcholine-stimulated medullary catecholamine secretion. SRIF reduces release of kidney-derived renin caused by hypovolemia and vasopressin-mediated water absorption. In addition to nervous system functions and regulation of endocrine and GI functions, SRIF also may affect key cellular processes in diverse tissues by regulating the release of both growth factors and cytokines as well as cellular responses to these stimuli. SRIF can contribute to control of smooth muscle cell contractility, lymphocyte and inflammatory cell proliferation and activity, tumor cell growth, and normal tissue plasticity (Patel, 1999; Rai et al., 2015). In human skin, SRIF has been suggested as a negative regulator of epidermal wound healing (Vockel et al., 2011). Finally, at variance with its nearly universal inhibitory actions, low (pM) concentrations of SRIF stimulate in vitro GH release on cultured pituitary cells derived from pigs (Luque et al., 2006) and nonhuman primates (Cordoba-Chacon et al., 2012b) and from human somatotroph adenomas (Matrone et al., 2004).

### B. Cortistatin

CST, a cyclic neuropeptide, highly homologous to SRIF, was identified as a region-specific brain mRNA encoding a protein of 112-amino-acid residues, which was called preproCST (de Lecea et al., 1996). CST in mammals derives from the *CORT* gene (Liu et al., 2010). The gene encoding for human and mouse CST is located on 1p36.3–1p36.2 and on chromosome 4, respectively. Similarly to preproSRIF, cleavage of preproCST gives rise to multiple mature products, CST-14 and CST-29 in rats and CST-17 and CST-29 in humans (Fig. 2). CST-14 and SRIF-14 differ in three amino acid; CST aligns with the second-amino-acid residue of SRIF on the N-terminus and terminates one-amino-acid residue beyond the C-terminal of SRIF (de Lecea et al., 1997b). Human CST-17 contains an arginine for lysine substitution and is extended by three amino acids at the amino-terminal end, resulting in CST-17 sharing 10 of the 14 SRIF-14 residues. Similar to their prepropeptides, mature CST and SRIF are also highly homologous, including the two cysteine residues that render the peptides cyclic as well as a FWKT motif critical for SST binding (de Lecea et al., 1997b). Consequently, CST peptides bind to all SST subtypes with similar affinity than SRIF, and yet there is no evidence for a selective cortistatin receptor (Siehler et al., 2008). Notably, the FWKT motif is also present in urotensin II and urotensin-related peptide, which are indeed agonists of SSTs (Vaudry et al., 2015).

PreproCST mRNA is predominantly expressed in the cerebral cortex and hippocampus. In the cortex, mainly layers II–III and VI contain CST-positive cells. Interestingly, CST-positive cell bodies are not uniformly distributed in all cortical areas, with highest numbers evident in the visual and temporal cortex and lowest in the somatosensory cortex (de Lecea et al., 1997a). CST-containing neurons are also detected in the piriform cortex and entorhinal area. In the hippocampus, CST expression is found in a small subset of nonpyramidal neurons of the subiculum and in the stratum oriens of hippocampus subfields Cornu Ammonis (CA)1–3. In the hilar region of the dentate gyrus, however, CST-positive neurons are only transiently present during development (de Lecea et al., 1997a). In parallel, there is a temporary increase of cortical CST expression during development, which correlates with maturation of cortical interneurons. Indeed, cortical CST-expressing neurons, similarly to SRIF-positive neurons, are also GABAergic. However, CST- and SRIF-containing neurons are expressed in distinct, only partially overlapping populations (de Lecea et al., 1997a). CST is also expressed in other brain areas, such as in the olfactory bulb, in the striatum, in the periventricular nucleus of the hypothalamus, and in GABAergic interneurons of the deep layers of the spinal cord dorsal horn (de Lecea, 2008; Morell et al., 2013). No CST expression was detected in the thalamus, brainstem, or cerebellum (de Lecea, 2008). The projections of CST-positive neurons were not analyzed in detail, but due to their high homology it is possible that anti-SRIF antibodies may also label CST-containing axons.

CST is also expressed in the periphery, in general at lower level than SRIF but with a broader distribution: preproCST mRNA was detected in peripheral nociceptive neurons, endocrine organs (e.g., pituitary gland, adrenal gland, thyroid gland, parathyroid gland, endocrine pancreas), digestive system (e.g., stomach, jejunum, ileum, colon, rectum, liver), kidney, lung, and gonads, and also in smooth muscle cells, endothelial cells, and immune cells (e.g., lymphocytes, monocytes, macrophages, dendritic cells) (Broglio et al., 2007; Gahete et al., 2008; Gonzalez-Rey et al., 2015). Discrepancies between mRNA expression and protein synthesis were observed in several tissues (including adrenal, thyroid, lung, and gonads) (Broglio et al., 2007). Notably, predominant CST is present in parathyroid chief cells and immune cells (Dalm et al., 2003a; Allia et al., 2005).

In keeping with their similar affinities to SSTs, CST and SRIF share several biologic properties, including inhibition of neuronal activity and consequent antiepileptic activity (Braun et al., 1998; Aourz et al., 2014), inhibition of cell proliferation, and regulation of hormones, and particularly inhibition of GH secretion (Spier and de Lecea, 2000). Double KO mice devoid of both SRIF and CST show markedly increased GH levels, although they do not display overt giant phenotypes (Pedraza-Arevalo et al., 2015). Nevertheless, functional differences of CST and SRIF can only partly be attributed to their distinct tissue distributions (de Lecea and Castaño, 2006). At the cellular level, CST, similarly to SRIF, increases the M current in hippocampal neurons but also augments the hyperpolarization-activated currents (Schweitzer et al., 2003), thereby modulating synaptic integration and regulation of oscillatory activity. At the behavioral level, CST induces hypomotility, whereas SRIF causes hypermotility (Criado et al., 1999); CST enhances slow-wave sleep, whereas SRIF increases rapid eye movement sleep (de Lecea et al., 1996; Bourgin et al., 2007). CST and SRIF also regulate differently endocrine functions (Ibáñez-Costa et al., 2017b) as well as learning and memory processes (Borbély et al., 2013). Consistent with its widespread distribution in the immune system (Dalm et al., 2003b), CST is a potent anti-inflammatory factor, decreasing the production of several inflammatory cytokines [tumor necrosis factor-*α*, interleukin (IL)-1*β*, IL-6, IL-12, interferon-*γ*], chemokines, and acute-phase proteins (Gonzalez-Rey et al., 2015). CST also inhibits T helper 1- and 17-driven inflammatory responses in models of inflammation [e.g., sepsis (Gonzalez-Rey et al., 2006a), atherosclerosis (Delgado-Maroto et al., 2017)], and autoimmune diseases [e.g., inflammatory bowel disease (Gonzalez-Rey et al., 2006b), rheumatoid arthritis (Gonzalez-Rey et al., 2007), and multiple sclerosis (Souza-Moreira et al., 2013)]. In parallel with its potent anti-inflammatory effect, CST is also an endogenous analgesic factor acting at both the peripheral and spinal level (Morell et al., 2013).

CST may activate GPCRs other than SSTs (Ibáñez-Costa et al., 2017b), including the ghrelin receptor 1a (GHS-R1a) (Callaghan and Furness, 2014) and human-specific MAS-related GPR family member X2 (Solinski et al., 2014). Functions of CST not shared by SRIF in the immune (Gonzalez-Rey et al., 2015) and endocrine (Cordoba-Chacon et al., 2011) systems are likely mediated by GHS-R1a, whereas MAS-related GPR family member X2 might play an important role in the analgesic effects of CST in humans. In addition, some CST-specific functions might be mediated by a yet unidentified CST-selective receptor (Gonzalez-Rey et al., 2015), and by truncated SST_5_ variants that selectively respond to CST (Gahete et al., 2008; Durán-Prado et al., 2009; Cordoba-Chacon et al., 2010, 2011; Ibáñez-Costa et al., 2017b).

## III. Somatostatin Receptors

### A. Nomenclature

There is yet considerable misconception and lack of clarity regarding classification and nomenclature of SSTs. SRIF binding sites were initially defined through radioligand-binding studies performed in rat brain cerebral cortex membranes. SRIF-1 (also called SS-1) recognition sites were characterized by high affinity for SRIF-14 and SRIF-28, and for cyclic peptides such as octreotide and seglitide. In contrast, SRIF-2 (also called SS-2) sites exhibit high affinity for SRIF-14 and SRIF-28, but very low affinity for octreotide and seglitide. In fact, SS-1 and SS-2 binding sites correlate with recombinant SST_2_ and SST_1_ receptors, respectively (Hoyer et al., 1995b; Schoeffter et al., 1995).

Subsequently, five distinct receptor genes have been cloned and named chronologically according to their respective publication dates, but two were regrettably given the same appellation (SST_4_). In 1995, a consistent nomenclature for the recombinant receptors was agreed upon according to International Union of Basic and Clinical Pharmacology (IUPHAR) guidelines (sst_1_, sst_2_, sst_3_, sst_4_, and sst_5_) (Hoyer et al., 1995a). Given that radioligands could differentiate only two distinct SRIF binding sites, the subsequent cloning of five receptors was indeed surprising. IUPHAR guidelines recommended that recombinant receptors without well-defined functional characteristics should be referred to by lower case letters, i.e., sst_1_, sst_2_, etc. (Vanhoutte et al., 1996). When the recombinant receptor is shown to be of functional relevance in whole tissues and is fully characterized, upper case letters should be used, i.e., SST_1_, SST_2_, etc. (Vanhoutte et al., 1996). Moreover, the name of a receptor should not include the letter “R” or “r” as an abbreviation for receptor (Vanhoutte et al., 1996). Thus, according to IUPHAR guidelines, employing receptor names such as SSTR1, SSTR2, etc., is discouraged (Vanhoutte et al., 1996).

Shortly after cloning, it became apparent that the five recombinant receptors comprise two classes or groups, on the basis of their phylogeny, structural homologies, and pharmacological properties. One class was referred to as SRIF1, comprising SST_2_, SST_3_, and SST_5_ receptor subtypes. The other class was referred to as SRIF2, comprising the other two recombinant receptor subtypes SST_1_ and SST_4_ (Hoyer et al., 1995a). SST subtypes share many structural characteristics and their main intracellular signaling pathways. Conversely, individual SST subtypes can now clearly be differentiated according to their cellular and subcellular localization as well as distinct modes of regulation and functional and pharmacological properties. The IUPHAR Committee on Receptor Nomenclature and Drug Classification subcommittee now recognizes the physiologic correlates of SSTs and has decided on upper case nomenclature for all five SSTs. Thus, the new recommended nomenclature for SSTs is SST_1_, SST_2_, SST_3_, SST_4_, and SST_5_ (Alexander et al., 2017; Schulz et al., 2017).

### B. General Properties

All five SSTs are prototypical class A GPCRs that belong to the rhodopsin-like family of receptors. All possess seven transmembrane domains (TMDs) that provide the characteristic architecture of GPCRs. Receptor sequences for human SSTs range in length from 364 amino acids for SST_5_ to 418 amino acids for SST_3_ (Table 1). Unfortunately, crystal structures are not yet available for any SST. However, the five SST subtypes share common structural features such as a conserved sequence (YANSCANPILY) in transmembrane region 7 (mammalian SST signature). In addition, there is a consensus motif (X-[S/T]-X-Φ) at the end of the carboxyl-terminal tail of all mammalian SSTs. The X-S/T-X-Φ motif is regarded as a potential postsynaptic density protein (PSD)-95/discs large/ZO-1 (PDZ) domain binding site crucial for interaction with scaffolding proteins. Like all prototypical GPCRs, SSTs contain a DRY motif in the second intracellular loop (ICL) and are involved in coupling to G proteins. Genes encoding human SST_1_–SST_5_ are located on chromosomes 14, 17, 22, 20, and 16, respectively (Table 1). There is considerable sequence similarity between different SST subtypes (39%–57%) (Table 2). In fact, sequence similarity is high for a given subtype when compared across species (81%–98% for mouse, human, and rat homologs).

**TABLE 1:** Nomenclature and properties of somatostatin receptors

	SST_1_	SST_2_	SST_3_	SST_4_	SST_5_
Gennomic location	14q13	17q24	22q13.1	20p11.2	16p13.3
Amino acids	391	369	418	388	364
Naturally occurring agonists	SRIF-14, SRIF-28	SRIF-14, SRIF-28	SRIF-14, SRIF-28	SRIF-14, SRIF-28	SRIF-14, SRIF-28
	CST-17, CST-29	CST-17, CST-29	CST-17, CST-29	CST-17, CST-29	CST-17, CST-29
G protein coupling	G*α*_i/o_	G*α*_i/o_	G*α*_i/o_	G*α*_i/o_	G*α*_i/o_
Primary signal transduction	cAMP↓	cAMP↓	cAMP↓	cAMP↓	cAMP↓
	VOCC ↓	VOCC ↓	VOCC ↓	VOCC ↓	VOCC ↓
	GIRK↑	GIRK↑	GIRK↑	GIRK↑	GIRK↑
	NHE1↓	PTP↑	NHE1↓	NHE1↓	PTP↑
	PTP↑		PTP↑	PTP↑	
Expression in human normal tissue	Brain	Brain	Brain	Brain	
	Anterior pituitary Pancreatic islets	Anterior pituitary	Anterior pituitary	Retina	Anterior pituitary
	Gastrointestinal tract	Pancreatic islets	Pancreatic islets	Dorsal root ganglia	Pancreatic islets
		Dorsal root ganglia	Gastrointestinal tract	Placenta	Gastrointestinal tract
		Gastrointestinal tract	Lymphatic tissue		Lymphatic tissue
		Lymphatic tissue	Adrenals		Adrenals
		Adrenals			
Expression in human tumors*^a^*	GH-Adenomas NET	GH-Adenomas	GH-Adenomas		GH-Adenomas
		TSH-Adenomas	ACTH-Adenomas		ACTH-Adenomas
		NET	NF-Adenomas		NET
		Pheochromocytomas			
		Paragangliomas			
Phenotype of mice lacking receptor	Altered insulin homeostasis	High basal acid secretion	Impaired novel object recognition	Increased seizure susceptibility	Increased insulin secretion
		Inhibition of glucagon release		Increased anxiety	Basal hypoglycemia
		Impaired motor coordination			

^a^ Expression in >50% of cases.

**TABLE 2:** Sequences of human receptors were aligned using the BLAST algorithm, and the percentages of sequence identity (upper right) and similarity (i.e., the presence of similar amino acids; lower left) were determined Sequence comparisons are limited to the core regions of receptors (i.e., sequences encompassing the seven-helix bundle plus adjacent segments), whereas no significant similarities were detected in the N-terminal and C-terminal tails.

	SST_1_	SST_2_	SST_3_	SST_4_	SST_5_
SST_1_	100	55	52	69	49
SST_2_	74	100	53	53	56
SST_3_	69	69	100	48	56
SST_4_	82	73	66	100	53
SST_5_	64	74	69	70	100

Comparative genomic analysis suggests that the current set of receptors present in mammalian species arose from a single ancestral gene. This precursor was duplicated before the appearance of vertebrates, leading to genes coding for ancestral SRIF1-type and SRIF2-type receptors, and one gene coding for the ligand, SRIF (Ocampo Daza et al., 2012; Tostivint et al., 2014). Further tetraploidizations occurred during vertebrate evolution, generating genes coding for SST_2_, SST_3_, and SST_5_ from the SRIF1-type precursor gene, and genes coding for SST_1_, SST_4_, and SST_6_ from the SRIF2-type precursor gene. The gene coding for SST_6_ has been lost in mammals, but is identifiable in several fish species. An additional tetraploidization in teleost fish gave rise to even more receptor species (Ocampo Daza et al., 2012; Tostivint et al., 2014). The common ancestor also gave rise to two so-called Drostar receptors in *Drosophila* (Kreienkamp et al., 2002) that are not activated by known mammalian peptides (including SRIF variants and opioids). However, their endogenous ligand allatostatin C bears only superficial similarity to SRIF. In contrast, the signature motif YANSCANPILY present in mammalian receptors is only slightly modified to YSNSAVNPILY in Drostar1, and the C-terminal PDZ ligand motif found in all SSTs is also present in the fly (Kreienkamp et al., 2002).

Genes encoding SSTs are intronless within their coding sequence, except for SST_2_. The SST_2_ gene can be alternatively spliced to produce two receptor proteins, SST_2A_ and SST_2B_, that differ in length and sequence of their carboxyl termini. Human tissues contain the unspliced SST_2A_ variant exclusively, whereas both spliced forms have been identified in rodents (Vanetti et al., 1992). Although the SST_5_ gene does not contain CD introns, variants of SST_5_ mRNA formed by splicing of noncanonical donor and acceptor splice sites are identified in humans, pigs, and rodents (Durán-Prado et al., 2009). The human SST_5_ variants encode truncated receptors containing five (SST_5_TMD5) or four (SST_5_TMD4) transmembrane domains and distinct carboxyl-termini (Durán-Prado et al., 2009).

Despite the prominent therapeutic role of SST_2_- and SST_5_-targeting SRIF analogs in pharmacotherapy of endocrine tumors, surprisingly few disease-associated mutations have been identified in any of the seven genes comprising the SRIF system (two peptide precursors and five receptor genes). To date, it has been reported that a single acromegaly patient resistant to octreotide treatment displayed a coding polymorphism in SST_5_ that clearly affected receptor signaling (Ballare et al., 2001). The R240W mutation presumably disrupts G protein and mitogen-activated protein kinase (MAPK) signaling, abolishing the antisecretory effects of SRIF on SST_5_-expressing cells. Besides this unique case, loss of heterozygosity at SST_5_ was speculated to lead to reduced mRNA expression, but molecular mechanisms for this notion have not been conclusively elucidated (Lania et al., 2008). Although numerous studies have reported reduced SST_2_ and SST_5_ expression in treatment-resistant tumors, correlations with any particular polymorphism in SST genes have not been established. Molecular mechanisms underlying low SST expression in octreotide- or lanreotide-resistant tumors must therefore reside in genes outside of the SRIF system and still await identification.

Data from large-scale human sequencing studies, such as the exome aggregation consortium (which includes about 60,000 healthy individuals), further show that all five genes coding for SSTs are rather tolerant to sequence variations. In a ranking of about 18,000 human genes based on the presence of missense mutations, the genes coding for SST receptors are placed between positions 1459 (SST_1_) and 9488 (SST_5_), where 1 would be the gene that has the lowest tolerance for missense mutations (Lek et al., 2016).

The best-characterized action of SRIF is its strong inhibitory effect on both endocrine and exocrine cell secretion (Konturek et al., 1976; Dolais-Kitabgi et al., 1979; Mandarino et al., 1981). SRIF also inhibits neuronal excitability. All SST subtypes mediate inhibitory actions by association with G_i_/G_o_ proteins, members of the heterotrimeric guanine-nucleotide–binding protein family characterized by sensitivity to pertussis toxin (PTX) (Demchyshyn et al., 1993; Gu et al., 1995a,b; Gu and Schonbrunn, 1997; Carruthers et al., 1999). Activation of G_i_/G_o_ proteins by SSTs leads to suppression of two critical second messengers: cAMP and cytosolic Ca^2+^. The reduction in cAMP results from inhibition of adenylyl cyclase. SSTs act to inhibit calcium channels both directly and indirectly, the latter by opening G protein–activated inward-rectifier K channels (Kir3.x) to produce hyperpolarization and, as a consequence, inhibition of Ca^2+^ influx through voltage-operated calcium channels (VOCC) (Gromada et al., 2001). Reduction of either cAMP or cytosolic Ca^2+^ leads to inhibition of secretion, and the simultaneous reduction of both second messengers by SRIF results in synergistic inhibitory effects on hormone release. Signaling events responsible for inhibition of cell proliferation are less well understood than those that inhibit hormone secretion. One such pathway involves activation of protein tyrosine phosphatases (PTP) (Pan et al., 1992), including the Src homology region 2 domain-containing phosphatase (SHP)-1 and SHP-2. Ensuing dephosphorylation of specific substrates may counteract growth factor–stimulated tyrosine kinase activity and thus inhibit multiple mitogenic signaling pathways (Table 1).

Before subtype-specific antibodies became available, detailed mapping of receptor mRNA and binding sites had been described and comprehensively reviewed (Epelbaum et al., 1994; Patel, 1999; Dournaud et al., 2000). SSTs are widely expressed in the CNS and the endocrine system with some overlapping distributions, but different cellular and subcellular localizations (Piwko et al., 1996; Thoss et al., 1996a; Lanneau et al., 2000). Within the CNS, SSTs are mainly expressed on neurons in the cortex, hippocampus, amygdala, and hypothalamus (Perez et al., 1994; Perez and Hoyer, 1995; Thoss et al., 1996b; Hannon et al., 2002). In the endocrine system, SSTs are expressed on distinct cell populations in the anterior pituitary, pancreatic islets, adrenals, and neuroendocrine cells of the GIT. SSTs are also expressed on enteric ganglion and immune cells (Table 1).

All five SSTs bind the endogenous SRIF and CST ligands with high affinity. Many peptidic SRIF analogs have been developed, leading not only to the discovery of metabolically stable multireceptor SRIF analogs but also to subtype-selective receptor agonists and antagonists. Selective nonpeptide agonists and antagonists are now available for all five SSTs, except SST_4_, for which selective antagonists are still lacking (Table 1).

## IV. Somatostatin Receptor 1

### A. Somatostatin Receptor 1 Structure

Human SST_1_ was the first SST cDNA to be cloned by Yamada et al. (1992a). Cloning was accomplished from a pool of GPCR-like sequences amplified from human pancreatic islet RNA by reverse-transcriptase polymerase chain reaction using a generic set of primers covering highly conserved amino acid sequences in the third and sixth GPCR transmembrane segments (Libert et al., 1989). The mouse homolog was then obtained by screening a mouse genomic library with a human SST_1_ probe (Yamada et al., 1992a). There is 99% amino acid identity between human and mouse SST_1_ sequences. Meyerhof et al. (1991) had previously reported the sequence of a rat cDNA encoding a novel putative GPCR expressed primarily in the hypothalamus and cerebral cortex, but whose ligand could not be identified. The high identity of the predicted amino acid sequence of this orphan receptor (97% and 98% identity with human and mouse SST_1_, respectively) indicated that it corresponded to the rat homolog.

**Fig. 3.**
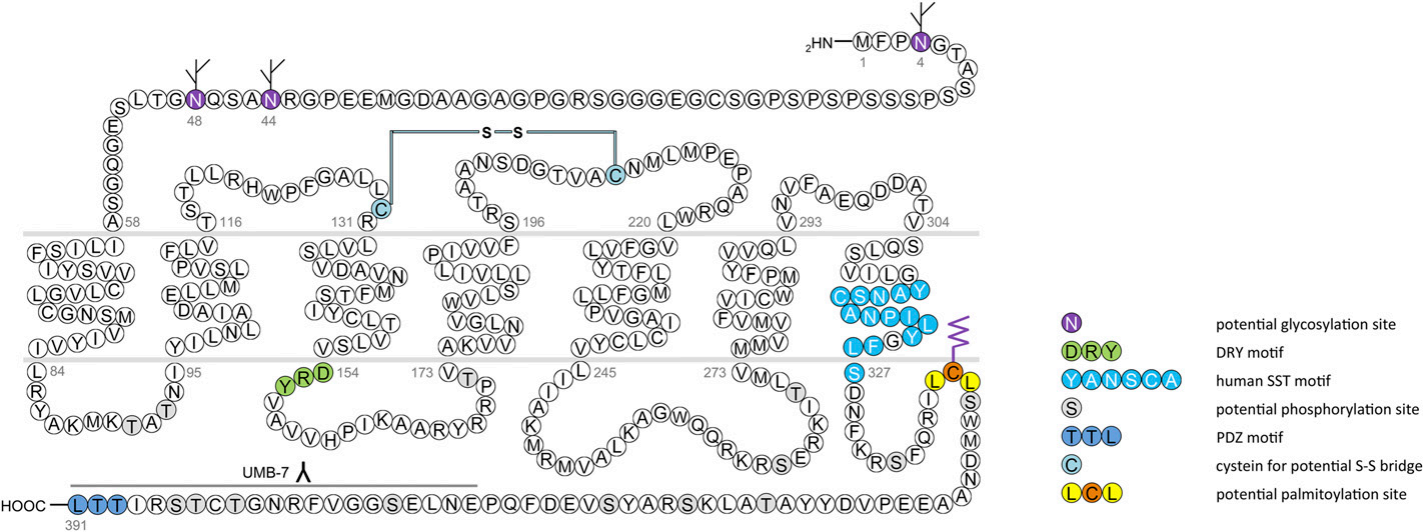
Structure of human SST_1_. The primary and secondary amino acid structure of the human SST_1_ (UniProtKB - P30872) is shown in a schematic serpentine format. Glycosylation sites are colored in purple; the DRY motif is highlighted in green; the human SST motif is in light blue; potential phosphorylation sites are in gray; the PDZ ligand motif is in dark blue; the disulfide-forming cysteines are in pale blue; and the potential palmitoylation site is in orange. UMB-7 is a rabbit monoclonal antibody, which detects the carboxyl-terminal tail of SST_1_ in a phosphorylation-independent manner.

In humans, SST_1_ is a 391-amino-acid protein encoded on chromosome 14q13 by an intronless gene whose promoter region, transcription start site, and 5′-untranslated region (UTR) have been elucidated (Fig. 3) (Redmann et al., 2007). Characterization of rabbit mAbs against SST_1_ (UMB-7) indicated that the protein is heavily glycosylated, migrating in SDS-PAGE at *M_r_* 45,000–60,000, but displaying the expected mol. wt. of *M_r_* 45,000 after protein extracts had been subjected to enzymatic deglycosylation (Lupp et al., 2013). The gene coding for mouse and rat SST_1_ is localized on chromosomes 12 C1 and 6q23, respectively, and both encode a 391-amino-acid protein.

### B. Somatostatin Receptor 1 Signaling Mechanisms

**Fig. 4.**
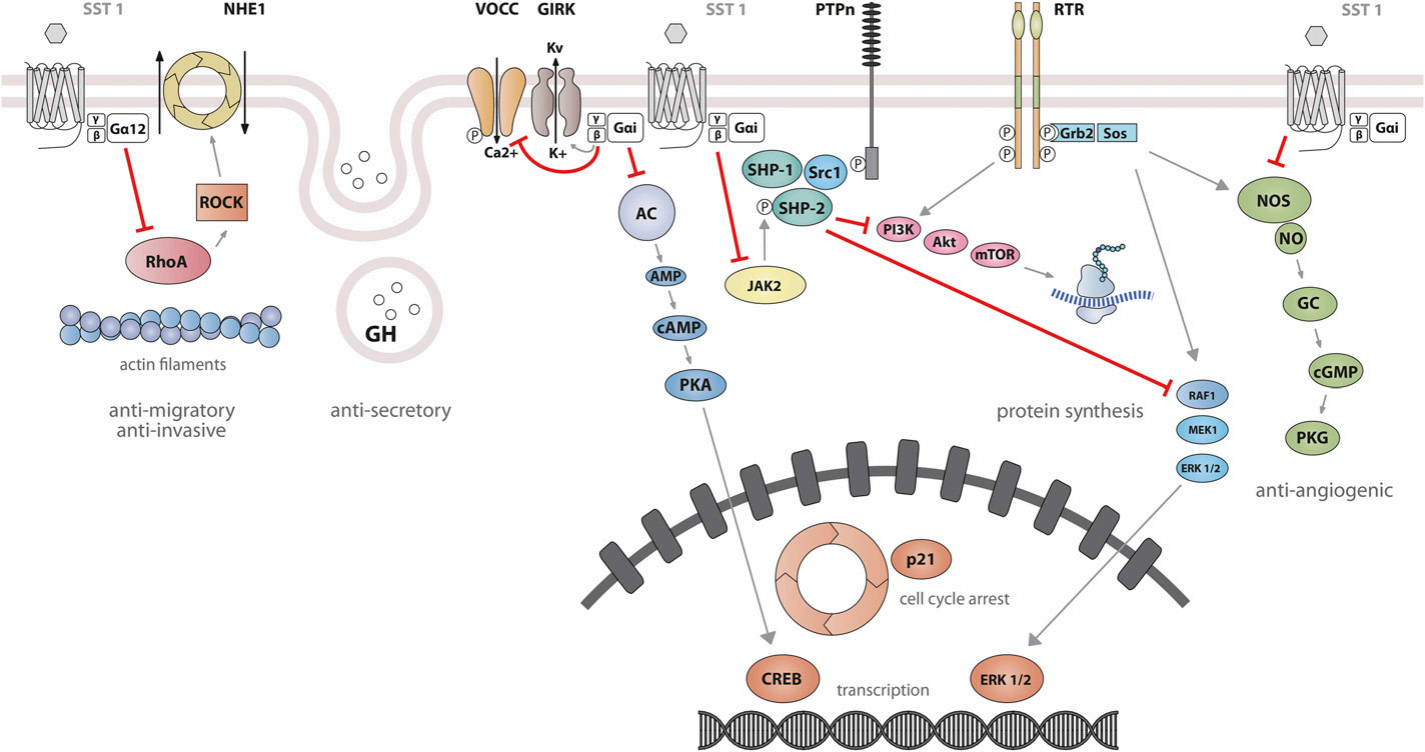
SST_1_ signaling leading to inhibition of hormone secretion, cell proliferation and migration, and angiogenesis. By coupling to Gi protein, SRIF-bound SST_1_ inhibits adenylate cyclase and reduces cAMP accumulation, as well as intracellular Ca^2+^ concentrations by regulating GIRK channels, which results in membrane hyperpolarization and subsequent reduction of Ca^2+^ influx through VOCC. This results in decreased hormone secretion. Inhibition of cell proliferation by SST_1_ involves upregulation of expression of the cyclin-dependent kinase inhibitor p21 (cip1/WAF1) and sequential activation through Src activity of tyrosine phosphatases (PTP*_η_* and SHP-2). Whereas p21 blocks cell cycling, tyrosine phosphatases block mitogenic signals through dephosphorylation (and inactivation) of effectors. Both PI3K–mTOR and MAPK pathways are inhibited, resulting in decreased cell growth and proliferation through inhibition of mRNA transcription and translation. SST_1_ also reduces endothelial NOS activation, resulting in reduced guanylate cyclase activity, cGMP production, and protein kinase G activity. Additionally, SST_1_ inhibits the NHE1 activity, resulting in a decrease of extracellular acidification rate. This involves inhibition of Rho activity through activation of G*_α_*_12_ protein by SST_1_.

Studies on SST_1_ signaling in various cell types have yielded heterogeneous results, depending on whether SST_1_ is expressed endogenously or heterologously by transfection, indicating that the cellular environment is important in determining the signal transduction machinery. Signal transduction studied in SST_1_ heterologous cell systems was first thought to be G protein-independent (Rens-Domiano et al., 1992; Buscail et al., 1994), but later shown to involve both PTX-sensitive and -insensitive G proteins (Garcia and Myers, 1994; Hadcock et al., 1994; Hershberger et al., 1994; Hou et al., 1994; Patel et al., 1994). Development of a polyclonal antibody to a 15-amino-acid peptide corresponding to a unique sequence in the SST_1_ carboxyl terminus has made it possible to immunoprecipitate endogenously expressed SST_1_ from pituitary tumor cell lysates and to demonstrate specific coupling to PTX-sensitive G proteins (Gu et al., 1995a). Gi proteins reported to couple to SST_1_ in SST_1_-transfected cells include Gi*_α_*_1,2,3_ (Hadcock et al., 1994; Kubota et al., 1994), although only Gi*_α_*_3_ dominantly couples SST_1_ to downstream adenylate cyclase inhibition (Kubota et al., 1994). SST_1_ transduces reduction of both cAMP accumulation and intracellular Ca^2+^ concentrations in heterologous cell systems (Fig. 4) (Garcia and Myers, 1994; Hadcock et al., 1994; Hershberger et al., 1994; Patel et al., 1994), as well as in insulinoma cells expressing SST_1_ endogenously (Roosterman et al., 1998). All SSTs regulate ion channels, including potassium channels (ATP-sensitive, inward, and delayed rectifying), as recently shown using an elegant fluorescence-based membrane potential assay in pituitary cells (Günther et al., 2016). SST_1_ activation results in membrane hyperpolarization and subsequent reduction of Ca^2+^ influx through voltage-sensitive Ca^2+^ channels, as demonstrated in endogenously SST_1_-expressing insulinoma cells (Roosterman et al., 1998). Interestingly, SST_1_, but not the other SSTs endogenously expressed in the mouse pancreatic *β*-cell line MIN6, shows exclusive coupling with N-type voltage-sensitive Ca^2+^ channels, resulting in reduced intracellular Ca^2+^ concentrations and in inhibition of insulin secretion (Smith, 2009). Such SST specificities were also observed in pituitary tumor cells, where, in contrast to SST_2_, SST_1_ fails to stimulate phosphoinositide-specific phospholipase C (PLC) activity or PLC-dependent release of Ca^2+^ from intracellular stores (Chen et al., 1997), but transduces inhibition of phospholipase A2 activity and arachidonic acid release, similar to SST_2_ (Cervia et al., 2002). Additionally, SST_1_ (like SST_3_ or SST_4_, but not SST_2_ or SST_5_) inhibits sodium/hydrogen exchanger 1 (NHE1) activity via a PTX-independent mechanism, as demonstrated in SST_1_-transfected cells (Hou et al., 1994), resulting in decreased extracellular acidification (Chen and Tashjian, 1999) that may be involved in inhibition of cell migration by SRIF (Buchan et al., 2002). Hence, SST_1_, but not SST_2_, attenuated rat sarcoma (Ras) homolog (Rho)–GTP levels and subsequent Rock activity induced both by GPCR or integrin activation when expressed in Chinese hamster lung fibroblast cells (CCL39), and these inhibitory effects correlated with decreased actin stress fiber assembly and cell migration (Buchan et al., 2002). Interestingly, a reported substrate of Rock is NHE1, which can serve as a plasma membrane-anchoring scaffold for actin filaments to control assembly of cortical stress fibers and focal adhesions. Because Rho inhibition by SST_1_ is PTX-independent, it may involve activation of G*_α_*_12_, a trimeric G*_α_* protein reported to inhibit both NHE1 (Lin et al., 1996) and the Rho guanine nucleotide exchange factor p115 RhoGEF (Hart et al., 1998). Notably, NHE1 localizes at the invadopodia (membrane structures involved in cell invasion) of human malignant breast carcinoma cells, where it generates extracellular acidification necessary for invadopodial-dependent extracellular matrix degradation and tumor invasion (Busco et al., 2010).

SRIF-induced increase of PTP activity (Hierowski et al., 1985; Liebow et al., 1989; Pan et al., 1992) was shown to be mainly involved in SRIF inhibitory effects on growth factor–stimulated cell growth (Buscail et al., 1994; Florio et al., 1994, 1996). PTP activity was found in a membrane complex containing SRIF and SSTs (Zeggari et al., 1994; Srikant and Shen, 1996). In pituitary tumor cells, SRIF-induced activation of protein phosphatases via PTX-sensitive G proteins (White et al., 1991; Duerson et al., 1996) correlates with endogenous SST_1_ expression (Florio et al., 1994). The rat membrane-associated protein tyrosine phosphatase *η* (PTP*_η_*) (homolog to human receptor tyrosine phosphatase type J, formerly known as DEP-1) transduces SRIF antiproliferative effects, in both insulin and/or TSH-treated thyroid PC C13 cells (which express all SSTs but predominantly SST_1_), and in glioblastoma cells (Massa et al., 2004; Barbieri et al., 2008), which express all five SSTs (Mawrin et al., 2004). In SST_1_-expressing heterologous cell systems, PTP-dependent inhibition of cell proliferation by SRIF was reported to rely on a complex interplay of different PTPs, comprising the receptor-like PTP*_η_*, which provides a long-lasting PTP activity (>2 hours), and the cytosolic SHP-2, which is rapidly activated (1–5 minutes) (Arena et al., 2007). SHP-2 and PTP*_η_* are sequentially activated in a complex comprising the Janus kinase 2 (JAK2) that phosphorylates and activates SHP-2, which in turn activates (by dephosphorylation) the SRC proto-oncogene, nonreceptor tyrosine kinase (Src) that tyrosine phosphorylates and activates PTP*_η_*. The latter phosphatase is directly responsible for SRIF-mediated inhibitory effect on fibroblast growth factor (FGF)–stimulated proliferation through SST_1_ (Arena et al., 2007). SRIF-activated PTPs inhibit cell proliferation by dephosphorylating tyrosine kinase receptors and/or downstream effectors, such as platelet-derived growth factor receptor, as demonstrated in SST_1_-expressing pancreatic cancer–associated fibroblasts (Duluc et al., 2015), or by inducing cell cycle arrest via upregulation of p21 (cip1/Waf1) expression in SST_1_-transfected cells (Florio et al., 1999). In addition to SST_3_, SST_1_ and SST_2_ blunt FGF-induced nitric oxide production through inhibition of endothelial nitric oxide synthase (NOS) in Chinese hamster ovary (CHO) cells in a PTX-dependent manner (Arena et al., 2005).

### C. Somatostatin Receptor 1 Regulation and Trafficking

Sequence analyses of the rat *Sstr1* gene promoter (Baumeister and Meyerhof, 1998, 2000a) demonstrated presence of putative transcription factor binding sites [GC box transcription factor, specificity protein 1, and activator protein (AP)-2] that are often found in TATA-less promoters (Smale et al., 1990). Presence of binding sites for tissue-specific transcriptional factors of the POU domain protein family (Rosenfeld, 1991) was also noted, including sites for pituitary-specific positive transcription factor 1 and POU family transcription factor Tst-1 that regulate tissue-specific rat *Sstr1* gene expression in the pituitary and in pancreatic *β*-cells, respectively (Baumeister and Meyerhof, 1998, 2000b). The porcine *Sstr1* gene promoter showed positive regulation by cAMP (through a CREBBP1 binding site) (Gahete et al., 2014), consistent with the cAMP-mediated upregulation of SST_1_ mRNA in rat pituitary primary cultures induced by GHRH treatment (Park et al., 2000), and in pituitary adenomas expressing a mutated G*_α_*_s_ (gsp oncogene) that constitutively activates the cAMP pathway (Kim et al., 2005). SST_1_ mouse pituitary expression may also be controlled by testosterone because pituitary SST_1_ mRNA levels are decreased in gonadectomized males but restored upon testosterone injection, and are increased by testosterone treatment in rat pituitary tumor cells (GH4C1 cells) (Xu et al., 1995a; Senaris et al., 1996). Such *Sstr1* gene regulation reported in mouse, rat, or pig was also confirmed for the human *SSTR1* gene (Redmann et al., 2007). Finally, the SST_1_ gene promoter contains two CpG islands (Redmann et al., 2007), putatively involved in head and neck squamous cell carcinoma tumorigenesis, where hypermethylation of the *SSTR1*, but also of SRIF, gene has been correlated with reduced disease-free survival (Misawa et al., 2015).

SRIF binding to its receptors results in internalization of receptor–ligand complexes, a critical process for receptor downregulation, resensitization, and signaling (Tulipano and Schulz, 2007). Intriguingly, SST internalization may differ across species, explaining controversial results reported for SST_1_. In the rat insulinoma cell line 1046-38, which endogenously expresses SST_1_, a recombinant rat epitope-labeled SST_1_ was expressed to demonstrate that SST_1_ endocytosis is observed upon cell treatment with SRIF (Roosterman et al., 1997). This was also confirmed in other rat SST_1_-expressing heterologous cells (Roth et al., 1997b). Interestingly, ligand-induced rat SST_1_ trafficking was dynamic, involving endocytosis followed by recycling, and then re-endocytosis of the receptor and of the intact and biologically active ligand, which are not directed to lysosomal degradation (Roosterman et al., 1997). In contrast, other studies showed that human SST_1_ expressed in heterologous cell systems demonstrates very slow, if any, internalization upon ligand binding (Stroh et al., 2000a; Liu and Schonbrunn, 2001), although it undergoes acute desensitization of adenylyl cyclase coupling that correlates with its phosphorylation status (Hukovic et al., 1996; Liu and Schonbrunn, 2001). Differences in rat and human SST_1_ internalization may be due to species-specific trafficking. Responsible for this species effect might be an amino acid change at a putative phospho-acceptor site (Thr^383^-Cys^384^-Thr^385^-Ser^386^) in the rat versus human SST_1_ C-terminal tail, where human Ser^386^ has been replaced by alanine in rat SST_1_. This substitution might explain the reported differences between rat and human SST_1_ affinity for *β*-arrestin-1 and subsequent trafficking (Tulipano et al., 2004; Ramirez et al., 2005). Confocal microscopy analyses showed bright immunoreactivity of both human and rat SST_1_ within the cytoplasm, both receptors accumulating the ligand (SRIF-14) into superficial compartments. Intriguingly, a fraction of SST_1_ stays clustered immediately beneath the plasma membrane, in as yet unidentified intracellular vesicular compartments (Nouel et al., 1997; Roosterman et al., 1997, 2007; Hukovic et al., 1999). This peculiar localization may be caused by the absence of a domain in the SST_1_ N terminus required for cell surface targeting, as described for SST_3_ (Ammon et al., 2002). The SST_1_ cytoplasmic pool serves as a reservoir for short-term upregulation of human SST_1_ expression at the membrane upon prolonged agonist treatment. Upregulation depends on phosphorylation events at the SST_1_ C-terminal tail (Hukovic et al., 1999). SST_1_ immunoreactivity is observed both at the membrane and in the cytoplasm in primary and heterologous cell models (Gahete et al., 2014), and in paraffin-embedded sections of diverse human tumor tissues, in contrast to SST_2_, which is predominantly membrane-associated (Hofland et al., 1999; Lupp et al., 2013).

### D. Somatostatin Receptor 1 Interacting Proteins

Unlike other SSTs, SST_1_ is not capable of homodimerization, prevented by structures within the C-terminal domain (Grant et al., 2004). SST_1_ was nevertheless found heterodimerized with SST_5_ in SST_1_- and SST_5_-coexpressing heterologous cell systems. Heterodimerization is induced by SST_5_- but not SST_1_-selective ligands and changes intracellular signaling (inhibition of forskolin-stimulated cAMP production) of the SST_1_/SST_5_ heterodimer as compared with SST_5_ homodimers or SST_1_ monomers (Grant et al., 2004). SST_1_ was also found heterodimerized with SST_2_ in prostate cancer cells; this complex stabilized with a bispecific (SST_1_/SST_2_) SRIF agonist, which nevertheless was less efficient than a mono-specific SST_1_ agonist to produce inhibition of cell proliferation (Ruscica et al., 2010). SST_1_, like other SSTs, harbors within its C terminus a PDZ-binding motif that interacts with membrane-associated guanylate kinase homologs, including PSD-95 and PSD-93 (Christenn et al., 2007), or synapse-associated protein SAP-97 (Cai et al., 2008), involved in SRIF signaling to regulate neuronal growth cone stability in neurons (including retraction of filopodia and lamellipodia). Whereas members of the membrane-associated guanylate kinase homolog subfamily are believed to play a role as molecular scaffolds in the organization of postsynaptic signaling machineries, SAP97 is also prominently expressed in axons and presynaptic terminals, where it may be involved in SST_1_ presynaptic functions (Cai et al., 2008).

### E. Somatostatin Receptor 1 Anatomic Framework

**Fig. 5.**
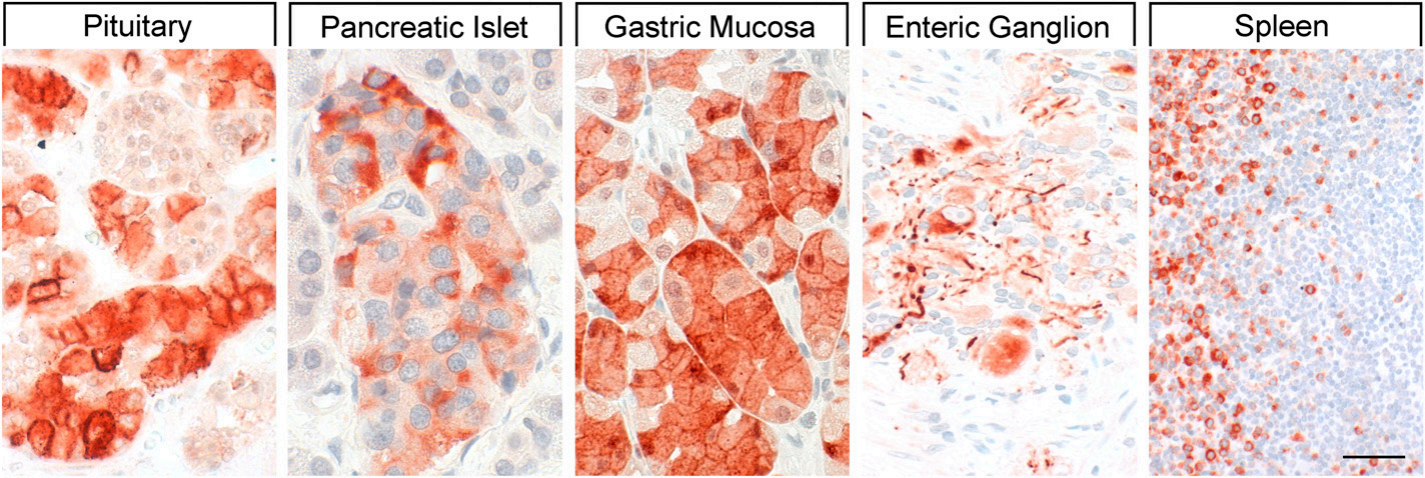
SST_1_ expression pattern in normal human tissues. Immunohistochemistry (red-brown color), counterstaining with hematoxylin; primary antibody: UMB-7; scale bar, 50 *µ*m. SST_1_ displays both membranous and cytoplasmic expression.

Binding studies using iodinated SRIFs in mice deficient for each of the SSTs suggest that SST_2_ is most abundant in the murine CNS (Hannon et al., 2002; Videau et al., 2003), although expression of other SST subtypes, including SST_1_, was confirmed by in situ hybridization in rat brain (Beaudet et al., 1995; Stumm et al., 2004). Immunohistochemistry studies demonstrated that SST_1_ is highly expressed in the hypothalamic paraventricular and ARC, the median eminence (Helboe et al., 1998; Hervieu and Emson, 1998; Stroh et al., 2006), as well as other brain regions, including basal ganglia, basal forebrain regions, and hippocampus (Schulz et al., 2000a). SST_1_ has also been localized in SRIF-containing amacrine cells of rat and rabbit retina (Dal Monte et al., 2003; Mastrodimou and Thermos, 2004). SST_1_ immunoreactivity is also intense in the spinal cord, especially in dorsal horn and dorsal medulla (Schulz et al., 2000a). Peripherally, SST_1_ is expressed in neurons of mouse, rat, and human dorsal root ganglia (DRG) (Bär et al., 2004; Imhof et al., 2011), and on intestinal mucosal nerve fibers (Van Op den Bosch et al., 2007). Outside the nervous system, high expression of human SST_1_ mRNA is apparent in stomach, intestine, and endocrine pancreas (Fig. 5) (Yamada et al., 1992a). Immunohistochemistry studies later confirmed that the SST_1_ protein is expressed mainly in these locations and also in the anterior pituitary (Portela-Gomes et al., 2000; Taniyama et al., 2005; Unger et al., 2012; Lambertini et al., 2013; Lupp et al., 2013). SST_1_ immunoreactivity is also positive in the parathyroid and bronchial glands (Taniyama et al., 2005), testis (staining in single cells between the tubules, resembling Leydig cells), and skeletal muscles (Unger et al., 2012). In the GI tract, SST_1_ is found expressed in stomach (Fig. 5), including antrum and corpus, in single cells resembling neuroendocrine or enterochromaffin-like cells (Unger et al., 2012), although expression in enterochromaffin cells (chromogranin-positive) was not always confirmed (Taniyama et al., 2005). Further SST_1_ expression was noted in the rectum (Taniyama et al., 2005) and also in enteric ganglion cells and corresponding nerve fibers and nerve terminals (Lupp et al., 2013). SST_1_ immunoreactivity was also detected in endothelial cells of blood vessels (Taniyama et al., 2005). In the immune system, SST_1_ protein is expressed in lymphocytes and macrophages (Taniyama et al., 2005), consistent with the positive SST_1_ immunoreactivity observed in cells resembling macrophages (cluster of differentiation 68 (CD68) positive) in spleen, lymph nodes, tonsils, thymus, lung, and gut mucosa, as well as in the stroma of diverse neoplasms (Lupp et al., 2013).

In neoplastic tissues, receptor autoradiography with SST_1_ subtype-selective ligands showed preferential expression of SST_1_ in prostate carcinomas and sarcomas (Reubi et al., 2001). Using the rabbit mAb UMB-7 (Table 3), SST_1_ expression was confirmed in prostate adenocarcinomas, rhabdomyosarcoma and liposarcoma, pituitary somatotroph adenomas, pancreatic adenocarcinoma, stomach cancer, urinary bladder cancer, pheochromocytoma, GI neuroendocrine tumors (NETs), breast carcinoma, cervix carcinoma, and ovarian tumors (Lupp et al., 2013). SST_1_ was abundantly expressed in bronchopulmonary NETs (Herrera-Martinez et al., 2017b) and positively associated with patient survival (Kaemmerer et al., 2015a).

**TABLE 3:** Rabbit monoclonal SST antibodies

	Clone	Epitope	Species Reactivity	Reference
SST_1_	UMB-7	^377^ENLESGGVFRNGTCTSRITTL^391^	Human	Lupp et al. (2013)
SST_2_	UMB-1	^355^ETQRTLLNGDLQTSI^369^	Mouse, rat human	Fischer et al. (2008)
SST_3_	UMB-5	^398^QLLPQEASTGEKSSTMRISYL^418^	Human	Lupp et al. (2012)
SST_5_	UMB-4	^344^QEATPPAHRAAANGLMQTSKL^364^	Human	Lupp et al. (2011)

### F. Somatostatin Receptor 1 Function

In the CNS, SST_1_ immunoreactivity is primarily found in fibers and terminals morphologically similar to varicose axons and that exhibit the highest brain SRIF immunoreactivity or are closely apposed by SRIF-immunoreactive fibers. This observation suggests that SST_1_ is predominantly targeted to presynaptic compartments (Schulz et al., 2000a). In this position it negatively modulates release of SRIF itself or of hypothalamic releasing and release-inhibiting hormones, including GHRH in ARC neurons, where high SST_1_ mRNA concentrations are found (Tannenbaum et al., 1998). Accordingly, SST_1_ was defined as an inhibitory autoreceptor located on the mediobasal hypothalamus, basal ganglia, and retina SRIF neurons (Thermos et al., 2006). Negative regulation of GHRH release by SRIF is consistent with the reported SST_1_ inhibitory role on hypothalamic regulation of GH secretion (Kreienkamp et al., 1999; Lanneau et al., 2000). Intriguingly, gender-related differences in both number and labeling density of SST_1_ mRNA-expressing cells are observed in the rat ARC (i.e., two- to threefold increase in males versus females) (Zhang et al., 1999). This observation may explain lower basal GH levels in male than in female mammals (Jansson et al., 1985), and also the sexually dimorphic GH pulsatile secretion (Low et al., 2001). More recently, the negative regulation by SRIF of GHRH neuron electrical activity was decrypted using a GHRH–GFP transgenic model (Osterstock et al., 2016). It revealed a sexual dimorphism, which is primarily attributable to a sex-dependent control of GABAergic and glutamatergic inputs by SRIF, rather than intrinsic differences in the GHRH neurons themselves. Interestingly, the positive glutaminergic neurotransmission onto GHRH neurons is an obligatory target of SRIF in female, providing a mechanism for a more tonic inhibition in female than in male, where this inhibitory signal was absent in one-third of animals. Intriguingly, this is the opposite for SRIF inhibition of GABAergic (negative) inputs, being especially robust and synchronized in males. Both SST_1_ and SST_2_ are involved in GHRH neuron rhythmicity, but SST_1_ receptors specifically transduce SRIF inhibitory control of GABAergic inputs, likely taking place at the presynaptic level (Osterstock et al., 2016). Seven percent of neuropeptide Y–positive neurons in the ARC coexpress SST_1_ mRNA, suggesting a direct interaction between the somatotropic axis and neuroendocrine regulatory loops of energy homeostasis (Fodor et al., 2005). Hypothalamic paraventricular and ARC SST_1_ may account for prevention of acute stress-induced gut motor functions in mice after central injection of a SST_1_-selective agonist, including inhibition of gastric emptying and stimulation of colonic motility (Stengel et al., 2011), putatively through central SRIFergic regulation of corticotropin-releasing factor (CRF) release and downstream stress-induced CRF actions (Stengel et al., 2013). Recently, neuroanatomical connections between somatostatin and kisspeptin neurones were observed in the rat ARC and ventromedial hypothalamus, where one-third of kisspeptin neurones exhibit SST_1_ immunoreactivity. Because kisspeptin is a gonadotropin-releasing hormone secretagogue, these observations suggest that the regulation of kisspeptin release by SST_1_ may at least be partly involved in the well-known inhibition of gonadotropin-releasing hormone release by SRIF (Dufourny et al., 2018). In the basal ganglia (substantia nigra, nucleus accumbens, globus pallidus, and ventral pallidum), SST_1_ is also present presynaptically, where it negatively regulates SRIF release (Vasilaki et al., 2004), strongly suggesting that it may serve as an autoreceptor to modulate systems regulated by SRIF (including dopamine). SST_1_ (together with SST_2_) are abundantly expressed in nerve processes of basal forebrain regions, including substantia innominata and the horizontal limb of the diagonal band (Hervieu and Emson, 1998), where SRIF inhibits glutamate release presynaptically through SST_1_, thereby regulating excitability of forebrain cholinergic neurons (Momiyama and Zaborszky, 2006). Expression and function of SST_1_ in the rodent hippocampus have been controversial until the demonstration of SST_1_-mediated SRIF inhibitory action on synaptic transmission, using hippocampal slices of SST_1_ KO mice and a SST_1_ selective agonist (Cammalleri et al., 2009). Hippocampal activity is regulated by SST_1_ through presynaptic inhibition of glutamate release induced by epileptiform treatment. In the spinal cord, SST_1_ may be involved in nociceptive transmission because dorsal horn and medulla regions coexpress SST_1_ together with SRIF, which has analgesic effects in rodents and humans (Malcangio, 2003; Imhof et al., 2011). In the peripheral nervous system, SST_1_ may be involved in inhibitory effects of SRIF on inflammation and nociception (Pinter et al., 2006), such as in mouse models of stress-related visceral nociception (Mulak et al., 2015) or immune-mediated arthritis (Imhof et al., 2011). In the retina, activation of SST_1_ with a selective ligand decreases SRIF release from retinal explants (Mastrodimou and Thermos, 2004). Surprisingly, loss of SST_1_ expression in SST_1_ KO mice results in upregulated SRIF and SST_2_ retinal expression, together with an enlargement of axonal terminals of rod bipolar cells, where SST_2_ is expressed, as well as enhanced SST_2_ function (Bigiani et al., 2004; Pavan et al., 2004). Conversely, in SST_2_ KO mice, SST_1_ expression is upregulated and rod bipolar cell axonal terminals are smaller (Casini et al., 2004). This suggests reciprocal inhibitory retinal roles of SST_1_ on SST_2_ expression, and vice versa. It certainly contributes to SRIFergic regulation of glutamatergic transmission along the vertical retinal visual pathway in which the SST_2_/SRIF receptor/ligand pair is probably restrained by SST_1_, consistent with reported SST_1_ autoreceptor functions (Dal Monte et al., 2003; Thermos et al., 2006).

In the periphery, SST_1_ is expressed, together with SST_5_ (Strowski et al., 2003), in a high percentage of pancreatic *β*-cells (Portela-Gomes et al., 2000), consistent with its reported role in regulating insulin secretion in studies using SST-selective agonists or in KO mouse models (Wang et al., 2004; Smith, 2009). In the anterior pituitary, the SST_1_-selective agonist CH-275 decreases GH secretion in wild-type, but not primary somatotroph cultures derived from SST_1_-KO mice (Kreienkamp et al., 1999), demonstrating the critical role for SST_1_ in regulating pituitary GH. SST_1_ was expressed in endothelial cells of normal human veins and arteries, including atherosclerotic arteries. SST_1_-selective agonists demonstrated consistent angio-inhibitory effects in vitro (Bocci et al., 2007) and induced vascular relaxation through cytoskeletal alterations (Liapakis et al., 1996), making SST_1_-specific analogs interesting for treatment of vascular diseases, including intimal hyperplasia. Intriguingly, another study localized SST_1_ mRNA and SST_1_ protein to vascular smooth muscle cells, where it showed acute upregulation of expression during vascular trauma coincidently with smooth muscle cell proliferation, making this receptor an interesting target to inhibit myointimal proliferation (Khare et al., 1999). The presence of SST_1_ in intestinal macrophages and mast cells, especially during inflammation, has been described in mice (Perez et al., 2003; Van Op den Bosch et al., 2007). Low SST_1_ expression was also found in macrophages differentiated from peripheral bone marrow cell–derived monocytes, where it mediates together with SST_2_ anti-inflammatory effects after activation by a multireceptor SRIF analog (Armani et al., 2007). During liver inflammation, such as in cirrhosis or hepatocellular carcinoma (HCC), all five SST mRNAs were expressed, whereas expression was not observed in normal human liver. The specific SST_1_ agonist L-797,591 was the only SST agonist to inhibit both liver cancer cell and hepatic stellate cell migration, making SST_1_ agonists putatively interesting to treat liver cirrhosis or HCC (Reynaert et al., 2004). Accordingly, SRIF also reduced production of collagens and inflammatory cytokines by hepatic stellate cells, although the specific receptor subtype was not identified. This putatively explains antifibrotic and immunomodulatory actions of SRIF in the liver (Lang et al., 2005; Reynaert et al., 2005). SST_1_ was also found uniquely expressed in stellate cells of pancreatic adenocarcinoma, and its activation by pasireotide reduced chemoprotective and prometastatic features of these fibroblastic cells by reducing IL-6 and collagen-1 secretion (Duluc et al., 2015; Moatassim-Billah et al., 2016).

SST_1_ is overexpressed in prostate cancer (Sinisi et al., 1997; Kosari et al., 2008) and mediates antiproliferative effects and inhibition of prostate-specific antigen release induced by the SST_1_-selective agonist BIM-23926 in prostate cancer cell lines (Pedraza-Arevalo et al., 2017). In the thymus, SST_1_ mRNA is expressed on isolated thymic epithelial cells, where SRIF inhibits proliferation (Ferone et al., 1999). SST_1_ mRNA is not expressed in fresh human or rat thymocytes (Sedqi et al., 1996; Ferone et al., 2002), but rat thymocyte activation with phytohemagglutinin or IL-1 selectively induced SST_1_ (Sedqi et al., 1996), suggesting SST_1_ involvement in thymocyte proliferation and differentiation.

### G. Somatostatin Receptor 1 Ligands

**Fig. 6.**
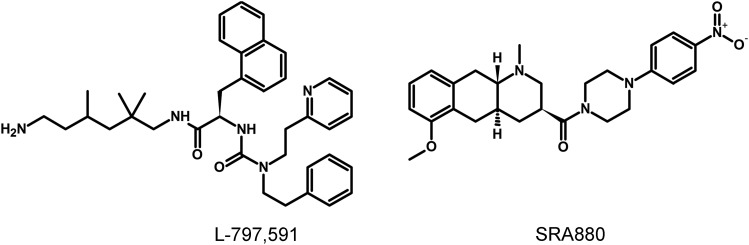
Structures of synthetic SST_1_ ligands. L-797,591, SST_1_ agonist; SRA880, SST_1_ antagonist.

The lack of available SRIF analogs with selectivity for SST_1_ stimulated the search for such compounds. The first to be identified is CH-275, which harbors a peptidic scaffold with selected amino acid deletions (des-aa1,2,5-SRIF) that in combination with DTrp at position 8, and 4-(N-isopropyl)-aminomethylphenylalanine (IAmp) at position 9, yields des-aa1,2,5-[DTrp8,IAmp9]-SRIF (CH-275), a SRIF agonist with nanomolar affinity for SST_1_, and that was 30-fold more selective for SST_1_ versus SST_2_/4/5 and 10-fold versus SST_3_, respectively (Liapakis et al., 1996). Using integrated combinatorial chemistry with high-throughput receptor-binding approaches, a SST_1_-selective nonpeptide compound (L-797,591) (Fig. 6; Table 4) displaying agonistic activity with an IC_50_ of 3 nM was the first pharmacological tool identified for selective SST_1_ in vitro and in vivo studies (Rohrer et al., 1998). BIM-23926 is a synthetic SST_1_-selective agonistic peptide (IC_50_ of 4 nM), reported to decrease cell viability of human medullary thyroid carcinoma TT cells, as well as to inhibit calcitonin release and cAMP levels (Fig. 6; Table 4) (Zatelli et al., 2002). SRA880 is the first reported nonpeptide SRIF SST_1_ competitive antagonist, with high affinity for both native and recombinantly expressed SST_1_ from various species (Fig. 6; Table 4) (rat, mouse, monkey, human), while displaying low affinity for a range of other neurotransmitter receptors, except the dopamine receptor D4 (Hoyer et al., 2004). The compound is bioavailable and brain penetrant. Consistent with the inhibitory autoreceptor role of SST_1_, SRA880 administration increases SRIF brain release and signaling, countering depressive-like symptoms in mice (Nilsson et al., 2012). A series of SST_1_-selective ergoline derivatives has been developed, some of which show effective oral bioavailability and brain penetration (Hurth et al., 2007; Troxler et al., 2008). The only clinically approved SRIF analog showing high affinity for SST_1_ is pasireotide, a nonselective peptidic compound displaying an IC_50_ of 9.3 nM for SST_1_, and IC_50_ values for SST_2_, SST_3_, and SST_5_ of 1, 1.5, and 0.16 nM, respectively (Schmid, 2008).

**TABLE 4:** Ligand-binding affinities of SST_1_-selective ligands

	SST_1_	SST_2_	SST_3_	SST_4_	SST_5_
L-797/591*^a^*	1.4	1875	2240	170	3600
BIM-23926*^b^*	3.6	>1000	>1000	833	788
SRA880*^c^*	7.6	>1000	>1000	>1000	954

^a^ Data from Rohrer et al. (1998).

^b^ Data from Zatelli et al. (2002).

^c^ Data from Hoyer et al. (2004), Cammalleri et al. (2009).

## V. Somatostatin Receptor 2

### A. Somatostatin Receptor 2 Structure

**Fig. 7.**
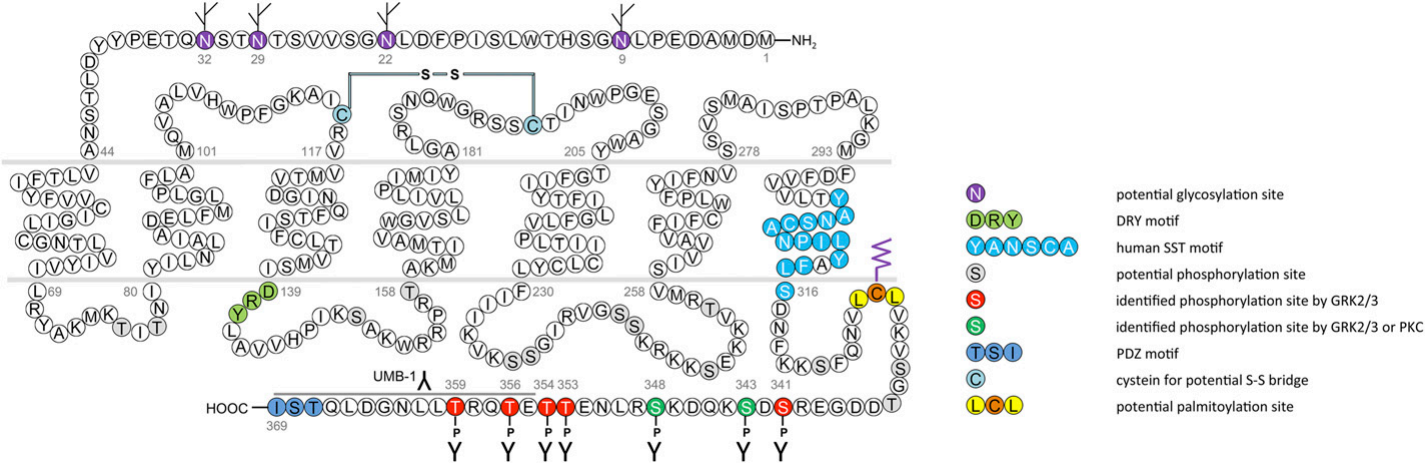
Structure of human SST_2_. The primary and secondary amino acid structure of the human SST_2_ (UniProtKB - P30874) is shown in a schematic serpentine format. Glycosylation sites are colored in purple; the DRY motif is highlighted in green; the human SST motif is in light blue; potential phosphorylation sites are in gray; identified GRK2/3 phosphorylation sites are in red; identified GRK2/3 or PKC phosphorylation sites are in dark green; the PDZ ligand motif is in dark blue; the disulfide-forming cysteines are in pale blue; and the potential palmitoylation site is in orange. UMB-1 is a rabbit monoclonal antibody, which detects the carboxyl-terminal tail of SST_2_ in a phosphorylation-independent manner.

cDNAs coding human and mouse SST_2_ were isolated, together with cDNAs coding for SST_1_, in early 1992 using a polymerase chain reaction–based approach with primers directed to regions conserved in all GPCRs (Yamada et al., 1992a). In the same year, a cDNA encoding for rat SST_2_ was identified by expression cloning from a rat brain cDNA library (Kluxen et al., 1992). The gene coding human SST_2_ is localized on chromosome 17q25.1 and consists of two exons. Whereas exon 1 contains only 5′UTR, the entire coding region and 3′UTR are located on exon 2. Genes encoding for mouse and rat SST_2_ are localized on chromosomes 11 E2 and 10q32.1, respectively. Homology between human and rodent SST_2_ is 94% (mouse) and 93% (rat) at the amino acid level, respectively. In all three species, SST_2_ is a 369-amino-acid protein (*M_r_* = 41,305 in humans), displaying typical seven-transmembrane segments and four putative N-glycosylation sites (Asn-9, Asn-22, Asn-29, and Asn-32) (Fig. 7). In Western blot experiments, the protein is detected as a characteristic smear migrating between 70 and 80 kDa, in keeping with the assumption that these Asn residues are extensively glycosylated. In addition to this long receptor species, a mouse SST_2_ splice variant codes for a shortened receptor with an alternative C-terminal tail (termed SST_2B_) (Vanetti et al., 1992). This variant arises due to removal of a part of the second exon that codes for the C-terminal tail of the long (SST_2A_) variant, and that also contains some 3′UTR. Some of the remaining 3′UTR of SST_2_A then becomes the coding region in SST_2B_ mRNA; in contrast to SST_2A_, the C-terminal sequence of SST_2B_ is very poorly conserved between species. Several functionally relevant elements of the SST_2A_ C terminus are lost due to the alternative splicing event: 1) elimination of phosphorylation sites that contribute to agonist-dependent desensitization and internalization and 2) SST_2A_ contains a C-terminal consensus motif for binding PDZ domains (Zitzer et al., 1999), which is not present in in SST_2_B. Whereas both spliced forms have been identified in rodents (Vanetti et al., 1992), human tissues exclusively contain the unspliced SST_2A_ variant. Consequently, we use the denomination SST_2_ for the long unspliced SST_2A_ variant throughout this review.

### B. Somatostatin Receptor 2 Signaling Mechanisms

**Fig. 8.**
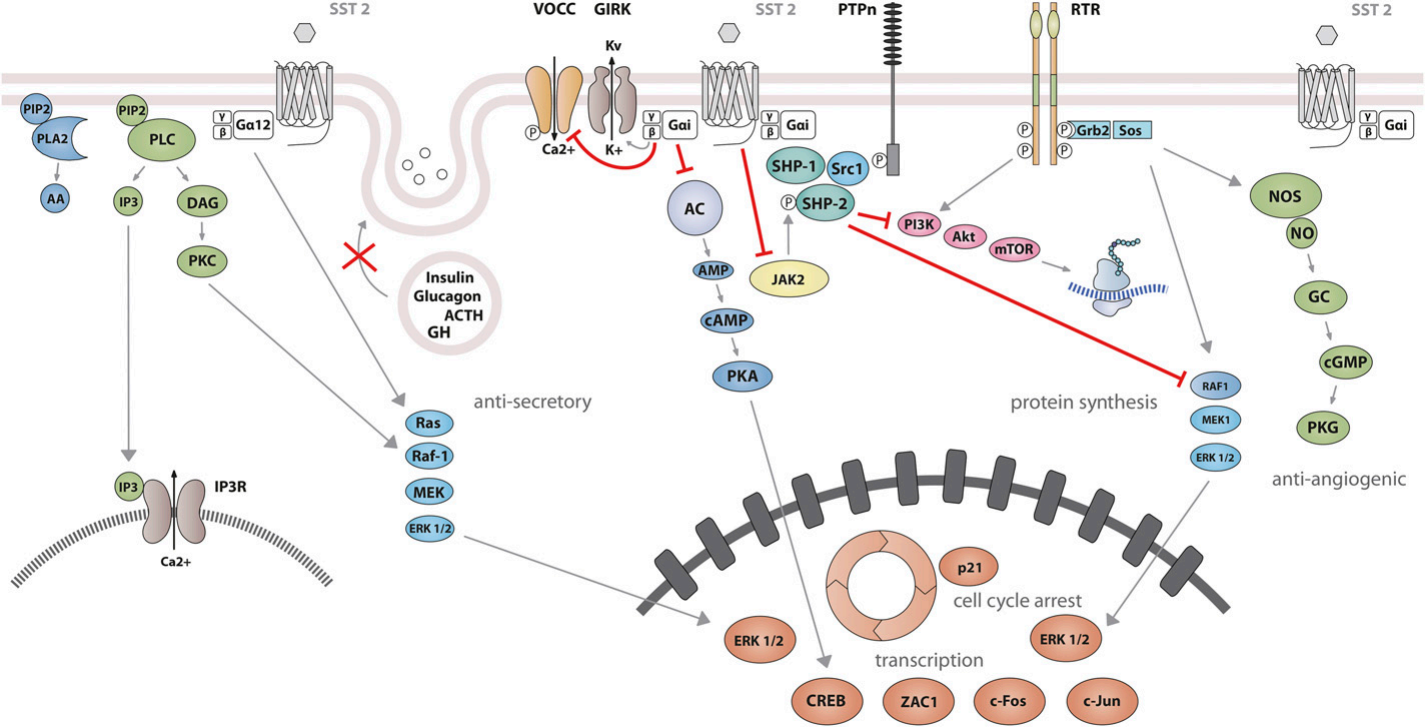
SST_2_ signaling leading to inhibition of hormone secretion, cell proliferation and migration, and angiogenesis. By coupling to Gi proteins, SST_2_ inhibits adenylate cyclase and reduces cAMP accumulation, and reduces intracellular Ca^2+^ concentrations by activating GIRK channels, which results in membrane hyperpolarization and subsequent reduction of Ca^2+^ influx through VOCC. This results in decreased hormone secretion. By coupling to a pertussis toxin–independent G protein, SST_2_ activates PLC, triggering inositol-1,4,5-trisphosphate (IP_3_) production and subsequent Ca^2+^ release into the cytoplasm from the endoplasmic reticulum. Major downstream effectors of SST_2_ are the tyrosine phosphatases SHP-1 and SHP-2 and the tyrosine kinase Src, which subsequently inhibit the PI3K-mTOR, MAPK, JAK2, and neuronal NOS pathways, thereby decreasing cell growth and proliferation. SST_2_-dependent inhibition of cell proliferation involves upregulation of the transcription factor ZAC1, triggering cell cycle inhibition.

Signaling properties of SST_2_ have been investigated in several heterologous expression systems, or in endogenous SST_2_-expressing cells using SST_2_ agonists. As physiologic actions of SRIF (e.g., on GH release) are sensitive to PTX (Cronin et al., 1983), unsurprisingly, SST_2_ also acts mostly through PTX-sensitive G proteins of the inhibitory/olfactory family of G*_α_* subunit (G_i/o_) type (Law et al., 1993), as reported in cultured mammalian cells heterologously expressing SST_2_, such as CHO, human embryonic kidney (HEK)293 cells, or SV4-transformed fibroblast-like derived monkey kidney (COS-7) cells. Major effects of SST_2_/G*_α_*_i/o_ signaling are inhibition of adenylyl cyclase, inhibition of voltage-gated calcium channels, and activation of K_ir_3.x (Fig. 8) (Kreienkamp et al., 1997). The inability of SRIF-bound SST_2_ to inhibit cAMP production in some cell lines (e.g., Law et al., 1993) was ascribed to lack of expression of an appropriate G protein (Hershberger et al., 1994). All of these effects are complementary for the inhibition of excitable cells, such as neurons or hormone-secreting cells. Thus, whereas hormone secretion by pituitary cells is driven by Ca^2+^ influx through voltage-gated Ca^2+^ channels, activation of potassium channels by SRIF-activated SST_2_ hyperpolarizes the membrane and prevents depolarization induced by hypothalamic releasing hormones. In pituitary cells, SST_2_ activates PLC [more specifically the PLC-*β*3 isozyme (Kim et al., 2012), which is partially blocked by PTX, putatively involving a G*_α_*_q/11_ (Chen et al., 1997) and/or G*_βγ_* protein (Kim et al., 2012)]. This results in inositol-1,4,5-trisphosphate formation and Ca^2+^ release into the cytoplasm from the endoplasmic reticulum, regulating the MAPK/extracellular signal-regulated kinase (ERK) pathway (Kim et al., 2012).

Besides these canonical G protein–mediated signaling pathways, SST_2_ also activates tyrosine phosphatase activity, associated with reduced serum-stimulated cell proliferation (Buscail et al., 1994; Reardon et al., 1997). This effect has been replicated in several cell types either overexpressing the receptor, or expressing lower endogenous levels of SST_2_ (Dent et al., 1997; Barbieri et al., 2008). Activation of tyrosine phosphatases by SRIF is PTX-sensitive and can be mimicked by addition of G*_α_*_i/o_ subunits purified from brain (Dent et al., 1997), implicating this G protein in the SST_2_-initiated signaling pathway. The nonreceptor tyrosine protein phosphatases SHP-1 and SHP-2, respectively, have emerged as major effectors (Lopez et al., 1997; Reardon et al., 1997), being copurified with SST_2_ (or SSTs in general) in heterologous cell systems, and sequentially activated, dependent on G protein (G*_α_*_i3_ and G*_βγ_*) and Src activity (Lopez et al., 1997; Ferjoux et al., 2003). Through phosphatase activation, SST_2_ inhibits tyrosine phosphorylation events such as those following activation of tyrosine kinase receptors (Bousquet et al., 1998; Hortala et al., 2003). This leads to cell cycle arrest and subsequent inhibition of cell proliferation, through upregulation of the cyclin-dependent kinase inhibitor p27/Kip1 (Pagès et al., 1999) and the zinc finger protein (Zac1) (Theodoropoulou et al., 2006), as mainly demonstrated in heterologous cell systems (Pagès et al., 1999; Grant et al., 2008). Furthermore, similar results were obtained using SST_2_-preferring or selective analogs in endogenously SST_2_-expressing cells [e.g., pituitary tumors (Ferrante et al., 2006; Hubina et al., 2006; Theodoropoulou et al., 2006; Horiguchi et al., 2009; Peverelli et al., 2017), insulinoma (Aoki et al., 2014), glioma (Barbieri et al., 2009), normal and tumoral pancreatic acinar cells (Charland et al., 2001), or thyroid cells (Medina et al., 1999)]. This involves regulation of several signaling pathways, including Ras/Raf/ERK (Dent et al., 1997; Lahlou et al., 2003), phosphatidylinositol-4,5-bisphosphate 3-kinase (PI3K)/AKT serine/threonine kinase 1 (AKT)/glycogen synthase kinase 3*β*/mechanistic target of rapamycin kinase (mTOR) (Bousquet et al., 2006; Theodoropoulou et al., 2006; Azar et al., 2008), p38 (Alderton et al., 2001), neuronal NOS (Lopez et al., 2001), and JAK2 (Hortala et al., 2003). Depending on phosphatase activity, SST_2_ activation also triggers apoptosis in endogenous SST_2_-expressing pituitary somatotroph tumor cells (Ben-Shlomo and Melmed, 2010), and also in pancreatic cancer cells engineered to express this receptor, where apoptosis is further stimulated by treatment with death ligands (Ben-Shlomo and Melmed, 2010). Finally, SST_2_-induced dephosphorylation events can also lead to inhibition of cell migration and invasion, through inhibition of the small G protein Rac and of the subsequent ruffle formation in endogenous SST_2_-expressing neuroblastoma cells (Pola et al., 2003). In addition, SST_2_-induced dephosphorylation events can lead to restoration of cell-to-cell (adherens and gap junctions) and cell-to-matrix (hemidesmosomes) contacts in SST_2_-transfected pancreatic cancer cells (Benali et al., 2000; Lahlou et al., 2005; Laval et al., 2014).

### C. Somatostatin Receptor 2 Regulation

Similar to genes encoding the other SSTs, genes coding SST_2_ from various species do not contain TATA and CAAT boxes (Greenwood et al., 1995). A minimal promoter fragment close to the transcription initiation site was identified as a novel initiator element sufficient to account for transcription from the SST_2_ promoter in neuroblastoma cells (Pscherer et al., 1996). Interestingly, a so-called enhancer box was identified in this region, which serves as a binding site for the basic helix-loop-helix transcription factor (SEF-2). Interaction of SEF-2 with the enhancer box was identified as a major driving force for activity of the promoter in several cell lines (Pscherer et al., 1996). Transcriptional activity is further enhanced by the binding of c-myc intron binding protein 1 (MIBP1), both to SEF-2 and to a thymine-cytosine–rich transcriptional enhancer element; the expression pattern of MIBP1 matches that of SST_2_ in the murine brain, suggesting that MIBP1 confers expression tissue specificity at least in the CNS (Dorflinger et al., 1999). In addition, negative regulatory elements have been identified in more distal regions of the promoter of the mouse *Sstr2* gene. Suppression of transcriptional activity by this region may be overcome by Smad3/Smad4, which plates the activity of the *Sstr2* gene under control of a transforming growth factor *β*–dependent signaling pathway (Puente et al., 2001). Finally, and relevant for tumors that escape SST_2_-mediated antiproliferative effect of SRIF and its analogs, an alternative 5*′*/upstream promoter was identified that may be silenced by methylation (Torrisani et al., 2008). High methylation levels in this region correlated with reduced SST_2_ expression in tumor cells (Shen et al., 2016).

Regulation of SST_2_ depends upon molecular mechanisms, implying phosphorylation events at the C-terminal tail followed by recruitment of *β*-arrestins and receptor endocytosis. Combined biochemical and mutagenesis approaches identified serine and threonine residues in the C-terminal tail of the SST_2_ that are phosphorylated upon SRIF and/or octreotide stimulation, namely, S341, S343, S348, T353, and T354 in rat SST_2_-transfected CHO and GH4C1 cells (Liu et al., 2009), and S341, S343, T353, T354, T356, and T359 in HEK293 cells stably transfected with rat or human SST_2_ (Nagel et al., 2011; Lehmann et al., 2014b). Agonist-dependent phosphorylation of the four threonine residues was also documented in rat pituitary tumor cells (GH3) transiently transfected with rat SST_2_, rat pancreatic insulinoma *β*-cells (INS1 cells), which endogenously express SST_2_, and rat pancreas in vivo (Poll et al., 2010). Although rat SST_2_ internalization was partially inhibited by mutation of threonine residues, none of the mutations resulted in a complete block of receptor internalization (Liu et al., 2008). By contrast, multisite phosphorylation of clusters of carboxyl-terminal serine and threonine residues of the human SST_2_ cytoplasmic tail is a critical event for receptor endocytosis (Lehmann et al., 2014b). Accordingly, in a SRIFoma, which synthesized and secreted SRIF and in which SST_2_ are localized intracellularly, receptors are phosphorylated, whereas in an ileal carcinoid tumor in which SST_2_ are membrane-bound, receptors are found nonphosphorylated (Liu et al., 2003). In human NET samples, SST_2_ phosphorylation is observed only in octreotide-treated patients and receptors are internalized, whereas in untreated tumors SST_2_ are not phosphorylated and are located at the cell membrane (Waser et al., 2012). Of note, unlike SRIF and octreotide, pasireotide (formerly known as SOM230) stimulates only phosphorylation of S341 and S343 residues of human SST_2_, followed by a partial receptor internalization (Lesche et al., 2009; Lehmann et al., 2014b). In cell lines, G protein–coupled receptor kinase (GRK_2_) (Liu et al., 2009) or GRK_3_ is involved in phosphorylation of S341 and S343 residues (Nagel et al., 2011), whereas the threonine residues (T353, T354, T356, and T359) are phosphorylated by GRK_2_ and GRK_3_ (Poll et al., 2010). In HEK293 cells stably expressing SST_2_, chemical protein phosphatase inhibitors and small interfering RNA knockdown screening lead to identification of protein phosphatase 1*β* (PP1*β*) as the GPCR phosphatase that catalyzes rapid dephosphorylation of residues T353, T354, T356, and T359 (Poll et al., 2011).

SST_2B_ terminates after residue 332 and therefore does not contain the phosphorylation sites identified in the C-terminal tail of SST_2_ (Cole and Schindler, 2000). Accordingly, SST_2B_ phosphorylation is not detectable after agonist stimulation of colonic adenocarcinoma cells, whereas SST_2_ is phosphorylated under the same conditions (Holliday et al., 2007). SRIF causes rapid desensitization of SST_2_, but not of SST_2B_, in this latter cell type. However, both receptor subtypes desensitized markedly in transfected CHO cell line subclone K1 (CHO-K1) cells (Cole and Schindler, 2000). Interestingly, phosphorylation in the third ICL of SST_2_, a sequence shared by the SST_2B_ variant, also occurs (Hipkin et al., 2000; Elberg et al., 2002), but does not play a role in internalization and desensitization (Lehmann et al., 2014b). Differences in receptor phosphorylation might be physiologically relevant in the rodent brain and the GIT, where different expression patterns of the two SST_2_ subtypes have been documented (Cole and Schindler, 2000).

The relationship between SST_2_ phosphorylation and *β*-arrestin binding, a major class of adaptor proteins involved in GPCR desensitization and internalization, has been investigated in different cell lines. In HEK293 (Tulipano et al., 2004) and CHO cells (Liu et al., 2005), or primary hippocampal neurons (Lelouvier et al., 2008), cotransfected with *β*-arrestin enhanced green fluorescent protein and the rat SST_2_, both *β*-arrestin-1 and *β*-arrestin-2 are recruited to the plasma membrane after agonist stimulation, form stable complexes with the receptor, and internalize together. *β*-arrestin-2 recruitment also occurs after agonist stimulation of human SST_2_ in HEK293 cells (Lehmann et al., 2014b). Together, these results suggest that the SST_2_ belongs to the class B GPCR subgroup, because its activation results in robust recruitment of both *β*-arrestin-1 and -2 (Oakley et al., 2000).

### D. Somatostatin Receptor 2 Trafficking

Studying SST trafficking has received increasing attention because the fate of internalized receptors, following agonist exposure, may vary from degradation to rapid recycling to the plasma membrane, thereby affecting responsiveness to endogenous ligands and drugs of therapeutic interest. A striking SST_2_ property is that in the vast majority of the cell types endogenously expressing this subtype, it is almost exclusively confined to the plasma membrane, such as in central and myenteric neurons, neuroendocrine cells of the gastric antrum (Gugger et al., 2004; Fischer et al., 2008), anterior pituitary (Fischer et al., 2008; Peineau et al., 2014), pancreatic islets, as well as central and peripheral tumors (Reubi et al., 2000b). However, intracytoplasmic SST_2_ localization was observed in CNS in regions exhibiting dense SRIF innervation such as the central nucleus of the amygdala (Dournaud et al., 1998). Intracellular localization was also described in a rat model of middle cerebral artery occlusion in cerebrocortical neurons adjacent to the infarct, which regionally correlates with transient SRIF depletion from axonal terminals (Stumm et al., 2004). Subcellular distribution of the receptor may be dependent on surrounding SRIF concentrations, as suggested in tumors of the nervous and neuroendocrine systems (pheochromocytomas and neuroblastomas) producing autocrine SRIF (Reubi et al., 2000b). Collectively, these studies highlight that SRIF released under physiologic or pathophysiological conditions regulates localization and trafficking of SST_2_ consistent with results obtained in cell lines (Csaba and Dournaud, 2001; Tulipano and Schulz, 2007; Jacobs and Schulz, 2008; Treppiedi et al., 2017). In hippocampal neuronal cells, SST_2_ trafficking was analyzed in detail at different times after acute intracerebral octreotide injections or in primary neuronal culture exposed to SRIF ligands (Csaba et al., 2001, 2007; Lelouvier et al., 2008; De Bundel et al., 2015). These experiments demonstrated for the first time that GPCR cargoes recycle through the trans-Golgi network (TGN) after endocytosis. After activation and internalization, endosomes bearing SST_2_ in dendrites (by far the major pool of SST_2_) and cell bodies fuse and migrate to a perinuclear compartment expressing *trans*-Golgi markers such as the integral protein of the TGN, TGN38, and syntaxin-6, but not *cis*-golgi markers such as cis-Golgi marker 130 (GM130). These results have been comfirmed in vivo by electron microscopy approaches that showed that SST_2_ cargoes were not targeted to degradative departments; rather, TGN-enriched receptors recycle to the plasma membrane (dendrites and cell bodies), where they are observed in preagonist challenge equivalent amounts. The recycling process, which depends on the length and extension of dendritic arborization, is slow, 3–6 hours in vitro and 24–48 hours in vivo. Differences in kinetics between in vivo and in vitro studies might be, at least in part, due to the persistence of the intracerebrally injected agonist, which cannot be removed or chased as in in vitro settings, implying several internalization/recycling cycles before total agonist clearance or degradation. Such trafficking of activated SST_2_ to the TGN was also reported in myenteric neurons, in which an intact TGN is necessary for receptor recycling (Zhao et al., 2013). In both DRGs and dorsal horn neurons, octreotide-activated SST_2_ in vivo also are observed to concentrate in perinuclear regions that resemble the TGN before recycling (24 hours) (Shi et al., 2014). The physiologic significance of this peculiar recycling pathway is not fully understood. Recycled receptor might undergo biochemical modifications and/or association with scaffolding proteins for proper delivery to the cell surface. An additional intriguing hypothesis is that SST_2_ targeted to the TGN could produce downstream cellular responses, such as coupling to different G proteins, as demonstrated for other GPCRs, the sphingosine 1-phosphate receptor (Mullershausen et al., 2009), and the TSH receptor (Calebiro et al., 2009).

Recently, studies have focused on factors involved in regulation of intracellular SST_2_ trafficking. Modulating recycling of a particular receptor can indeed impact its physiologic fate and therefore offer a potential therapeutic value. Using pharmacological and cell biologic approaches, it was demonstrated that leucyl-cysteinyl aminopeptidase (LNPEP; formerly known as insulin-regulated aminopeptidase) ligands accelerate recycling of internalized SST_2_ in neurons in vitro or in vivo (De Bundel et al., 2015). LNPEP, which shares common regional and subcellular distribution with internalized SST_2_, was shown to be involved in vesicular trafficking (Wright and Harding, 2011). Importantly, because LNPEP ligands increase the density of SST_2_ at the plasma membrane, they also potentiate SRIF-inhibitory effects on seizure activity (De Bundel et al., 2015). LNPEP therefore represents a therapeutic target for treatment of limbic seizures and possibly for other neurologic conditions in which downregulation of GPCRs occurs. In myenteric neurons, activated SST_2_ traffic to endothelin-converting enzyme 1 (ECE-1)–containing vesicles and TGN (Zhao et al., 2013). This endosomal peptidase degrades peptide ligands in intracellular organelles and promotes receptor resensitization (Roosterman et al., 2007). SST_2_ recycling (30 minutes) in myenteric neurons is dependent upon endosomal acidification, ECE-1 activity, and an ECE-1 cleavable ligand, which is the case for SRIF-14, but not for SRIF-28 or analogs, such as octreotide (Zhao et al., 2013). Hence, after activation by ECE-1–resistant SRIF-28 and analogs, SST_2_ remain within the TGN and are poorly recycled at 120 minutes. Assuming that SST_2_ signals in intracellular organelles, this could explain, at least in part, the long-lasting actions of SRIF analogs such as octreotide. Of note, although ECE-1 might be present in the hippocampus (Barnes et al., 1997), the kinetics of SST_2_ recycling were the same after activation by SRIF-14 or octreotide in hippocampal neurons (De Bundel et al., 2015), suggesting that the ECE-1 role on SST_2_ recycling is dependent upon cell types. Filamin A (FLNA), a scaffolding protein involved in intracellular trafficking of several transmembrane proteins (Onoprishvili et al., 2003; Noam et al., 2014), has been shown to interact with the SST_2_ in melanoma and pancreatic cell lines (Najib et al., 2012). FLNA appears crucial for SST_2_ stabilization and signaling at the plasma membrane (Peverelli et al., 2014; Vitali et al., 2016). In addition, FLNA may protect SST_2_ from degradation by facilitating targeting of the receptor to a recycling pathway during long-term agonist treatment of pancreatic tumor and GH-secreting tumor cells (Peverelli et al., 2014; Vitali et al., 2016).

### E. Somatostatin Receptor 2 Interacting Proteins

Interestingly, all SST subtypes (SST_1_–SST_5_; with the exception of the short-splice variant SST_2B_) contain a consensus motif for interaction with type I PDZ domains at the intracellular C termini. This motif is conserved throughout evolution, as the closest homologs of SSTs in Drosophila, the Drostar1 and Drostar2 receptors for type C allatostatins, also contain a PDZ ligand motif (Kreienkamp et al., 2002). In contrast, closely related opioid receptors are devoid of such a motif. In its simplest form, a type I PDZ ligand consists of the C-terminal sequence –S/T–X–*φ*–COOH, in which *φ* is a large hydrophobic residue (Phe in SST_4_; Ile or Leu in SST_1, 2, 3, 5_; Val in many other typical PDZ ligand motifs). Whereas such a motif is quite common, flanking sequences add to the specificity of PDZ-type interactions (Zeng et al., 2016), thus ensuring that not every PDZ ligand can interact with any PDZ domain. SST_2_ was the first SST for which an interaction with a PDZ domain–containing protein was reported; Zitzer et al. (1999) identified members of the Shank protein family as potential interactors through yeast two-hybrid screening (Zitzer et al., 1999). Shank proteins are important scaffold proteins of the PSD. They exhibit a complex domain structure, as the central PDZ domain is accompanied by a Ras association domain, a set of seven ankyrin repeats, and a sterile alpha motif (SAM domain) and a nuclear localization signals 1 domain. In addition, a long proline-rich stretch is involved in binding actin-binding proteins. In the PSD, Shank proteins are considered as master scaffold proteins that link receptor complexes to the actin-based cytoskeleton (Kreienkamp, 2008). As SST_2_ does not appear to be a postsynaptically enriched receptor, it appears likely that interactions between Shank proteins and SST_2_ are relevant at other, nonsynaptic sites. In further studies, PDZ domain–containing 1 (PDZ-K1) protein was identified as interaction partner for all SST subtypes, including SST_2_. As PDZ-K1 also interacts with a PLC isoform, this work suggests that PDZ-K1 allows for coupling of SST subtypes to PLC through ternary complex formation. Finally, C-terminal PDZ ligand motifs of a larger number of membrane proteins have been shown to promote postendocytic recycling through binding to the PDZ domain containing sorting nexin family member 27 (Steinberg et al., 2013), and the PDZ ligand of SST_2_ may also promote similar recycling.

Additionally, SST_2_ harbors two immunoreceptor tyrosine-based inhibition motif (ITIM) sequences (immunoreceptor tyrosine–based inhibitory motif: I/V/L/S-x-Y-x-x-L/V), present in the third ICL and C-terminal tail (Ferjoux et al., 2003). Such ITIM consensus sequences were initially found in inhibitory immunoreceptors (e.g., programmed cell death protein 1), triggering B cell receptor inhibition of SHP-2 recruitment, and subsequent dephosphorylation of B cell receptor effector molecules (Okazaki et al., 2001). Similarly, SRIF-induced phosphorylation of SST_2_ tyrosine 228 and 312 residues, present in each of the two SST_2_ ITIMs, triggers SHP-2 (but not SHP-1) direct recruitment to SST_2_, SHP-2 activation, and subsequent transduction of dephosphorylation events also involving the kinase Src and SHP-1, leading to cell proliferation inhibition (Ferjoux et al., 2003).

The first SST_2_ ICL contains two juxtaposed binding sites for the p85 regulatory subunit of PI3K and for the actin-binding and scaffolding protein FLNA (Bousquet et al., 2006; Najib et al., 2012). Depending on SST_2_ phosphorylation of tyrosine residues 66 and 71, present in FLNA and p85 binding sites, respectively, FNLA or p85 competitively binds to SST_2_ first ICL. In the absence of SRIF, these tyrosine residues are phosphorylated, enabling p85, but not FLNA, binding. This state is permissive for growth factor–induced activation of PI3K activity. In the presence of SRIF, SST_2_ tyrosine residues 66 and 71 are dephosphorylated, enabling FLNA, but not p85 binding, the dissociation of which from SST_2_ triggers PI3K inactivation. One hypothesis is that SHP-2 binding to SST_2_, induced by SRIF through ITIM phosphorylation on SST_2_ third ICL and C-terminal domain (Ferjoux et al., 2003), triggers dephosphorylation of FLNA and p85 binding sites in SST_2_ first intracellular domain. Alternatively, the scaffolding FLNA protein, once recruited onto SST_2_ in the presence of SRIF (Najib et al., 2012), brings SST_2_ in proximity to the phosphatase SHP-1, whose activity is critical to trigger SRIF-inhibitory effects on cell proliferation, migration, or invasion. Both rat and human SST_2_ form constitutive homodimers (Pfeiffer et al., 2001; Lehmann et al., 2016). The dynamics of ligand-induced trafficking have also been studied for pig SST_2_ (Durán-Prado et al., 2007). This receptor forms constitutive homodimers/multimers in the absence of ligand, which rapidly dissociate (11 seconds) upon SRIF binding. Interestingly, in contrast to human SST_2_, pig SST_2_ rapidly reassociates (110.5 seconds) during a subsequent process that temporally overlaps with receptor internalization (half-maximal 95.1 seconds) (Durán-Prado et al., 2007). When coexpressed heterologously, SST_2_ and SST_3_ form heterodimers with reduced SST_3_ activity (Pfeiffer et al., 2001). However, to what extent SST_2_ forms dimers or oligomers with other GPCRs in vivo is not known.

### F. Somatostatin Receptor 2 Anatomic Framework

#### 1. Central and Peripheral Nervous System

Two independent laboratories using two different antibodies directed toward the carboxy-terminal tail of the SST_2_ have demonstrated that this SST subtype is the most abundant SST in the rodent CNS (Schindler et al., 1997; Dournaud et al., 1996) in agreement with both in situ hybridization experiments and autoradiographical studies using SST_2_-preferring ligands. Strong SST_2_ labeling is detected in the deep layers of the cerebral cortex, CA1 field, and dentate gyrus of the hippocampus, lateral septum, medial septum/diagonal band of Broca, medial habenula, bed nucleus of the stria terminalis, endopiriform nucleus, claustrum, amygdaloid complex, locus coeruleus, and nucleus tractus solitarius. In the hypothalamus, the highest densitiy of SST_2_ immunoreactivivity is located in the ARC and the medial tuberal nucleus as well as in the lateroanterior nucleus and the ventrocaudal part of the tuber cinereum (Csaba et al., 2003). In the rat spinal cord, SST_2_ neurons are localized in the superficial layers of the dorsal horn (Schindler et al., 1997, 1998a; Schulz et al., 1998b,c; Segond von Banchet et al., 1999) often closely apposed by SRIF-immunoreactive terminals (Schulz et al., 1998b). In the rat retina, several neuronal cell types express SST_2_. In the outer layers, immunoreactivity is localized to cone photoreceptors, horizontal cells, and rod and cone bipolar cells. In the inner layers, SST_2_ immunostaining is present in numerous medium- to large-size amacrine cells (Johnson et al., 1999). Regional distribution of SST_2_ immunostaining in the human CNS is generally congruent with that reported for the rat, although, in contrast to rodents, human cerebellum displayed significant SST_2_ immunostaining (Schindler et al., 1998b; Csaba et al., 2005; Shi et al., 2014).

In the rat peripheral nervous system, medium-size neurons distinct from those expressing SRIF display SST_2_ in the DRG (Schulz et al., 1998b). In the human and rat GIT, SST_2_ are localized in neurons of the myenteric and submucosal plexuses, and in fibers distributed to the muscle, mucosa, and vasculature (Sternini et al., 1997; Reubi et al., 1999).

Immunohistochemical experiments have examined localization of SST_2B_ (Schulz et al., 1998a; Schindler et al., 1999). In the rat brain, somatodendritic labeling is evident in several regions that also exhibit SST_2_ immunostaining, including the olfactory bulb, cerebral cortex, hippocampal formation, septal nuclei, and superior colliculi. In contrast to SST_2_, the Purkinje cell layer of the cerebellum appears to be SST_2B_ immunoreactive. In the rat spinal cord, whereas the SST_2_ is confined to the superficial layers, SST_2B_ is located in neuronal perikarya and proximal dendrites throughout the gray matter of the spinal cord (Schulz et al., 1998a).

#### 2. Pituitary

**Fig. 9.**
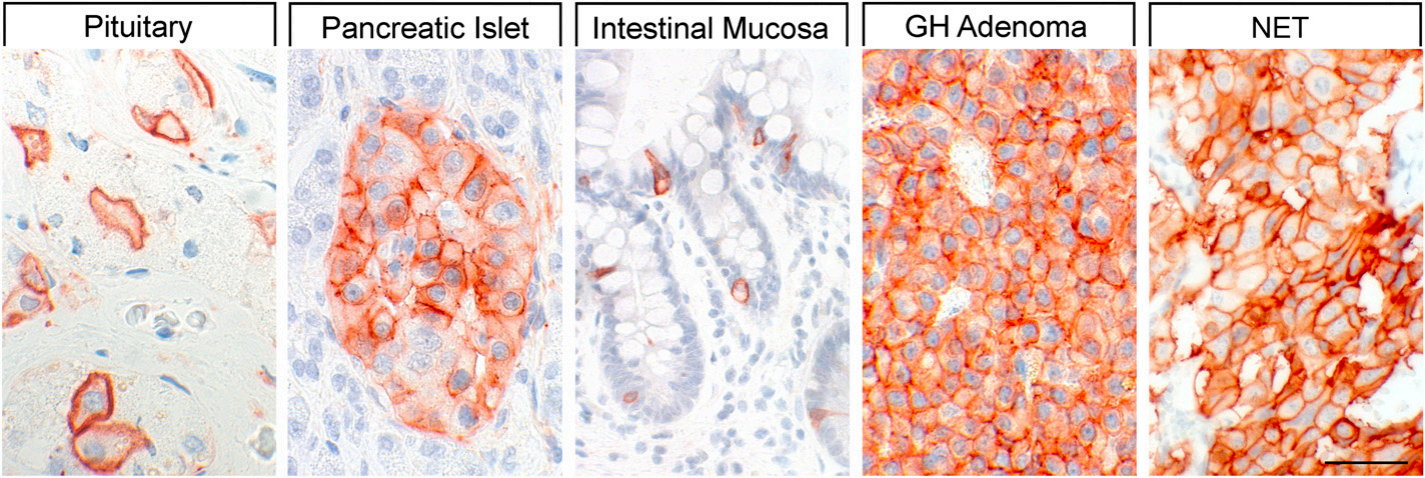
SST_2_ expression pattern in normal human and neoplastic tissues. Immunohistochemistry (red-brown color), counterstaining with hematoxylin; primary antibody: UMB-1; scale bar, 50 *µ*m. Note that SST_2_ is predominantly expressed at the plasma membrane.

Using specific antibodies against SST_2_, it appears that this receptor is largely distributed in the adult rat and human pituitary (Fig. 9) (Mezey et al., 1998; Peineau et al., 2014). Although all anterior pituitary cell types express the SST_2_ protein, GH-expressing cells almost completely colocalize with the SST_2_, whereas 50% of gonadotrophs, 60% of corticotrophs, 30% of thyrotrophs, and 10% of prolactin cells exhibit SST_2_ immunoreactivity (Peineau et al., 2014). Of note, some discrepancies with distribution of the mRNA exist because the SST_2_ mRNA was previously found in 40% of somatotrophs, 36% of thyrotrophs, 26% of lactotrophs, 3% of corticotrophs, and 8% of gonadotrophs (Day et al., 1995).

#### 3. Peripheral Organs

Immunohistochemical studies also revealed the presence of the SST_2_ in striated ducts of the parotid gland, in neuroendocrine and enterochromaffin-like cells of the GI mucosa, in enteric ganglia, in insulin- and glucagon-secreting cells of the pancreas (Fig. 9), in the reticular zone of the adrenal cortex, in glomeruli and tubules of the kidney, in luteinized granulosa cells of the ovary, in basal parts of testicular tubules, in granulocytopoietic cells of the bone marrow, in alveolar macrophages of the lung, and in germinal centers of lymph follicles (Fischer et al., 2008; Lupp et al., 2011; Unger et al., 2012; Stollberg et al., 2016). All in all, SST_2_ was the most frequently detected SST subtype, and, in most cases, SST_2_ predominantly immune-stained at the cell plasma membrane.

#### 4. Tumors

As determined using the rabbit mAb UMB-1, SST_2_ also represents the most prominent SST subtype in tumor tissues, with only few exceptions, such as pituitary adenomas (Fischer et al., 2008). In somatotroph and thyrotroph pituitary adenomas, SST_2_ is present at high abundance in >80% of tumor specimens, along with a high presence of SST_5_. In contrast, in gonadotroph, corticotroph, and nonfunctioning pituitary adenomas where SST_2_ expression was low or even absent, a preponderance of SST_3_ or SST_5_ has been noted (Fischer et al., 2008; Lupp et al., 2011). In brain tumors, the prevalence of SST_2_ was highest. SST_2_ was present in most of meningiomas (Schulz et al., 2000b; Fischer et al., 2008), medulloblastomas (Guyotat et al., 2001; Cervera et al., 2002; Remke et al., 2013), neuroblastomas (Albers et al., 2000), and supratentorial primitive neuroectodermal tumors of childhood (Fruhwald et al., 2004). SST_2_ was also detected in oligodendrogliomas, but much less frequently in astrocytomas (Cervera et al., 2002; Kiviniemi et al., 2017). Furthermore, noticeable SST_2_ expression occurred in peripheral nerve sheath tumors, especially in schwannomas (Mawrin et al., 2005).

SST_2_ was present in 43%–66% of medullary as well as in papillary and follicular thyroid carcinomas (Papotti et al., 2001; Druckenthaner et al., 2007; Mussig et al., 2012; Pazaitou-Panayiotou et al., 2012; Atkinson et al., 2013; Woelfl et al., 2014; Herac et al., 2016). SST_2_ was also detected in a high percentage (>70%) of pheochromocytomas and paragangliomas (Fischer et al., 2008; Lupp et al., 2011; Saveanu et al., 2011; Elston et al., 2015) as well as in 33% of both functioning and nonfunctioning adrenocortical adenomas (Unger et al., 2008). Very low levels of SST_2_ were also observed in lymphomas (Dalm et al., 2004; Stollberg et al., 2016; Ruuska et al., 2018). SST_2_ represents by far the most prominent SST subtype detected in gastroenteropancreatic (GEP)-NETs, and, overall, it was identified in >70% of cases at a high expression intensity (Kulaksiz et al., 2002; Fischer et al., 2008; Corleto et al., 2009; Srirajaskanthan et al., 2009; Zamora et al., 2010; Lupp et al., 2011; Okuwaki et al., 2013; Kaemmerer et al., 2015b; Mehta et al., 2015; Qian et al., 2016; Wang et al., 2017). However, SST_2_ was detected more frequently in gastrinomas (100%) and in carcinoid tumors (86%) (Fig. 9) than in insulinomas (58%) (Kulaksiz et al., 2002). SST_2_ expression was also found to be higher in functioning than in nonfunctioning tumors (Zamora et al., 2010; Song et al., 2016) and more pronounced in gastroenteric than in pancreatic neoplasms (Zamora et al., 2010). Negative correlation between SST_2_ expression and tumor grading or proliferation rate and a positive association with patient outcomes have been shown (Corleto et al., 2009; Srirajaskanthan et al., 2009; Zamora et al., 2010; Okuwaki et al., 2013; Kaemmerer et al., 2015b; Mehta et al., 2015; Qian et al., 2016; Song et al., 2016; Wang et al., 2017). Furthermore, a positive correlation between SST_2_ expression and SST-based imaging was demonstrated (Srirajaskanthan et al., 2009; Diakatou et al., 2015; Kaemmerer et al., 2015b). SST_2_ was detected in 32%–56% of bronchopulmonary NETs. However, in comparison with GEP-NETs, SST_2_ expression was less pronounced. Lower SST_2_ expression in high-grade in comparison with low-grade tumors and a positive correlation with SST-based imaging were shown (Righi et al., 2010; Kaemmerer et al., 2015a; Lapa et al., 2016). SST_2_ expression was also observed in NETs of other origins, including thymus, breast, cervix, or prostate (Kajiwara et al., 2009; Mizutani et al., 2012). Furthermore, SST_2_ was detected in 88%–100% of GI stromal tumors (GIST), and, also in this tumor entity, an association with favorable patient outcomes was demonstrated (Palmieri et al., 2007; Arne et al., 2013; Zhao et al., 2014). Depending on tumor grade and location, SST_2_ was observed in 45%–100% of colorectal carcinomas (Qiu et al., 2006; Evangelou et al., 2012) and in 41%–67% of HCCs (Blaker et al., 2004; Reynaert et al., 2004; Verhoef et al., 2008); SST_2_ was expressed in 20%–79% of breast cancers (Pilichowska et al., 2000; Orlando et al., 2004; Kumar et al., 2005; Fischer et al., 2008; Lupp et al., 2011; Frati et al., 2014), in 57% of cervical carcinomas, in 39% of endometrial cancers (Schulz et al., 2003), and in 30% of ovarian carcinomas (Hall et al., 2002; Schulz et al., 2003). Furthermore, a moderate to strong SST_2_ expression was observed in 13% of prostate cancers in general and in 50% of prostate cancers with endocrine differentiation (Matei et al., 2012; Hennigs et al., 2014). SST_2_ was detected in 59% of Merkel cell carcinomas (Gardair et al., 2015) and in melanomas (Ardjomand et al., 2003; Valsecchi et al., 2013). Finally, SST_2_ expression in normal exocrine pancreatic tissue is progressively lost during pancreatic ductal adenocarcinoma progression (Buscail et al., 1996; Laklai et al., 2009), which participates in tumor aggression, as demonstrated in mouse models of pancreatic cancer combined with SST_2_ KO mice (Chalabi-Dchar et al., 2015). Accordingly, in vitro and in vivo re-expression of SST_2_ in human pancreatic cancer cell lines through SST_2_ cDNA transfection (Delesque et al., 1997; Guillermet et al., 2003; Laval et al., 2014) and through in vivo SST_2_ gene transfer in mouse models (Vernejoul et al., 2002) and in a first-in-man phase I clinical trial (Buscail et al., 2015), respectively, demonstrated promising oncosuppressive activity in advanced pancreatic cancer.

### G. Somatostatin Receptor 2 Function

#### 1. Endocrine System

SRIF was originally described as an inhibitor of GH release, but it also inhibits secretion of other pituitary hormones. SST_2_ is predominantly responsible for regulation of physiologic secretion of GH and TSH (Ben-Shlomo and Melmed, 2010) indirectly mediated by opening of K^+^ channels. The subsequent K^+^-derived membrane hyperpolarization and reduction of L- and N-type Ca^2+^ influx as well as intracellular Ca^2+^ concentration are major mechanisms by which SRIF, through the SST_2_ subtype, acutely inhibits exocytosis of hormone-containing vesicles (Ben-Shlomo and Melmed, 2010). SST_2_ also inhibits exocytosis of hormone-containing vesicles derived from pancreatic *α*- and *β*-cells. In rodents secretion of glucagon and in humans secretion of both glucagon and insulin are regulated by SST_2_ (Singh et al., 2007; Strowski and Blake, 2008; Kailey et al., 2012). In addition, the SST_2_ subtype activates K_ir_3.x, which leads to hyperpolarization and inhibits voltage-gated P/Q-type Ca^2+^ channels (Kailey et al., 2012).

#### 2. Central Nervous System

##### a. Neuronal excitability and epilepsy

Another major role of SST_2_ is inhibitory neuromodulation. Effects of SST_2_ on neuronal excitability have been studied in several CNS cell populations. Activation of SST_2_ in medial septal GABAergic neurons results in decreased discharge rate and consequent reduction of hippocampal *θ* rhythm power (Bassant et al., 2005). SST_2_ also mediates hyperpolarization of dorsal horn neurons and subsequent antinociceptive effects (Song et al., 2002; Yin et al., 2009; Shi et al., 2014). In the ventrolateral medulla, SST_2_ activation of presympathetic neurons provokes robust sympathoinhibition with bradycardia and hypotension (Burke et al., 2008). Rodent and sheep gonadotropin-releasing hormone neurons are inhibited by SST_2_, which results in decreased luteinizing hormone secretion (Bhattarai et al., 2010; McCosh et al., 2017). Activation of SST_2_ in olfactory bulb mitral cells modulates dendrodendritic inhibition between mitral and granule cells, which in turn results in increased *γ* oscillation power of mitral cells and increased odor discrimination performances (Lepousez et al., 2010). The role of SRIF in neuronal excitability has been mostly studied in the hippocampal formation. In CA1 pyramidal neurons, SRIF has hyperpolarizing effects through activation of K^+^ channels (Moore et al., 1988; Schweitzer et al., 1990, 1998; Tallent and Siggins, 1997).

Postsynaptic hyperpolarization of CA3 pyramidal neurons by SRIF has also been demonstrated (Tallent and Siggins, 1999). Presynaptic inhibition of glutamate release by SRIF reduces excitatory synaptic input on CA1 neurons (Kozhemyakin et al., 2013); thus, SRIF decreases both post- and presynaptic hippocampal pyramidal cell excitability (Tallent and Qiu, 2008). By contrast, in the dentate gyrus, SRIF has no effect on granule cell postsynaptic currents or firing properties. SRIF, however, inhibits postsynaptic N-type Ca^2+^ channels in granule cells, resulting in inhibition of long-term potentiation (Baratta et al., 2002), a form of synaptic plasticity, critical in learning and memory, and also plays an important role in epileptogenesis. Converging evidence suggests that, of the five SSTs, SST_2_ exerts a predominant role in transduction of SRIF actions in the hippocampal formation (Csaba et al., 2004, 2005). SRIF plays a prominent role in epilepsy in agreement with its inhibitory neuromodulatory function, and SST_2_ mediates most of antiepileptic actions of SRIF in rats (Vezzani and Hoyer, 1999; Binaschi et al., 2003; Baraban and Tallent, 2004; Cervia and Bagnoli, 2007; Tallent and Qiu, 2008; Viollet et al., 2008), likely in humans (Csaba et al., 2005), but not in mice (Moneta et al., 2002).

##### b. Motor control

Striatal SST_2_ receptors are involved in control of extrapyramidal motor systems, as activation of SST_2_ receptors in rats increases locomotor activity (Marazioti et al., 2008; Santis et al., 2009), whereas disruption of the SST_2_ receptor gene in mice impairs motor functions (Viollet et al., 2000; Allen et al., 2003).

##### c. Feeding and drinking

In accordance with the widespread distribution of SST_2_ in the hypothalamus, the receptor also plays a role in drinking and feeding behavior. Activation of SST_2_ increases food intake by suppressing satiety (i.e., a mechanism delaying onset of another meal after a completed one), but not satiation (a mechanism causing meal termination) (Stengel et al., 2015). Increased meal numbers mediated by SST_2_ activation likely involve lateral hypothalamic orexinergic-A neurons projecting to the arcuate neuropeptide Y neurons that express orexin receptors 1. SST_2_ actions on orexinergic neurons, however, seem to be indirect (Stengel et al., 2015). Activation of SST_2_ also increases rapid-onset water consumption (Karasawa et al., 2014). This dipsogenic function involves activation of the angiotensin II receptor type 1 signaling system. SRIF release in the hypothalamus follows a circadian rhythm, with the highest release at the beginning of the dark phase in rats. Early nocturnal drinking and feeding in rats are therefore physiologically regulated by SST_2_ signaling (Stengel et al., 2015).

##### d. Stress response

Stress responses are inhibited by SRIF, and SST_2_ plays a major role in inhibition of acute stress induced at several levels. First, stress-related endocrine responses are inhibited by SST_2_ at both the CNS and pituitary levels (Prevot et al., 2017; Stengel and Taché, 2017). Second, stress-related sympathetic activation is inhibited by SST_2_ at the level of brainstem presympathetic neurons (Burke et al., 2008). Finally, behavioral stress responses, such as suppression of food intake and anxiety, are also inhibited by SST_2_ (Stengel et al., 2015; Stengel and Taché, 2017). In addition to anxiolytic effects, SST_2_ also mediates antidepressant actions of SRIF (Engin et al., 2008; Engin and Treit, 2009; Fee et al., 2017; Prevot et al., 2017).

#### 3. Retina

In the retina, SST_2_ inhibits adenylyl cyclase, K^+^/Ca^2+^ conductances, as well as activates guanylyl cyclase and NOS, and plays an important role in positive control of dopamine and negative control of glutamate release (Cervia et al., 2008). SST_2_ signaling through these diverse intracellular pathways converges into an important retinal neuroprotection (Casini et al., 2005; Vasilaki and Thermos, 2009). Therefore, SRIF administration is a promising therapeutic approach in treating retinal diseases involving ischemia and excitotoxicity, and a multicentric, phase II–III, randomized, controlled clinical trial (EUROCONDOR-278040) is underway to assess the efficacy of SRIF administration in diabetic retinopathy (Hernandez and Simo, 2013; Hernandez et al., 2014; Simo and Hernandez, 2014).

### H. Somatostatin Receptor 2 Ligands

**Fig. 10.**
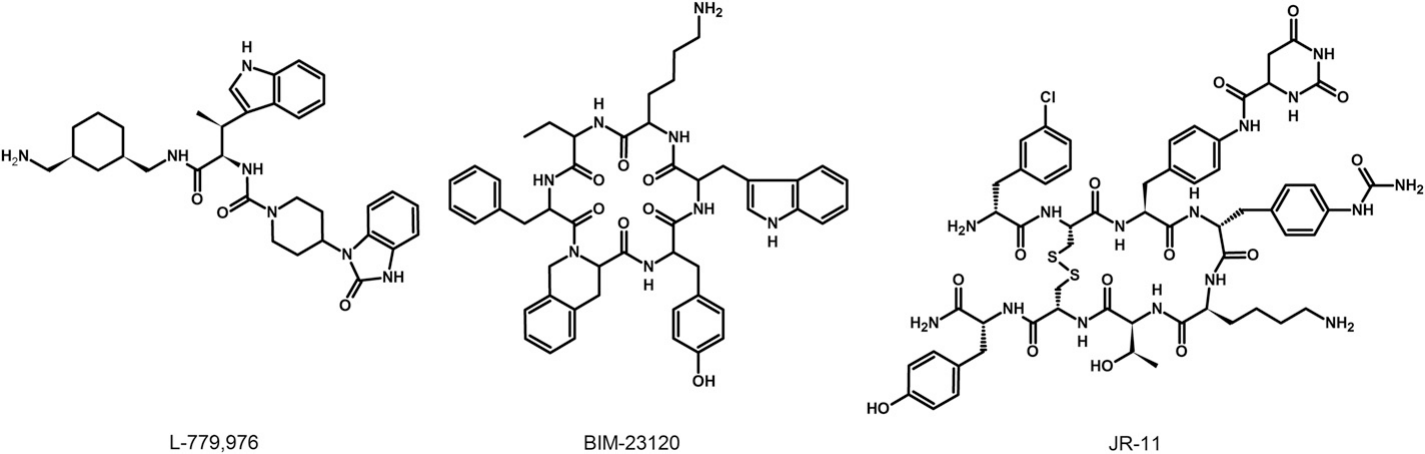
Structures of synthetic SST_2_ ligands. L-779,976 and BIM-23120, SST_2_ agonists; JR-11, SST_2_ antagonist.

Early efforts to develop stable SRIF analogs with potent inhibitory activity on GH release have led to the synthesis of SST_2_-preferring peptide ligands, two of which, octreotide and lanreotide, have later been approved for clinical use. Both exhibit potent (subnanomolar) full agonistic properties at the SST_2_ and modest activity (low nanomolar) at SST_5_. In clinical practice, however, octreotide and lanreotide fully normalize GH and IGF-1 in only ∼50% of unselected acromegalic patients (Carmichael et al., 2014), which has stimulated the search for new SRIF analogs. This has led to recent development of pasireotide, which exhibits affinity for multiple SSTs. Pasireotide is particularly potent at the SST_5_, but binds with modest affinity to SST_2_, where it exhibits only partial agonistic activity (Bruns et al., 2002; Lesche et al., 2009; Poll et al., 2010). BIM-23120 is a highly selective SST_2_ peptide agonist often used to study SST_2_ activity (Gruszka et al., 2012; Günther et al., 2016). L-779,976 was the first selective nonpeptide agonist (Fig. 10; Table 5) (Rohrer et al., 1998) with high SST_2_ selectivity. However, further development of L-779,976 was halted because of low oral bioavailability. Abundant SST_2_ expression in human tumors has stimulated a continued search for orally available SST_2_-selective agonists such as L-054,264, RFE-007, as well as novel *β*-methyltryptophan derivatives (Yang et al., 1998; Palii et al., 2008; Banno et al., 2017). Several peptide antagonists of SST_2_ have also been identified, such as BIM23627 and BIM-23454. At high concentrations these compounds exhibit some residual agonist activity and are thus weak partial agonists (Pöll et al., 2010). Among available SST_2_ peptide antagonists, JR11 is currently the most potent and selective one (Fig. 10; Table 5) (Cescato et al., 2008).

**TABLE 5:** Ligand-binding affinities of SST_2_-selective ligands

	SST_1_	SST_2_	SST_3_	SST_4_	SST_5_
L-779/976*^a^*	2760	0.05	729	310	4260
BIM-23120*^b^*	>1000	0.34	412	>1000	213.5
DOTA-JR11*^c^*	>1000	0.58	>1000	>1000	>1000

^a^ Data from Rohrer et al. (1998).

^b^ Data from Gruszka et al. (2012).

^c^ Data from Cescato et al. (2008).

## VI. Somatostatin Receptor 3

### A. Somatostatin Receptor 3 Structure

**Fig. 11.**
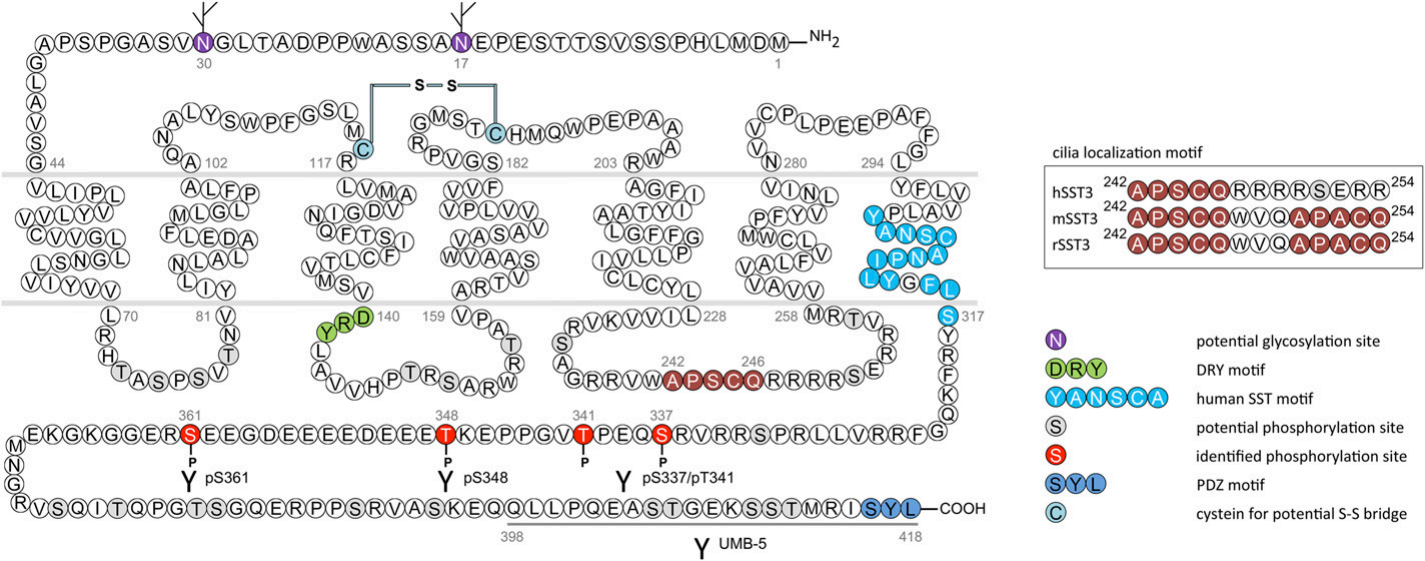
Structure of human SST_3_. The primary and secondary amino acid structure of the human SST_3_ (UniProtKB - P32745) is shown in a schematic serpentine format. Glycosylation sites are colored in purple; the DRY motif is highlighted in green; the human SST motif is in light blue; potential phosphorylation sites are in gray; identified GRK2/3 phosphorylation sites are in red; the PDZ ligand motif is in dark blue; the cilia localization motif is in dark red; and the disulfide-forming cysteines are in pale blue. UMB-5 is a rabbit monoclonal antibody, which detects the carboxyl-terminal tail of SST_3_ in a phosphorylation-independent manner.

The mouse SST_3_ was cloned as the third member of the SST family (Yasuda et al., 1992), and cloning of the human SST_3_ was reported shortly thereafter (Yamada et al., 1992b). The gene encoding for human SST_3_ is localized on chromosome 22q13.1 and spreads over eight exons. However, the entire coding region is localized in a single exon and encodes a protein of 418 amino acids (Fig. 11). The SST_3_ protein sequence shares 46% homology with SST_2_. Analysis of the sequence showed two potential *N*-glycosylation sites located in the amino-terminal domain at Asn^17^ and Asn^30^. The genes encoding mouse and rat SST_3_ are localized on chromosome 15 E1 and 7q34, respectively; both encode a protein of 428 amino acids. In rat SST_3_, Asp^124^ is essential for binding of the endogenous peptide ligand SRIF-14 (Nehring et al., 1995). A unique feature of mouse and rat SST_3_ is selective targeting to primary neuronal cilia (Händel et al., 1999) dependent on the presence of a conserved ciliary targeting motif within the third ICL (^243^APSCQWVQAPACQ^255^). This sequence is identical in mouse and rat and contains a dual (AX[A/S]XQ) ciliary targeting motif found in GPCRs efficiently localized to cilia (Berbari et al., 2008a; Jin et al., 2010; Geneva et al., 2017). In contrast, the third ICL of the human SST_3_ contains only a single (AX[A/S]XQ) motif. Consequently, the human SST_3_ is not selectively localized to primary cilia but is predominantly observed at the plasma membrane in many cell types. The calculated mol. wt. of the nonglycosylated protein is approximately 46 kDa. However, in Western blots derived from human pituitary and transfected cells, SST_3_ is detected as a broad smear of approximately 70–80 kDa (Lupp et al., 2012). After peptide N-glycosidase F (PNGase F) treatment, the mol. wt. is reduced to approximately 48 kDa, and the receptor protein appears as a sharp band (Lupp et al., 2012), indicating that the native SST_3_ protein is indeed heavily glycosylated. SST_3_ is unique among SST subtypes in that it exhibits a very long carboxyl-terminal tail. In contrast to all other SSTs, the SST_3_ carboxyl-terminal tail lacks a potential palmitoylation site. Amino acid sequences of both the third ICL and the carboxyl-terminal tail are not conserved across species.

### B. Somatostatin Receptor 3 Signaling Mechanisms

**Fig. 12.**
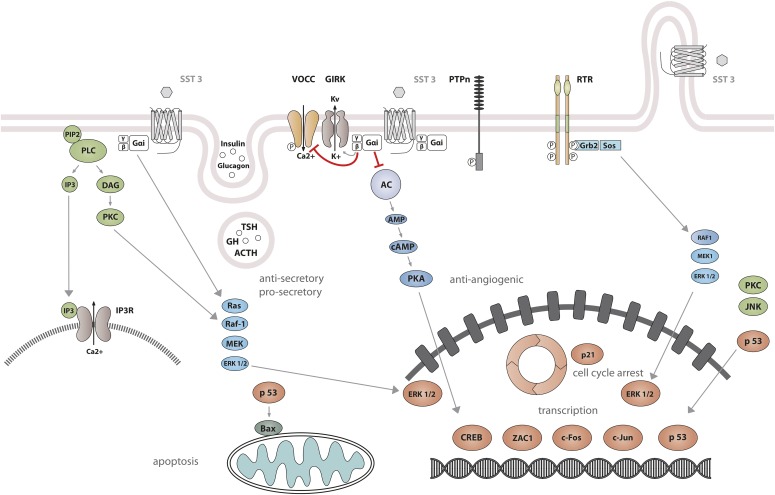
SST_3_ signaling leading to inhibition of hormone secretion, proliferation, and induction of apoptosis. By coupling to Gi proteins, SST_3_ inhibits adenylate cyclase and reduces cAMP accumulation and reduces intracellular Ca^2+^ concentrations by activating GIRK channels, which results in membrane hyperpolarization and subsequent reduction of Ca^2+^ influx through VOCC. This results in decreased hormone secretion. By coupling to a pertussis toxin–independent G protein (probably Gq), SST_3_ activates PLC, triggering inositol-1,4,5-trisphosphate (IP_3_) production and subsequent Ca^2+^ release into the cytoplasm from endoplasmic reticulum. SST_3_-dependent induction of apoptosis involves p53 and Bax.

SST_3_ is a G_i/o_-coupled receptor. Agonist activation results in increased incorporation of guanosine 5′-O-[gamma-thio]triphosphate (GTP*γ*S) into membranes of SST_3_-transfected cells (Lesche et al., 2009). Its major effector systems are inhibition of adenylyl cyclase (Yamada et al., 1992b; Lesche et al., 2009), activation of K_ir_3.x currents (Günther et al., 2016), and modulation of VOCCs (Fig. 12) (Mergler et al., 2008). In transfected cells, transient activation of ERK can be detected. These effects are greatly diminished when cells are preincubated with PTX, strongly indicating that G_i/o_ proteins are major effectors of SST_3_. Exogenously expressed human SST_3_ activates PLC, which is only partly blocked by PTX, suggesting involvement of G_q_ proteins. However, the physiologic relevance of PLC activity is unknown (Siehler and Hoyer, 1999c). When expressed in stable rat pituitary tumor cells (GC cells), human SST_3_ exhibits constitutive ligand-independent activity that inhibits basal cAMP/protein kinase A (PKA) signaling and suppresses GH transcription through glycogen synthase kinase 3B activation (Eigler et al., 2014). Heterologously expressed SST_3_ also induces antiproliferative or proapoptic cell-specific effects (War et al., 2015). SST_3_-induced apoptosis in CHO-K1 cells involves induction of transformation-related protein 53 (p53) and Bax (Sharma et al., 1996).

### C. Somatostatin Receptor 3 Regulation

Human SST_3_ contains a very long carboxyl-terminal tail of 102 amino acids, compared with only 66 and 56 residues in SST_2_ and SST_5_, respectively. Sequence homology within the carboxyl-terminal region of the mouse, rat, and human SST_3_ is rather low. Upon agonist exposure, human SST_3_ is phosphorylated at four carboxyl-terminal hydroxyl amino acids, namely S337, T341, T348, and S361 (Lehmann et al., 2016). Direct evidence for agonist-induced phosphorylation of these residues has been provided by generation of phosphosite-specific antibodies. For rat SST_3_, S341, S346, S351, and T357 have been identified as major phosphoacceptor sites using whole-cell phosphorylation assays (Roth et al., 1997a). Interestingly, phosphorylation sites for the human and rat SST_3_ identified to date reside within the proximal part of the SST_3_ carboxyl-terminal tail. Alignment of phosphorylation motifs identified in SST_2_ and SST_5_ suggests that agonist-mediated phosphorylation of these receptors occurs preferentially at a defined distance from the NPXXY motif, which marks the end of the seventh transmembrane region and the beginning of the carboxyl-terminal tail. This observation is supported by functional analysis of receptor mutants. Exchange of S/T sites to A within the proximal part of the carboxyl-terminal tail greatly diminished *β*-arrestin recruitment and SST_3_ internalization (Lehmann et al., 2016). In contrast, mutation of S/T residues within the distal part of the carboxyl-terminal tail had no effect on receptor trafficking. SRIF-induced phosphorylation is completely blocked by the SST_3_-selective antagonist NVP-ACQ090 (Roth et al., 1997a; Lehmann et al., 2016). Phosphorylation occurs rapidly within seconds to minutes, whereas SST_3_ dephosphorylation occurs more slowly. In transfected HEK293 cells, agonist-induced phosphorylation is primarily mediated by GRK_2_ and GRK_3_ (Lehmann et al., 2016). The four identified phosphorylation sites do not undergo protein kinase C- or PKA-mediated phosphorylation. Dephosphorylation of SST_3_ specifically requires PP1*α* and PP1*β* (Lehmann et al., 2016).

Another unique feature of the SST_3_ is its rapid downregulation upon prolonged agonist exposure. This effect has been clearly documented for both human and rat SST_3_ in transfected cells (Tulipano et al., 2004; Lesche et al., 2009; Lehmann et al., 2016). Downregulation of about 50% of cellular SST_3_ protein is observed between 6 and 12 hours of continued agonist exposure. Loss of human and rat SST_3_ occurred similarly with SRIF, octreotide, or pasireotide as ligands. In contrast, no such downregulation was observed with human SST_2_ or SST_5_ under similar conditions (Tulipano et al., 2004; Lesche et al., 2009). It is not known, however, whether endogenous SST_3_ undergoes such rapid downregulation. Such studies are difficult to perform because human cell lines expressing sufficient levels of endogenous SST_3_ allowing immunochemical detection of receptor protein are not available. Degradation of SST_3_ was blocked by the lysosomal inhibitor chloroquine and the cell-permeable proteasome inhibitor MG132 (Tulipano et al., 2004). For rat SST_3_, agonist-induced ubiquitination occurs at intracellular lysine residues. Mutation of all intracellular lysine residues to arginine in a K-R mutant of the human SST_3_ prevents downregulation only during the first 6 hours of treatment. After 24 hours, downregulation of the K-R mutant occurs to the same extent as for wild-type SST_3_, suggesting that ubiquitination of SST_3_ facilitates, but is not an absolute requirement for degradation (Lehmann et al., 2016).

### D. Somatostatin Receptor 3 Trafficking

SST_3_ is regulated like a prototypical GPCR in that it is internalized within minutes upon exposure to the endogenous SRIF-14 ligand. The time course and extent of receptor internalization are similar to that observed for SST_2_. Internalization is preceded by recruitment of *β*-arrestins to the activated SST_3_ (Kreuzer et al., 2001). In fact, *β*-arrestin-2 is more efficiently recruited than *β*-arrestin-1. Interestingly, SRIF-14 bound to internalized SST_3_ is rapidly degraded, whereas octreotide is recycled as an intact peptide (Roosterman et al., 2008). Phosphorylation is a precondition for arrestin translocation and internalization. SST_3_ and arrestin form transient complexes (Lehmann et al., 2016). Although SST_3_ receptors are internalized via clathrin-coated pits into early endosomes, arrestins dissociate from the receptor and redistribute into the cytosol (Tulipano et al., 2004; Lehmann et al., 2016). This is in contrast to SST_2_, which forms stable complexes with arrestins that cointernalize into early endosomes. After internalization, only a proportion of SST_3_ recycles back to the plasma membrane. Remaining intracellular receptors are transferred to larger diameter vesicles (presumably lysosomes) for degradation (Tulipano et al., 2004). This action is also in contrast to SST_2_, which completely recycles to the plasma membrane within 60–90 minutes after agonist removal. Differential endosomal sorting of SST_3_ and SST_2_ has been demonstrated using an immunocytochemical pulse–chase assay that estimates the degree to which internalized receptors remain associated with transferrin-containing endocytic vesicles. Transferrin is a well-established marker of early and recycling endosomes that mediate rapid recycling. After a 30-minute SRIF-14/transferrin pulse, a high degree of colocalization of both SST_3_ and SST_2_ with transferrin was observed. However, after an additional 20-minute pulse, only SST_2_—but not SST_3_—showed a high degree of colocalization with endocytosed transferrin, suggesting that SST_3_ are predominantly sorted to a population of endocytic vesicles distinct from those that constitute the conserved recycling pathway marked by transferrin (Tulipano et al., 2004). Ras-related in brain (Rab) proteins are markers for specific populations of endosomes. For many GPCRs, Rab proteins are major regulators of endosomal trafficking. Using real-time imaging, it was demonstrated that SST_3_ traffics through Rab4-, Rab11-, and Rab21-containing endosomes. Expression of inactive variants of these specific Rab proteins inhibits passage of SST_3_ through different endosomal compartments (Tower-Gilchrist et al., 2011).

### E. Somatostatin Receptor 3 Targeting

When expressed in HEK293 cells, mouse, rat, and human SST_3_ demonstrate bona fide plasma membrane localization. In contrast, SST_1_ is predominantly localized to intracellular vesicular compartments. In fact, transplantation of the rat SST_3_ N-terminal domain to the rat SST_1_ is sufficient to facilitate plasma membrane localization of SST_1_, suggesting that the N-terminal domain of SST_3_ contains a plasma membrane targeting sequence (Ammon et al., 2002). However, when mouse SST_3_ is expressed exogenously in polarized inner-medullary–collecting duct cells or cultured hippocampal neurons, the receptor protein is concentrated in primary cilia (Berbari et al., 2008a). Primary cilia are nonmotile plasma membrane appendages that serve specialized sensory functions, such as light sensation in photoreceptors or detection of odors in olfactory neurons. Cilia are enriched in signaling proteins, such as G proteins, adenylyl cyclases, ion channels, and arrestins. Their function is defined by the presence of specific signaling receptors. Importantly, disruption of ciliary function has been associated with human ciliopathies, such as Bardet–Biedl syndrome (BBS), Joubert syndrome, and Meckel syndrome, which have prominent functional and structural CNS phenotypes (Berbari et al., 2008b; Jin et al., 2010). In fact, SST_3_ was the first signaling receptor identified in primary neuronal cilia in mouse and rat brain (Händel et al., 1999) and is therefore a prototypical ciliary GPCR. Thereafter, additional GPCRs with selective targeting to primary cilia were identified, such as 5-hydroxytryptamine receptor 6 and melanin-concentrating hormone receptor 1 (Ammon et al., 2002). In rodents, SST_3_ was also identified in primary cilia in pancreatic islets and adenohypophysis (Iwanaga et al., 2011). Expression of SST_3_ in neuronal cilia in rodents appears after birth when first cilia are formed and persists throughout the aged brain (Stanić et al., 2009; Guadiana et al., 2016). However, selective cilia targeting appears not to be conserved across many species. The dual (AX[A/S]XQ) ciliary targeting motif in the third ICL (^243^APSCQWVQAPACQ^255^) is identical in rat and mouse. This motif is not found in any other SST subtype, and human SST_3_ contains only a single ciliary targeting motif (^242^APSCQRRRRSERR^254^). This may explain why subcellular localization of the human SST_3_ is not restricted to primary cilia in many cell types.

### F. Somatostatin Receptor 3 Interacting Proteins

The rodent SST_3_ is a prototypical ciliary GPCR. As such, SST_3_ has been intensively used to identify proteins that traffic cargo to cilia. The ciliary targeting signal of rodent SST_3_ is directly recognized by BBS proteins that form the BBSome complex required for targeting of membrane proteins to cilia (Jin et al., 2010). The BBSome is an octameric complex consisting of seven highly conserved BBS proteins, BBS1, BBS2, BBS4, BBS5, BBS7, BBS8, and BBS9, plus BBIP10. BBS is an autosomal recessive disorder characterized by retinal degeneration, polydactyly, kidney cysts, and obesity, caused by mutations in any of 14 known genes and whose etiology is associated with cilium dysfunction. In the absence of BBSome function, SST_3_ accumulates at the plasma membrane (Jin et al., 2010). In the naturally occurring *tubby* mutant mouse, which develops retinitis pigmentosa, hearing loss, and obesity, SST_3_ also fails to localize to cilia. Although it is not known whether the *tubby* protein product binds directly to SST_3_, it appears to be an accessory factor in ciliary GPCR trafficking (Sun et al., 2012). Once embedded into the ciliary plasma membrane, SST_3_ behaves as a functional GPCR. Activation by SRIF facilitates translocation of *β*-arrestin-2 into cilia. *β*-Arrestin-2 recruitment depends on SST_3_ phosphorylation and is required for removal of activated SST_3_ receptors from the ciliary space (Green et al., 2015). Interestingly, when the receptor fails to undergo BBSome- or arrestin-mediated retrieval from the cilia back into the cell, SST_3_ concentrates into membranous buds at the tips of cilia before release into extracellular vesicles via exocytosis (Nager et al., 2017). For the human SST_3_, the carboxyl-terminal domain was shown to interact with the multiple PDZ-domain protein 1 (MUPP1). MUPP1 is a tight junction scaffold protein in epithelial cells, and, as a result of the interaction with MUPP1, SST_3_ is targeted to tight junctions in human keratinocytes. Interaction with MUPP1 enables the receptor to regulate transepithelial permeability in a PTX-sensitive manner, suggesting that human SST_3_ can activate G proteins locally at tight junctions (Liew et al., 2009). Both rat and human SST_3_ form constitutive homodimers (Pfeiffer et al., 2001; Lehmann et al., 2016). When coexpressed, rat SST_2_ and SST_3_ can form heterodimers with reduced SST_3_ activity (Pfeiffer et al., 2001). However, to what extent SST_3_ can form dimers or oligomers with other GPCRs in vivo is not known.

### G. Somatostatin Receptor 3 Anatomic Framework

**Fig. 13.**
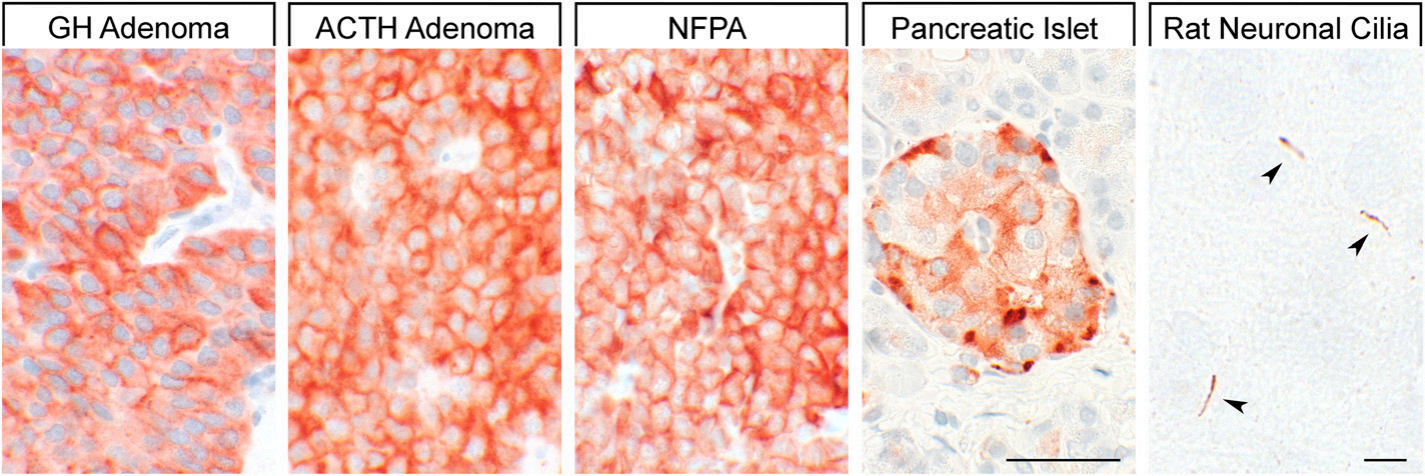
SST_3_ expression pattern in human pituitary adenomas, human pancreatic islets, and rat neuronal cilia. Immunohistochemistry (red-brown color), counterstaining with hematoxylin; primary antibody: UMB-5; scale bar, 50 *µ*m. SST_3_ displays both membranous and cytoplasmic expression. NFPA, clinically nonfunctioning pituitary adenoma.

Cellular and subcellular localizations of human SST_3_ have been studied in detail using rabbit mAb UMB-5, which is directed against the distal part of its carboxyl-terminal tail (^398^QLLPQEASTGEKSSTMRISYL^418^) (Fig. 13). In normal human tissues, SST_3_ is present at the plasma membrane of distinct cell populations in the anterior pituitary, pancreatic islets, enteric ganglion cells of the GIT, the zona fasciculata and zona reticularis of the adrenal cortex, as well as the adrenal medulla, glomeruli and tubules of the kidney, luteinized granulosa cells of the ovary, and immune cells (Lupp et al., 2012). Interestingly, nonfunctioning pituitary adenomas (mostly of gonadotroph lineage) express high levels of SST_3_, whereas expression of SST_2_ and SST_5_ is very low or absent (Lupp et al., 2012; Lee et al., 2015). In most ACTH-producing adenomas, SST_3_ and SST_5_—but not SST_2_—are present. In contrast, rodent SST_3_ receptors are selectively targeted to primary cilia in pancreatic islets and anterior pituitary and to primary neuronal cilia in many brain regions, including cerebral cortex, hippocampus, hypothalamus, and amygdala (Händel et al., 1999; Iwanaga et al., 2011). Another brain region with high SST_3_ mRNA expression is the cerebellum, where SST_3_ is expressed on Bergmann glial cells (Händel et al., 1999).

SST_3_ is also present in different human tumor types. It is consistently observed with high intensity of expression, especially in pituitary adenomas. In GH-producing pituitary adenomas, noticeable amounts of SST_5_ and SST_3_ are also expressed, besides high levels of SST_2_ (Lupp et al., 2012; Casar-Borota et al., 2013), whereas in most of ACTH-producing adenomas only SST_5_ and SST_3_, but not SST_2_, were detected (Lupp et al., 2011, 2012). In contrast, in gonadotropic and nonfunctioning pituitary tumors, high levels of SST_3_ were observed, whereas SST_2_ and SST_5_ expression was low or even absent (Lupp et al., 2012; Gabalec et al., 2015; Lee et al., 2015). SST_3_ is expressed in about 30%–50% of pheochromocytomas and paragangliomas (Elston et al., 2015). In addition, SST_3_ was found most prominently expressed in thymomas (Ferone et al., 2000). Although SST_2_ is clearly the most prominent receptor expressed in gastroenteropancreatic neuroendocrine neoplasms, SST_3_ was also detected in 52%–90% of cases (Lupp et al., 2012; Kaemmerer et al., 2015b; Qian et al., 2016). However, and in contrast to SST_2_, strong SST_3_ expression was only noted in 5%–29% of cases (Kaemmerer et al., 2015b; Qian et al., 2016; Song et al., 2016). Low SST_3_ expression levels occur also in bronchopulmonary neuroendocrine neoplasms (Kaemmerer et al., 2015a). Likewise, in about 50% of tumors with neuroendocrine differentiation derived from breast, cervix, or prostate, weak to moderate SST_3_ expression was observed (Mizutani et al., 2012). SST_3_ was also detected in 56% of GIST tumors (Zhao et al., 2014).

### H. Somatostatin Receptor 3 Function

At the cellular level, activation of SST_3_ inhibits hormone release. Inhibition of GH release has been observed in GC cells (Eigler et al., 2014). SST_3_ agonists inhibit insulin release from INS-1 cells (Mergler et al., 2008). At the systemic level, highly selective SST_3_ antagonists such as MK-1421 or MK-4256 facilitate glucose-stimulated insulin secretion from pancreatic *β*-cells and block glucose excursion in wild-type, but not SST_3_ KO mice (Pasternak et al., 2012; Shah et al., 2015; He et al., 2016). This suggests that SST_3_ antagonism represents a new potential mechanism for treating type 2 diabetes mellitus. SST_3_ expressed in primary neuronal cilia in rodent brain is critical for object recognition memory. SST_3_ KO mice are severely impaired in discriminating novel objects, whereas they retain normal memory for object location. Similarly, systemic injection of the SST_3_ antagonist NVP-ACQ090 disrupts recall of familiar objects in wild-type mice (Einstein et al., 2010). In addition, the anticonvulsant effects of CST-14 in rodents can be blocked by the selective SST_3_ antagonist SST_3_-ODN-8, suggesting that this activity is mediated in part via the SST_3_ (Aourz et al., 2014).

### I. Somatostatin Receptor 3 Ligands

**Fig. 14.**
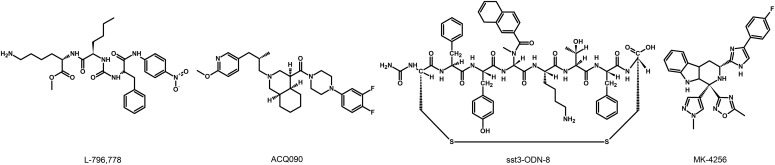
Structures of synthetic SST_3_ ligands. L-796,776, SST_3_ agonist; ACQ090, sst_3_-ODN-8, and MK-4256, SST_3_ antagonists.

SRIF-14 is a full agonist at the SST_3_, mediating strong G protein signaling, full phosphorylation, and internalization of the receptor (Fig. 14) (Lehmann et al., 2016). Compared with SRIF-14, octreotide and pasireotide behave as full agonists with regard to G protein signaling and as partial agonists with regard to receptor phosphorylation and internalization (Lehmann et al., 2016). L-796,778 was the first selective nonpeptide SST_3_ agonist with a moderate affinity (Rohrer et al., 1998); however, compared with SRIF-14, it behaves as a weak partial agonist on G protein signaling and does not induce noticeable receptor phosphorylation or internalization (Fig. 14; Table 6) (Lehmann et al., 2016). The first selective SST_3_ antagonist discovered, SST_3_-ODN-8, was successfully used to label endogenous SST_3_ in human tissues using autoradiographic binding studies (Fig. 14; Table 6) (Reubi et al., 2000a). Great progress has been made in development of two structurally distinct classes of selective nonpeptide SST_3_ antagonists based on tetrahydro-*β*-carboline and decahydroisoquinoline derivatives (Poitout et al., 2001; Troxler et al., 2010; He et al., 2016). The decahydroisoquinoline derivative ACQ090 is a full neutral antagonist that blocks phosphorylation and internalization of SST_3_ completely (Fig. 14; Table 6) (Lehmann et al., 2016). Tetrahydro-*β*-carboline derivatives such as MK-1421 or MK-4256 are highly selective for SST_3_. They were shown to facilitate glucose-stimulated insulin secretion from pancreatic *β*-cells and block glucose excursion in rodents in vivo (Pasternak et al., 2012; Shah et al., 2015; He et al., 2016). MK-4256 has been evaluated as a potential candidate for treatment of type 2 diabetes mellitus (Fig. 14; Table 6) (He et al., 2012, 2016; Shah et al., 2015). However, development was discontinued due to adverse cardiovascular effects related to human ether-a-go-go–related gene off-target side effects (He et al., 2014). Attempts to eliminate this off-target action have led to discovery of (4-phenyl-1*H*-imidazol-2-yl)-methanamines as potent and selective SST_3_ agonists (Li et al., 2014; Lai et al., 2015).

**TABLE 6:** Ligand-binding affinities of SST_3_-selective ligands

	SST_1_	SST_2_	SST_3_	SST_4_	SST_5_
L-796/778*^a^*	1255	>10,000	24	8650	1200
sst_3_-ODN-8*^b^*	>10,000	>10,000	4.1	>10,000	>10,000
ACQ090*^c^*	5.68	5.31	8.13	6.81	5.93
MK-4256*^d^*	2362	4025	0.66	384	533

^a^ Data from Rohrer et al. (1998).

^b^ Data from Reubi et al. (2000a).

^c^ Data from Troxler et al. (2010).

^d^ Data from He et al. (2012).

## VII. Somatostatin Receptor 4

### A. Somatostatin Receptor 4 Structure

**Fig. 15.**
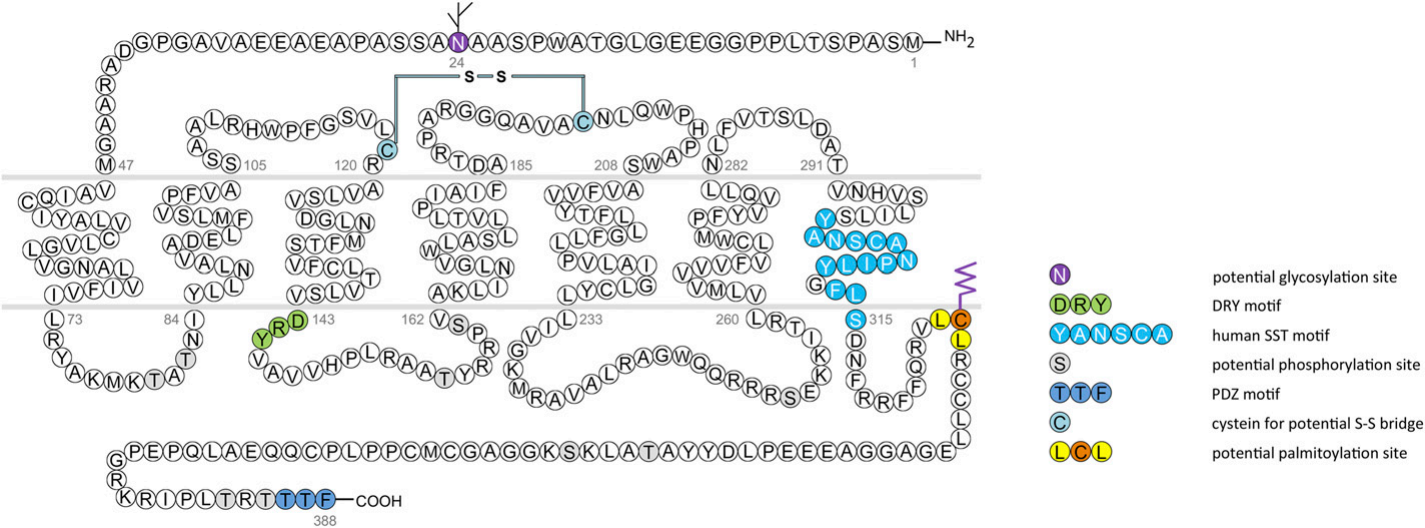
Structure of human SST_4_. The primary and secondary amino acid structure of the human SST_4_ (UniProtKB - P31391) is shown in a schematic serpentine format. The glycosylation site is colored in purple; the DRY motif is highlighted in green; the human SST motif is in light blue; potential phosphorylation sites are in gray; the PDZ ligand motif is in dark blue; the disulfide-forming cysteines are in pale blue; and the potential palmitoylation site is in orange.

cDNAs coding for rat and human SST_4_ were cloned in 1992 and 1993, respectively, and the rat tissue distribution of the mRNA suggested a brain-specific receptor subtype (Bruno et al., 1992; Demchyshyn et al., 1993; Rohrer et al., 1993). The gene encoding human SST_4_ is localized on chromosome 20p11.2 in a single exon. Genes encoding mouse and rat SST_4_ are localized on chromosomes 2 G3 and 3q41, respectively. Human SST_4_ and rat SST_4_ are proteins of 388 and 384 amino acids, respectively, and show 88% sequence identity. Both human and rodent SST_4_ feature one site for N-linked glycosylation in the N-terminal domain and a putative palmitoylation motif in the C-terminal tail. The calculated mol. wt. is approximately 42 kDa. However, in Western blots derived from rat brain, SST_4_ protein is detected as a broad smear of 60–70 kDa (Schreff et al., 2000). After PNGase F digestion, the mol. wt. is reduced to approximately 45 kDa and the receptor protein appears as a sharp band (Schreff et al., 2000), indicating that the native SST_4_ is indeed glycosylated. SST_4_ shares a conserved sequence (^351^YANSCANPILY^361^) (Fig. 15) in transmembrane region 7 (mammalian SST signature) and a consensus motif (X-[S/T]-X-Φ) in its C terminus with all the other mammalian SST subtypes (UniProtKB accession: P31391). The X-S/T-X-Φ motif (the hydrophobic amino acid Φ is phenylalanine in SST_4_) is regarded as a potential PDZ domain binding site and might be important for interaction with scaffolding proteins (Christenn et al., 2007). Crystal structure of the agonist-bound SST_4_ is not yet available, and studies on ligand binding and receptor activation have been sparse. A homology model of SST_4_ has been generated using the active state *β*_2_-adrenergic receptor crystal structure, and a number of reported ligands have been docked to the model-built structure. This molecular prediction analysis suggested two partially overlapping binding modes (Liu et al., 2012).

### B. Somatostatin Receptor 4 Signaling Mechanisms

**Fig. 16.**
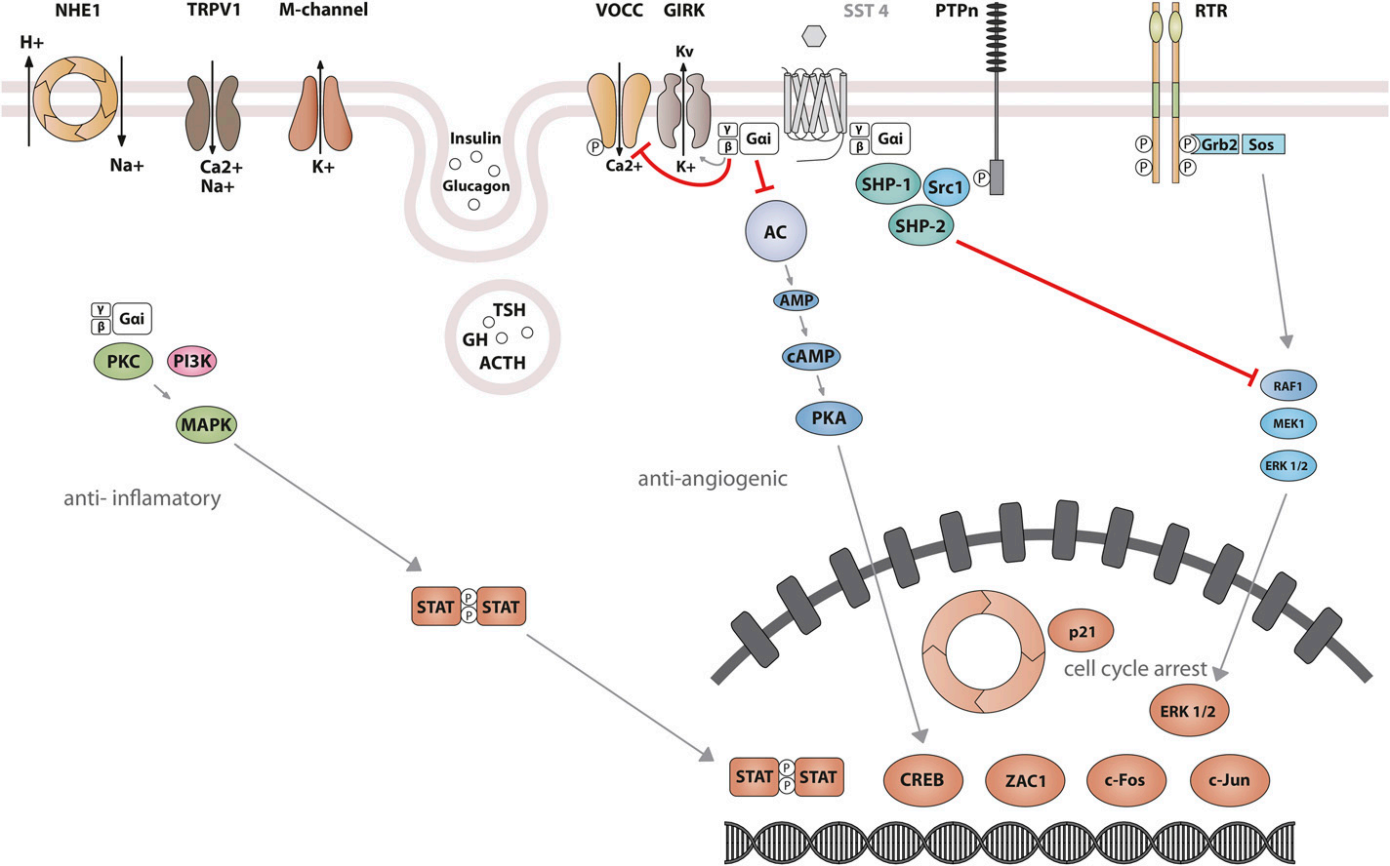
SST_4_ signaling leading to inhibition of hormone secretion, proliferation, and migration. By coupling to Gi proteins, SST_4_ inhibits adenylate cyclase and reduces cAMP accumulation, and reduces intracellular Ca^2+^ concentrations by activating GIRK and M channels, which results in membrane hyperpolarization and subsequent reduction of Ca^2+^ and Na^+^ influx through VOCC and TRPV1. In addition, SST_4_ inhibits the NHE1 activity, resulting in a decrease of extracellular acidification rate. Another major effector of SST_4_ is the tyrosine phosphatase SHP-2, which mediates antiproliferative effects. SST_4_ also mediates a prolonged ERK activation and subsequent signal transducer and activator of transcription 3 phosphorylation, which is Gi/Go and PI3K dependent. Activation of SST_4_ can induce cell cycle arrest by upregulation of the cyclin-dependent kinase inhibitor p21 (cip1/WAF1).

SST_4_ is coupled to heterotrimeric G_i/o_ protein–mediated adenylate cyclase inhibition (Fig. 16) (Demchyshyn et al., 1993; Patel et al., 1994). In cortical neurons, SST_4_ has been linked to activation of M currents (Qiu et al., 2008). Native SST_4_ modulates K_ir_3.x, VOCC, and transient receptor potential cation channel subfamily V member 1 in rat DRG neurons (Gorham et al., 2014b; Schuelert et al., 2015). In rat retinal ganglion cells, SST_4_ also modulates VOCC (Farrell et al., 2010, 2014). In transfected cells, agonist-stimulated SST_4_ activates phospholipase A2, leading to production of arachidonic acid as a second messenger, the MAPK signaling cascade (Bito et al., 1994; Smalley et al., 1999), and a NHE1 (Smalley et al., 1998). In CHO cells expressing human recombinant SST_4_, SRIF stimulates basal proliferation through a mechanism involving prolonged activation of mitogen-activated protein kinases 1/2 (ERK1/2) and phosphorylation of signal transducer and activator of transcription 3 (STAT3) (Sellers et al., 1999). This event is transduced by G_i/o_ proteins and is dependent on PI3K activity. However, SST_4_ exerts more complex functions in regulating cell proliferation. For example, SST_4_ causes prolonged activation of p38 MAPK, which in turn results in the induction of the cell cycle inhibitor p21 (cip1/Waf1) and growth arrest, when cells were simultaneously exposed to somatostatin and basic FGF (Alderton et al., 2001). When expressed in malignant pleural mesothelioma cells, human SST_4_ activates SHP-1 and SHP-2. Downstream signaling through SHP-2 is required for cytostatic effects of SST_4_ observed in these cells (Yamamoto et al., 2014). In transfected CHO cells, SST_4_ causes PI3K-dependent activation of NHE1 and increases extracellular acidification rate (Smalley et al., 1999). In contrast, SST_4_ inhibits the ubiquitous NHE1 in transfected rat fibroblasts (Lin et al., 2003).

### C. Somatostatin Receptor 4 Regulation

The 5′ promoter region of the human gene (Petersenn et al., 2002b) bears little homology between the human and the rat 5′ flanking regions (Xu et al., 1995b). A minimal −209-bp 5′ flanking region contains elements that support human promoter activity in different cell types in vitro, including rat pituitary cells. Elements located between −459 and −984 bp enhance promoter activity, although putative binding sites for tissue-specific transcription factors were not identified in these regions. Pathophysiological factors affecting SRIF and SST_1-5_ expression in endothelial cells include hypoxia, which induces SST_4_ mRNA expression in human umbilical vein endothelial cells (Dal Monte et al., 2011). In brain endothelial cells, proinflammatory cytokines and lipopolysaccharide upregulate expression of SST_4_ as well as SST_2_ (Basivireddy et al., 2013). Substantial differences between rat and the human SST_4_ have been reported in desensitization after prolonged SRIF treatment. In transfected CHO cells expressing human SST_4_, both activation of NHE1 and stimulation of ERK phosphorylation were susceptible to a marked desensitization in response to SRIF (Smalley et al., 1998, 1999; Engström et al., 2006).

### D. Somatostatin Receptor 4 Trafficking

As to the fate of the receptor upon SRIF-14 binding, low levels of internalized human SST_4_ were detected in transfected cells. The use of radiolabeled ligand suggested rapid dissociation of the complex and rapid recycling of the ligand to the extracellular medium and of the receptor to the plasma membrane, respectively. These observations may suggest that sustained desensitization of human SST_4_ is not entirely dependent on receptor sequestration (Smalley et al., 2001). In contrast, rat SST_4_ is not susceptible to rapid desensitization and does not undergo internalization at all, as shown in transfected cells and in brain tissue after in vivo treatment of rats with SRIF (Kreienkamp et al., 1998; Schreff et al., 2000). Although a number of potential phosphate acceptor sites are present, rat SST_4_ is also not subject to agonist-induced phosphorylation and does not recruit *β*-arrestins to the plasma membrane when activated (Tulipano et al., 2004). Site-directed mutagenesis allowed for identification of a single amino acid residue (Thr^331^) in rat SST_4_, which confers resistance to agonist-induced internalization (Kreienkamp et al., 1998).

### E. Somatostatin Receptor 4 Interacting Proteins

Direct interaction between SST_4_ and the scaffolding PSD-95 has been shown in transfected HEK293 cells and in hippocampal neurons (Christenn et al., 2007). This interaction is mediated by binding of a PDZ domain of PSD-95 to a PDZ-domain ligand motif in the C-terminal tail of SST_4_. PSD-95 is not involved in regulating receptor signaling in transfected cells. Moreover, as PSD-95 and SST_4_ partially colocalize in hippocampal neurons in the dendritic domain, the scaffold protein may be involved in targeting SST_4_, mainly localized in the somatodendritic postsynaptic domain in brain. In addition, an interaction between SST_4_ and the membrane glycoprotein dipeptidyl peptidase-4/cluster of differentiation 26 (CD26) occurs in malignant pleural mesothelioma cells. CD26 is a 110-kDa type II membrane glycoprotein with known dipeptidyl peptidase IV activity in its extracellular domain. SST_4_ and CD26 are highly coexpressed and interact through their cytoplasmic domains in malignant pleural mesothelioma cells. The SST_4_–CD26 interaction reduces cytostatic effects of SST_4_ agonists (Yamamoto et al., 2014). Their efficacy was enhanced by suppression of CD26 as well as by treatment of cells with anti-CD26 mAbs. Upon treatment with anti-CD26 mAbs, SST_4_ aggregated preferentially in lipid rafts. Interestingly, SHP-2 also clustered in lipid rafts along with SST_4_, which in turn facilitated SST_4_-mediated cytostatic and antitumor effects. Moreover, in transfected HEK293 cells, human SST_4_ exists as constitutive homodimers and as constitutive heterodimers when coexpressed with human SST_5_ (Somvanshi et al., 2009). However, in a different cellular background (CHO-K1 cells), cotransfection of these two receptors did not result in heterodimerization under otherwise identical conditions (Rocheville et al., 2000b). Nevertheless, to what extent the SST_4_–SST_5_ interaction may be physiologically relevant is not known.

### F. Somatostatin Receptor 4 Anatomic Framework

The SST_4_ is localized in diverse rat brain areas, and there is substantial consistency between mRNA and SST_4_-like immunoreactivity distribution in the rat (Fehlmann et al., 2000; Schreff et al., 2000; Schulz et al., 2000a; Stumm et al., 2004). High levels of SST_4_ are present throughout the rat forebrain, whereas the signal progressively decreased toward caudal brain regions. SST_4_ is abundantly expressed in the olfactory bulb and in other olfactory structures. SST_4_ is found throughout layers I–VI of the neocortex, in the hippocampus formation, the hilar region of the dentate gyrus, the amygdala, and the hypothalamus. SST_4_ immunoreactivity is distributed along neuronal processes in the striatum, nucleus accumbens, and globus pallidus. In addition, approximately 50% of DRG showed SST_4_-like immunoreactivity (Bär et al., 2004). SST_4_ is also abundantly present in retinal ganglion cells (Farrell et al., 2010). At the cellular and subcellular level of adult rat CNS, SST_4_ is preferentially distributed to somatodendritic neuronal domains. In the neocortex, hippocampus, and striatum, SST_4_ is almost exclusively confined to dendrites and symmetric synapses. In the hippocampus, targeting to asymmetric, excitatory synapses was observed. Colocalization studies of SRIF and SST_4_ provided evidence of close apposition of SRIF-containing axons and their terminals with dendrites containing SST_4_, suggesting that SST_4_ mainly functions postsynaptically (Schreff et al., 2000). In the periphery, SST_4_ is expressed in the lung, heart, and placenta and is undetectable in pancreatic islets (Fehlmann et al., 2000; Ludvigsen et al., 2015). Given that highly specific rabbit mAbs are not yet available, the cellular expression of human SST_4_ is less well characterized. Using polyclonal antibodies, SST_4_ receptors have been found in cells of the bronchial glands, the exocrine pancreas, in the GIT (stomach and duodenum), in kidney tubules, and in the parathyroid gland (Taniyama et al., 2005). Lack of SST_4_ binding sites and immunoreactivity in hypophyseal tissue suggests that SST_4_ does not play a major role in SRIF-mediated neuroendocrine control of the human anterior pituitary (Panetta and Patel, 1995; Reubi et al., 2001). SST_4_ immunoreactivity in human brain was restricted to the gray matter in cerebral cortex areas. In the sensory and motor cortex, staining of the large motor neurons was not detected. Immunopositive pyramidal cells were found in cortical layers III–VI, in agreement with results of SST_4_-like immunoreactivity in rat neocortex layers III–V, most likely representing targets of local SRIFergic neurons in the cerebral cortex. SST_4_ protein was found in the hippocampal formation, with immunostaining of cell bodies and processes in the polymorphic layer of the dentate gyrus and in the thalamus, where it localized particularly in fibers. SST_4_ immunoreactivity was also observed in the cerebellar cortex and the medulla (Selmer et al., 2000). In summary, data on SST_4_ distribution in selected human brain areas correlate well with the distribution in rat brain. Comparison between SST_4_ immunoreactivity and SST_4_ mRNA distribution by in situ hybridization (Schindler et al., 1995; Thoss et al., 1996b; Piwko et al., 1997) in human brain showed some discrepancies in thalamus, cerebral cortex, and cerebellum expression patterns (Selmer et al., 2000). According to receptor autoradiography using selective ligands, SST_4_ cannot be frequently found in human tumors (Reubi et al., 2001). Finally, two studies reported divergent results on the expression of SST_4_ in human insulinomas. In the first study, immunoreactivity analysis suggested that SST_4_ was the most frequent receptor expressed in both benign and malignant insulinomas (Portela-Gomes et al., 2007). In the second study, the mRNA expression analysis and binding assays suggested that SST_4_ expression was limited to approximately 20% of tumors (Bertherat et al., 2003).

### G. Somatostatin Receptor 4 Function

SST_4_ is expressed in areas involved in learning and memory processes. In mice, pharmacological studies suggest that activation of hippocampal SST_4_ leads to impaired spatial learning and enhanced cued memory. This effect suggests a switch from formation of hippocampus-based memory to striatum-based memory (Gastambide et al., 2010). In addition, behavioral studies using selective ligands showed that activation of SST_4_ in the striatum increases rat locomotor activity via glutamatergic systems (Raynor et al., 1993a; Santis et al., 2009). Finally, activation of SST_4_ in the CNS plays a role in modulation of behavioral responses to acute stress and of behavioral and neuroendocrine changes induced by mild chronic stress in mice, suggesting involvement of SST_4_ in anxiety and depression-like behavior (Scheich et al., 2016, 2017). Experimental data suggest SST_4_ as a therapeutic target in Alzheimer’s disease. Administration of the SST_4_ agonist NNC26-9100 was found to reduce soluble amyloid-*β* peptide oligomers in the brain by enhancing metalloproteinase-mediated amyloid degradation in two different mouse models. This effect correlated with improved learning (Sandoval et al., 2012, 2013). Interestingly, SST_4_ expression levels are drastically reduced in the temporal cortex of female Alzheimer’s disease patients (Gahete et al., 2010b). SRIF is also highly expressed in brain areas associated with seizures. Activation of SST_4_ suppressed epileptiform activity in mouse hippocampal slices and exerts anticonvulsant effects in vivo. Moreover, SST_4_ KO mice showed increased susceptibility to limbic seizures (Qiu et al., 2008) but other studies suggested excitatory and proconvulsant effects of SST_4_ activation (Moneta et al., 2002). In a rat model for limbic seizures, intrahippocampal administration of a SST_4_-selective agonist has marked anticonvulsant effects, similar to administration of SST_2_ and SST_3_ agonists (Aourz et al., 2011). SST_4_ is currently being evaluated as a therapeutic target for development of anti-inflammatory and/or analgesic drugs without endocrine side effects (Sándor et al., 2006; Elekes et al., 2008; Helyes et al., 2009; Schuelert et al., 2015). Mice lacking SST_4_ exhibit increased inflammatory and nociceptive responses, suggesting impaired defense mechanisms (Helyes et al., 2009; Van Op den Bosch et al., 2009). Peripheral administration of selective SST_4_ agonists reduced formalin-induced acute nociception and mechanical allodynia in arthritic and neuropathic pain models and exhibited multiple anti-inflammatory effects in rodents (Sándor et al., 2006; Schuelert et al., 2015). DRG neurons are most likely a primary target of SST_4_ agonists. SST_4_ activation reduces membrane excitability in DRG neurons by activating K_ir_3.x and inhibiting VOCC channels, and both mechanisms are presumed to contribute to analgesic effects (Gorham et al., 2014a). SST_4_-selective agonists reduced acute and chronic airway inflammation as well as bronchial hyper-reactivity in the mouse, and inhibited carbachol-induced bronchoconstriction (Elekes et al., 2008).

### H. Somatostatin Receptor 4 Ligands

**Fig. 17.**
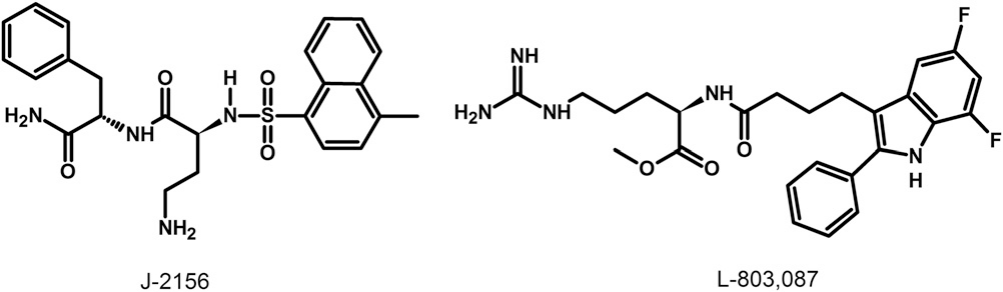
Structures of synthetic SST_4_ ligands. J-2156 and L-803,087, SST_4_ agonists.

Compared with other SST subtypes, SST_4_ displays somewhat lower affinity for the common endogenous ligand SRIF-14. Among multi-SST ligands, the cyclic heptapeptide veldoreotide (COR005) (previously called somatoprim, DG3173) is unique in that it binds to SST_4_ in addition to SST_2_ and SST_5_ (Shimon et al., 2004; Plöckinger et al., 2012). Veldoreotide is a potent suppressor of GH secretion from human pituitary adenomas, which is attributed to its affinity for SST_2_ and SST_5_. Interestingly, despite nanomolar affinity for SST_5_, veldoreotide has minimal effects on insulin secretion from endocrine pancreas in vivo. In phase II clinical trials for the treatment of acromegaly, veldoreotide proved to be effective when administered by s.c. bolus or s.c. infusion (ClinicalTrials.gov NCT02235987 and NCT02217800). TT-232 is a stable cyclic heptapeptide with partial activity at SST_4_ that also binds to SST_1_ (Crider and Witt, 2007). Compound J-2156, a nonpeptide agonist displaying high selectivity and high affinity for SST_4_ (Fig. 17; Table 7), was derived by solid-phase synthesis of a series of 1-naphthalenesulfonylamino-peptidomimetics (Engström et al., 2005). Both TT-232 and J-2156 exhibited anti-inflammatory and antinociceptive effects after i.p. administration in rodents (Crider and Witt, 2007). NNC26-9100 is the lead compound of another structurally distinct class of highly selective SST_4_ agonists (Liu et al., 1998). L-803,087 is a nonpeptidic agonist with high affinity and selectivity for SST_4_ (Fig. 17; Table 7) (Rohrer et al., 1998). Unexpectedly for peptide GPCRs, only SST_4_ agonists have been identified, and antagonists are not yet available. SST_4_ agonists able to penetrate the blood brain barrier would be of great interest.

**TABLE 7:** Ligand-binding affinities of SST_4_-selective ligands

	SST_1_	SST_2_	SST_3_	SST_4_	SST_5_
L-803,087*^a^*	199	4720	1280	0.7	3880
J-2156*^b^*	350	>5000	1300	0.8	460

^a^ Data from Rohrer et al. (1998).

^b^ Data from Engström et al. (2005).

## VIII. Somatostatin Receptor 5

### A. Somatostatin Receptor 5 Structure

**Fig. 18.**
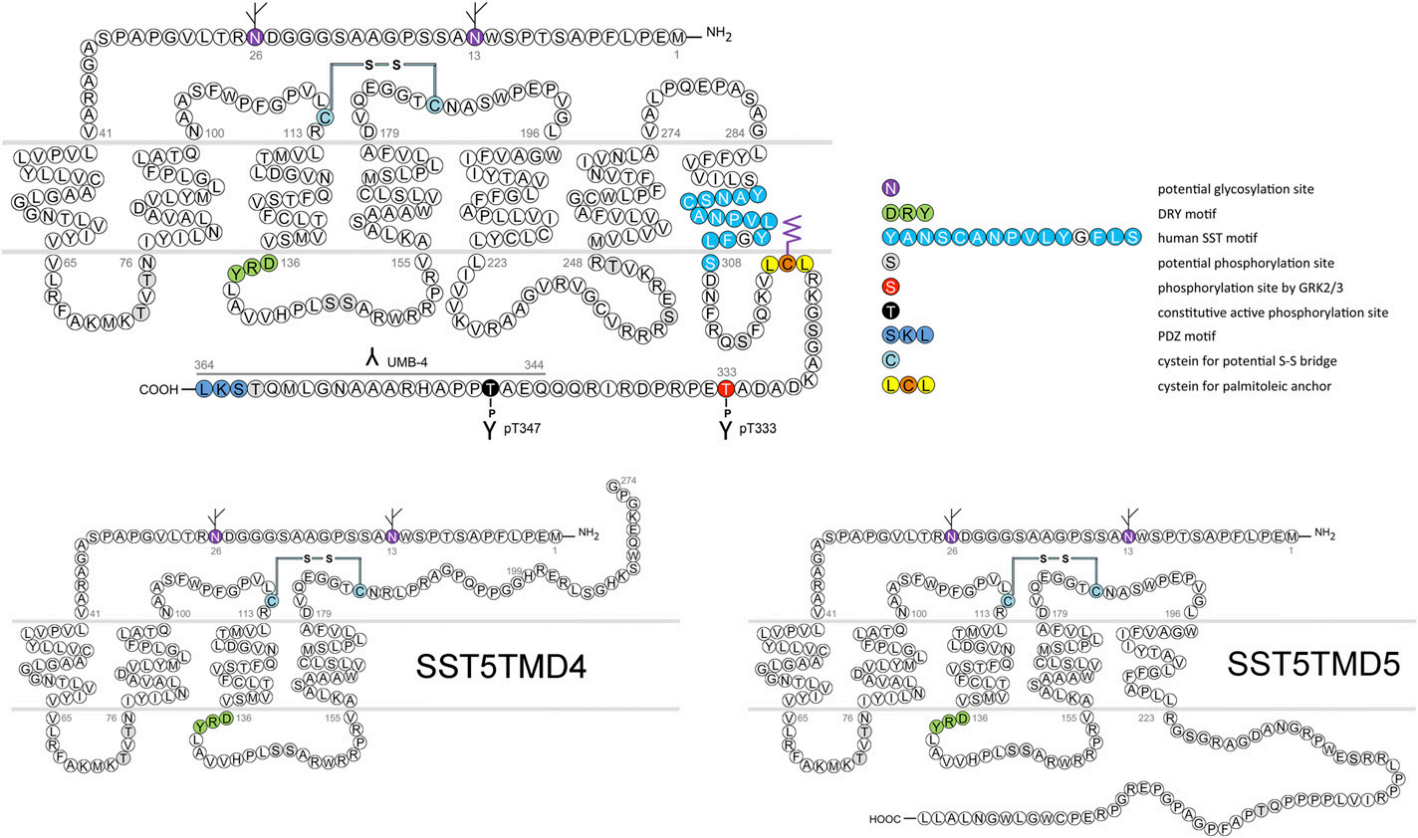
Structure of human SST_5_. The primary and secondary amino acid structure of the human SST_5_ (UniProtKB - P35346) as well as its truncated variants SST_5_TMD4 and SST_5_TMD5 are shown in a schematic serpentine format. Glycosylation sites are colored in purple; the DRY motif is highlighted in green; the human SST motif is in light blue; potential phosphorylation sites are in gray; identified GRK2/3 phosphorylation site is in red; constitutive phosphorylation site is in black; the PDZ ligand motif is in dark blue; the disulfide-forming cysteines are in pale blue; and the potential palmitoylation site is in orange. UMB-4 is a rabbit monoclonal antibody, which detects the carboxyl-terminal tail of SST_5_ in a phosphorylation-independent manner.

Cloning of human SST_5_, the last subtype of the SST family to be cloned (Yamada et al., 1993; Panetta et al., 1994), was preceded by that of rat SST_5_ (O’Carroll et al., 1992), which was initially termed rat SSTR4, due to the temporal proximity with the cloning of the receptor currently known as rat SST_4_ (Xu et al., 1993). After an initial period of confusion, the current nomenclature was agreed upon (Hoyer et al., 1995a), and subsequent studies led to detailed characterization of SST_5_. The human *SSTR5* gene is localized on chromosome 16p13.3, and its coding sequence spans a single exon, encoding a protein of 364 amino acids (Panetta et al., 1994; Takeda et al., 1995), whereas the rat *Sstr5* receptor gene encodes a protein of 363 amino acids (O’Carroll et al., 1992). Genes encoding mouse and rat SST_5_ are localized on chromosomes 17 A3.3 and 10q12, respectively. Mouse SST_5_ was also shown to encode a protein whose length appears to vary from 362 amino acids (Moldovan et al., 1998; Gordon et al., 1999) to 363 residues (Lublin et al., 1997; Feuerbach et al., 2000), and up to 385 amino acids (Baumeister et al., 1998), differences attributed to cloning procedures or mouse strain. Initial comparative analysis had revealed that the sequence of SST_5_ is evolutionarily well conserved, with human SST_5_ showing 80% homology with the amino acid sequence of rat and mouse SST_5_ (O’Carroll et al., 1992; Lublin et al., 1997). Nevertheless, compared with other SSTs, human SST_5_ seems to display lower levels of identity and similarity with SST_5_ from other species, particularly at the carboxyl-terminal tail, as well as with other human SSTs [ranging from 42% to 52% compared with SST_1_, SST_2_, SST_3_, and SST_4_ (O’Carroll et al., 1992; Panetta et al., 1994; Møller et al., 2003)], which portrays SST_5_ as the least conserved subtype among SSTs. Original cloning revealed two potential *N-*glycosylation sites in human SST_5_, located at Asn-13 and Asn-26 in the amino-terminal segment, and a third Asn-187 in the second ICL (Fig. 18) (Yamada et al., 1993; Panetta et al., 1994). Likewise, the 385-amino-acid mouse SST_5_ described by Baumeister et al. (1998) contains three equivalent putative *N-*glycosylation sites at residues 36, 46, and 208. The estimated molecular mass of the 364-residue human SST_5_ is 39 kDa, whereas immunodetection of SST_5_ from transfected baby hamster kidney cells in Western blot showed several bands ranging from 52 to 66 kDa. Deglycosylation with PNGase F rendered a single band with an estimated 40-kDa mass, thus confirming the glycoprotein nature of the receptor (Helboe et al., 1997). Human SST_5_ contains a cysteine residue at position 320 as a presumed palmitoylation anchor, the mammalian SST signature motif in transmembrane region 7 (^294^YANSCANPVLY^304^), and two cysteine residues at residues 112 in the first extracellular loop (ECL) and 186 in the second ECL, predicted to enable a disulfide bridge (Reisine and Bell, 1995). Studies on structural determinants of human SST_5_ function using mutational analysis suggested that ECL 2 is key to form the receptor ligand-binding pocket (Greenwood et al., 1997).

In contrast to other SSTs, a number of studies have identified single-nucleotide polymorphisms in the human SST_5_ gene that may imply potential pathophysiological functions in pancreatic NETs and other cancers (Li et al., 2011; Zhou et al., 2011, 2012), bipolar affective disorder (Nyegaard et al., 2002), acromegaly (Lania et al., 2008; Ciganoka et al., 2011), prostate cancer (Hormaechea-Agulla et al., 2017), and in the regulation of circulating IGF-1 and IGFBP3 in prostate and breast cancer (Gu et al., 2010). However, only a single SST_5_ mutation associated with acromegaly has been described in a single patient (Ballare et al., 2001).

The human *SSTR5* gene as well as rodent orthologs uniquely undergoes noncanonical splicing to truncated variants that possess less than the typical seven TMDs that characterize all GPCRs (Cordoba-Chacon et al., 2011). In particular, two truncated human SST_5_ receptors exist, termed SST_5_TMD4 and SST_5_TMD5, which display distinct tissue distribution, subcellular localization, response to ligands, and signaling capacities as compared with canonical full-length SST_5_ (Cordoba-Chacon et al., 2011). The most studied variant, SST_5_TMD4, is scarcely present in normal tissues but abundantly expressed in a number of tumors, including pituitary adenomas (Durán-Prado et al., 2009, 2010; Gatto et al., 2013a; Luque et al., 2015), breast cancer (Durán-Prado et al., 2012b; Gahete et al., 2016), thyroid cancer (Puig-Domingo et al., 2014), medullary thyroid carcinoma (Mole et al., 2015), NETs (Sampedro-Nunez et al., 2016), and prostate cancer (Hormaechea-Agulla et al., 2017). In all those tumor types, SST_5_TMD4 expression is associated with features of enhanced tumor aggressiveness that vary depending on the type of tumor: increased cell survival/proliferation, migration, invasion, angiogenesis, decreased apoptosis, poor response to octreotide/lanreotide, etc. Similar, albeit not identical, truncated SST_5_ receptor variants have also been cloned and characterized in pig (Durán-Prado et al., 2012a) and rodents (Cordoba-Chacon et al., 2010). Truncated SST_5_ receptor variants share a number of features, as follows: 1) preferential intracellular distribution (rather than the predominant plasma membrane localization of full-length SST_5_); 2) functional capacity to selectively respond to ligands (e.g., SRIF for SST_5_TMD5; CST for SST_5_TMD4), by modulating distinct signaling pathways (cAMP, Ca^2+^, etc.); and 3) ability to physically interact with full-length SST_2_ and/or SST_5_, to retain them in intracellular compartments, and, eventually, disrupt their normal function. As a result, it has been proposed that SST_5_TMD4 and other truncated variants may act as functional dominant-negative partners for their respective full-length SST_2_ and SST_5_ receptor counterparts (Cordoba-Chacon et al., 2011).

### B. Somatostatin Receptor 5 Signaling Mechanisms

**Fig. 19.**
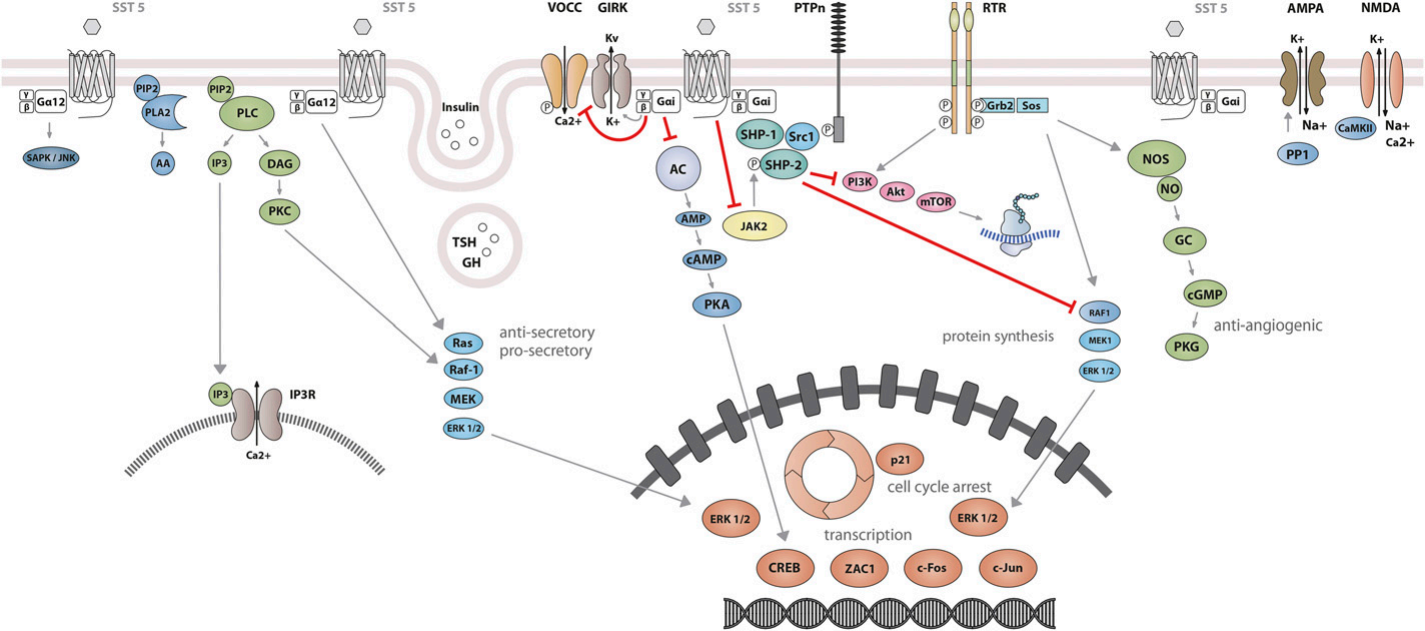
SST_5_ signaling leading to inhibition of hormone secretion and proliferation. By coupling to Gi proteins, SST_5_ inhibits adenylate cyclase and reduces cAMP accumulation, and reduces intracellular Ca^2+^ concentrations by activating GIRK channels, which results in membrane hyperpolarization and subsequent reduction of Ca^2+^ influx through VOCC. This results in decreased hormone secretion. By coupling to a pertussis toxin–independent G protein, SST_5_ activates PLC, triggering inositol-1,4,5-trisphosphate (IP_3_) production and subsequent Ca^2+^ release into the cytoplasm from endoplasmic reticulum. Major downstream effectors of SST_5_ are the tyrosine phosphatases SHP-1 and SHP-2, which subsequently inhibit mTOR pathway, thereby decreasing cell growth and proliferation. In addition, SST_5_ inhibits NHE1 activity, resulting in a decrease of extracellular acidification rate.

SST_5_ signals through a wide array of mechanisms, which include prototypical G*_α_*_i_-dependent inhibition of adenylyl cyclase common to all SSTs, regulation of other crucial enzymes like PTPs and MAPK, as well as modulation of free cytosolic calcium and potassium concentrations (Fig. 19) (Møller et al., 2003; Peverelli et al., 2009, 2013; van der Hoek et al., 2010; Theodoropoulou and Stalla, 2013). Original cloning studies showed that both human and rat SST_5_ inhibited forskolin-stimulated cAMP accumulation through a G*_α_*_i_ protein–dependent mechanism (Yamada et al., 1993; Panetta et al., 1994). These functions were confirmed in subsequent studies, mainly using SST_5_-transfected cells and/or selective ligands, which revealed that SST_5_ activates additional pathways in a context (i.e., ligand, cell environment)- and species-dependent manner (Siehler and Hoyer, 1999b,c; Møller et al., 2003; van der Hoek et al., 2010; Theodoropoulou and Stalla, 2013). Thus, SST_5_ activates PLC activity, thereby increasing cytosolic Ca^2+^ levels by release from intracellular stores (Wilkinson et al., 1997a,b; Siehler and Hoyer, 1999c). However, SST_5_ also blocks VOCCs, thereby decreasing Ca^2+^ cell entry and cytosolic levels (Tallent et al., 1996), likely by hyperpolarizing the cell through K^+^ influx via activation of K_ir_3.x (Kreienkamp et al., 1997; Smith et al., 2001). Using wild-type and mutant SST_5_, the main signaling pathways activated by this receptor have been delineated, as follows: 1) SST_5_ couples to individual G*_α_*_i1–3_ and G*_α_*_oA,B_; 2) G*_α_*_o_ mediates antisecretory and antimitogenic effects of SST_5_ in human pituitary somatotrophs; 3) the DRY motif is crucial for SST_5_ coupling with downstream effectors, whereas the BBXXB motif within the third ICL is dispensable for cAMP inhibition but essential for SST_5_ actions to reduce intracellular calcium levels and inhibit ERK1/2 activation, as well as for *β*-arrestin/receptor interaction and receptor internalization; and 4) residues 328–347 within the C terminus may play an inhibitory role in receptor internalization (Ballare et al., 2001; Peverelli et al., 2008, 2009, 2013). In fact, earlier mutational analysis indicated that Cys320 and, by and large, the C-tail of SST_5_ are essential for functional effector coupling (e.g., to adenylyl cyclase) and agonist-induced receptor desensitization and internalization (Hukovic et al., 1998).

SST_5_ inhibits ERK1/2 by a dephosphorylation cascade, including inhibition of guanylate cyclase and inhibition of cGMP-dependent protein kinase G (Cordelier et al., 1997). It also exerts antiproliferative effects by activating neuronal NOS via p60src kinase (Cordelier et al., 2006). SST_5_ activates stress-activated protein kinase or c-Jun N-terminal kinase via G*α*_12_ proteins (Komatsuzaki et al., 2001). In addition, it activates and upregulates N-methyl-D-aspartate receptor function by a mechanism involving calmodulin-dependent kinase II, PLC, protein kinase C, and tyrosine kinases in hippocampal noradrenergic nerve endings (Pittaluga et al., 2005). In rat retinal ganglion cells, SST_5_ mediates suppression of 2-amino-3-(5-methyl-3-oxo-2,3-dihydro-1,2-oxazol-4-yl)propanoic acid (AMPA) responses by acting through a signaling cascade involving G_i/o_/cAMP-PKA/ryanodine/Ca^2+^/CAM/calcineurin/PP1 (Deng et al., 2016).

SST_5_ displays constitutive activity, resulting in tonic inhibition of cAMP and ERK1/2 signaling and thus reducing hormone secretion (Ben-Shlomo et al., 2007, 2009b; Ben-Shlomo and Melmed, 2010). Finally, it has been shown that very low concentrations of SRIF actually stimulate GH release from pig and nonhuman primate somatotrophs mediated via SST_5_ and involving adenylyl cyclase, cAMP/PKA, and intracellular Ca^2+^ pathways (Luque et al., 2006; Cordoba-Chacon et al., 2012b). These actions may also be related to the presence of truncated SST_5_ receptor variants, which activate a wide array of signaling routes and molecular effectors, in a cell-, species-, and tumor-dependent manner, including cAMP, intracellular Ca^2+^, mitogen-activated protein kinases (ERK/c-Jun N-terminal kinase), AKT, cyclin D3, actin-related protein 2/3, MYC/myc-associated factor X, Wingless/int-1, and retinoblastoma protein signaling components (Durán-Prado et al., 2009, 2012a,b; Cordoba-Chacon et al., 2010; Hormaechea-Agulla et al., 2017).

### C. Somatostatin Receptor 5 Regulation

#### 1. Regulation of Somatostatin Receptor 5 Gene Expression

The mouse *Sstr5* gene contains two introns in the 5′-flanking region, which would enable the potential use of alternative gene promoters (Gordon et al., 1999; Baumeister and Meyerhof, 2000a; Olias et al., 2004). In humans, cell-specific expression was initially assigned to the first 900 bp of the *SSTR5* gene (Greenwood et al., 1994; Baumeister and Meyerhof, 2000a), but a subsequent study identified a 6.1-kb intron in the 5′-UTR that unveiled a new upstream promoter, which can drive tissue-specific activation of the gene in pituitary in a Pit1-independent manner, but not in other tissues (e.g., small intestine, lung, or placenta) (Petersenn et al., 2002a). Similar to the mouse *Sstr5* gene promoter, the human *SSTR5* promoter lacks consensus sites for TATA or CAAT boxes, YY1, or a comparable initiator sequence, but contains relevant regulatory elements, including an essential GC-rich region containing SP1 binding sites, located proximal upstream of the transcription start site, two thyroid hormone response elements (between −1741 and −1269 and −317 and −101), and a cAMP-responsive element (between −101 and transcription start site). Indeed, reporter assays confirmed that forskolin and thyroid hormones enhance and SRIF inhibits promoter activity, which was not altered by other treatments, including IGF-1, estrogens, glucocorticoids, and phorbol 12-myristate 13-acetate (Greenwood et al., 1994; Petersenn et al., 2002a). Additional putative binding sites were identified for basic and tissue-specific transcription factors [e.g., nuclear factor 1, SP1, octamer-binding transcription factor 1, AP-1, AP-2, pituitary-specific positive transcription factor 1, Krox, pancreas-specific transcription factor 1, MyoD] and for hormone-dependent regulation [e.g., the cAMP-responsive element and tetracycline-responsive promoter element mentioned above, retinoic acid receptor, estrogen receptor, glucocorticoid receptor sites, etc.], as well as two CpG islands (Greenwood et al., 1994; Petersenn et al., 2002a).

Expression of the *SSTR5* receptor gene is under multifactorial regulation that includes the following: 1) homologous control by SRIF and its analogs; 2) heterologous regulation by key stimulatory hypothalamic hormones, such as GHRH and ghrelin, which commonly inhibit *SSTR5* receptor expression (Luque et al., 2004; Cordoba-Chacon et al., 2012a); and 3) endocrine–metabolic control by hormones from the major regulatory axes, such as sex steroids (17*β*-estradiol, testosterone), thyroid hormones, and glucocorticoids (reviewed in Baumeister and Meyerhof, 2000a; Olias et al., 2004; Ben-Shlomo and Melmed, 2010).

#### 2. Ligand-Dependent Regulation of Somatostatin Receptor 5

The SST_5_ carboxyl-terminal tail contains only two potential phosphorylation sites at residues T333 and T347 (compared with the seven putative phosphate–acceptor sites in SST_2_), which seem to undergo markedly divergent dynamics: whereas T347 is constitutively phosphorylated even in the absence of ligand, T333 is phosphorylated by GRK_2_ immediately after agonist binding, as shown with phosphosite-specific antibodies (Petrich et al., 2013; Schulz et al., 2014), and as supported by mutagenesis studies, which also point to T333 as an essential residue for SST_5_ receptor internalization (Peverelli et al., 2008). This latter study also suggested that the third ICL of SST_5_ is key for *β*-arrestin binding and receptor internalization upon ligand exposure, whereas the 36 terminal residues of the carboxyl-terminal tail may contribute to inhibit receptor internalization. Actually, regulation of SST_5_ by agonist-induced phosphorylation is tightly coupled to internalization and trafficking, for *β*-arrestin is recruited immediately after agonist-induced T333 phosphorylation and the receptor is subsequently internalized, in contrast to SST_2_, the SST_5_–*β*-arrestin complex is quickly disrupted, and SST_5_ traffics to early endosomes without *β*-arrestin (Petrich et al., 2013; Schulz et al., 2014). As a likely consequence of these distinct dynamics, the proportion of SST_5_ internalized after 30 minutes of SRIF exposure is considerably lower than that observed for the SST_2_ receptor (30%–40% versus 80%–90%, respectively) (Petrich et al., 2013; Schulz et al., 2014). Nevertheless, it is important to emphasize that the dynamics of SST_5_ phosphorylation (and also its trafficking) are ligand- and context-dependent. Although SRIF-14 induces rapid dose-dependent SST_5_ phosphorylation, octreotide did not cause this effect. In addition, the SST_5_-selective agonist L-817,818 or the multireceptor ligand pasireotide induced SST_5_ phosphorylation to a lesser extent than the natural ligand SRIF-14, which was only paralleled by the SST_5_-selective agonist BIM-23268 (Shimon et al., 1997b; Petrich et al., 2013; Schulz et al., 2014). Additional mechanisms influencing agonist-dependent SST_5_ phosphorylation remain to be elucidated.

The reverse process of SST_5_ dephosphorylation at T333 is driven by PP1*γ* and depends on sequences in the carboxy-terminal tail, and is more rapid for SST_5_ than for SST_2_ (Petrich et al., 2013; Lehmann et al., 2014a; Schulz et al., 2014). In contrast, mechanisms that operate constitutive phosphorylation of T347 at SST_5_ and its physiologic relevance in vivo, if any, are still to be elucidated (Schulz et al., 2014). Likewise, possible ubiquitin-mediated degradation of SST_5_ remains to be elucidated, although recent results showed a relationship between the abundance and functionality of ubiquitin-specific peptidase 8 and the degree of SST_5_ expression at both mRNA and protein levels, which could bear clinical relevance for treating corticotroph adenomas (Hayashi et al., 2016).

### D. Somatostatin Receptor 5 Trafficking

**Fig. 20.**
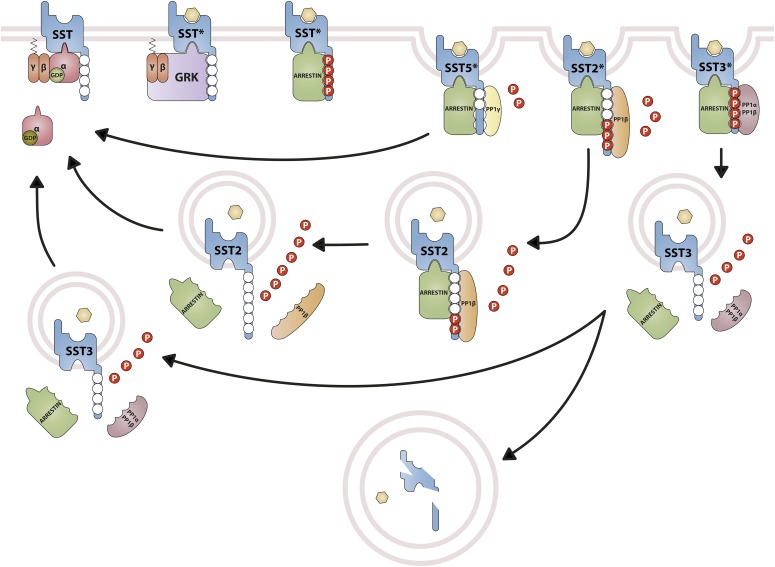
Differential trafficking of somatostatin receptors. Agonist activation of SSTs triggers activation of the associated heterotrimeric G protein that in turn stimulates a second messenger system. Quenching of this signal involves phosphorylation of the receptor by GRKs. Phosphorylation by GRKs increases the affinity for arrestins, which uncouple the receptor from the G protein and target the receptor to clathrin-coated pits for internalization. Return to its resting state requires dissociation or degradation of the agonist, dephosphorylation, and dissociation of arrestin. For SST_5_, the catalytic PP1*γ* subunit was identified to catalyze S/T dephosphorylation at the plasma membrane within seconds to minutes after agonist removal. SST_5_ forms unstable complexes with arrestins that are rapidly disrupted. After dephosphorylation, SST_5_ is either resensitized at the plasma membrane or recycled back through an endosomal pathway. For SST_2_, the catalytic PP1*β* subunit was identified to catalyze S/T dephosphorylation. SST_2_ forms stable complexes with arrestins that cointernalize into the same endocytic vesicles. This dephosphorylation process is initiated at the plasma membrane and continues along the endosomal pathway. PP1*β*-mediated dephosphorylation promotes dissociation of arrestins and, hence, facilitates quenching of arrestin-dependent signaling. Subsequently, SST_2_ is recycled back through an endosomal pathway to the plasma membrane. For SST_3_, the catalytic PP1*α*/*β* subunits were identified to catalyze S/T dephosphorylation at the plasma membrane within seconds to minutes after agonist removal. SST_3_ forms unstable complexes with arrestins that are rapidly disrupted. After dephosphorylation, SST_3_ is either subject to lysosomal degradation or recycled back to the plasma membrane through an endosomal pathway.

Trafficking of SST_5_ is intimately related to both ligand-dependent regulation of the receptor and interaction with specific proteins, which may include heterodimerization with other plasma membrane receptors. Thus, specific features of SST_5_ that reside within its sequence, phosphorylation/dephosphorylation kinetics, and interaction with *β*-arrestin and PDZ proteins are determinants for particular trafficking dynamics of this receptor. Little is known regarding the pathway of SST_5_ from its synthesis in the endoplasmic reticulum to the plasma membrane (i.e., export pathway), whereas trafficking involved in ligand-induced internalization and recycling has been studied in more detail (Fig. 20) (Jacobs and Schulz, 2008; Csaba et al., 2012; Schulz et al., 2014). Earlier studies explored the process of human and rat SST_5_ internalization and trafficking using different cell models (Hukovic et al., 1996, 1998; Roosterman et al., 1997; Roth et al., 1997b; Stroh et al., 2000b). In COS-7 cells, detailed microscopic and functional examination of SRIF-driven SST_5_ trafficking revealed that ligand exposure induced rapid (minutes) internalization of SST_5_, which involved trafficking to endosomal compartments, and was followed by a subsequent process of ligand–receptor dissociation and receptor recycling to the cell surface, accompanied by recruitment of receptors residing in an intracellular pool to the plasma membrane (Stroh et al., 2000b). Subsequent studies have dissected the contribution of specific protein regions and amino acid residues in SST_5_ trafficking, by demonstrating, for example, the importance of the third ICL (particularly phosphorylation of S242) and the carboxyl-terminal tail for agonist-induced internalization of SST_5_, which seems to be species and cell context dependent (Hukovic et al., 1998; Jacobs and Schulz, 2008; Peverelli et al., 2008; Csaba et al., 2012). In fact, SST_5_-trafficking dynamics are also heavily ligand-dependent, a feature of critical importance from a clinical standpoint. Trafficking does not solely depend on ligand-binding affinity for SST_5_, but on alternative properties that may entail, among others, distinct agonist–receptor binding sites, specific processes of phosphorylation, and subsequent activation of downstream interactions with *β*-arrestin, PDZ, and other interacting proteins (Cescato et al., 2006, 2012; Ginj et al., 2008; Jacobs and Schulz, 2008; Peverelli et al., 2008; Lesche et al., 2009; Petrich et al., 2013; Lehmann et al., 2014a; Schulz et al., 2014).

### E. Somatostatin Receptor 5 Interacting Proteins

Like the other SSTs, SST_5_ contains a potential C-terminal class I PDZ ligand motif. Human and rodent SST_5_ interact with the PDZ domain protein interacting specifically with Tc10 (PIST, a Golgi-associated protein also known as Golgi-associated PDZ and coiled-coil motif–containing protein) and with sodium/hydrogen exchanger regulatory factor (NHERF)3/PDZ-K1 (PDZ protein expressed in kidney 1), a scaffold protein (Wente et al., 2005a,b; Csaba et al., 2012; Bauch et al., 2014). Analysis of SST_5_–PIST interaction in HEK293, AtT20, and MIN6 cells suggested that PIST may accompany SST_5_ to the Golgi/TGN, and also, that it may contribute to recycling to the plasma membrane (Wente et al., 2005a,b; Csaba et al., 2012). More recent work identified additional PDZ domain proteins interacting with mouse SST_5_, such as sorting nexin family member 27 and NHERF1, and further delineated the function of PIST, which seems to retain SST_5_ at the Golgi/TGN compartment. In contrast, NHERF1 could release the receptors from this area and thereby facilitate access to the cell surface (Bauch et al., 2014). PDZ-K1/NHERF3 appears to regulate specific interaction and functional activation of PLC*β*3 by SST_5_ and other SSTs in response to SRIF by forming a ternary complex with PLC*β*3 and SSTs (Kim et al., 2012). Thus, although the PDZ motif of SST_5_ does not seem to be indispensable for agonist-induced internalization of the receptor or for recycling to the plasma membrane, it may limit lysosomal degradation (and hence increase receptor stability) and enable additional signaling capabilities through selective PDZ domain–driven interactions (Wente et al., 2005a,b; Csaba et al., 2012; Kim et al., 2012; Bauch et al., 2014).

SST_5_ displays the ability to interact with other receptors from the SST family, forming homodimers/homomers or heterodimers/heteromers. Evidence in support of the existence and functional relevance of SST_5_ homodimers as well as heteromers with SST_1_ and SST_2_ has been derived in cell models, and these have delineated some molecular determinants and mechanisms involved in these interactions (Rocheville et al., 2000b; Durán-Prado et al., 2008; Grant et al., 2008; Kumar, 2011). However, although it has been suggested that SST_2_–SST_5_ interaction could elicit relevant functional consequences in the response to SRIF analogs in acromegaly, the precise biologic and physiologic importance of these mechanisms in vivo is still a matter of debate (Grant et al., 2008). Interestingly, the truncated human SST_5_TMD4 variant disrupts normal SST_2_ homodimerization, whereas it does not interfere with homodimerization of its full-length SST_5_ counterpart. Consequently, SST_5_TMD4 only, and distinctly, reduced functional responses of SST_2_ to SRIF, but not that of canonical SST_5_, which conveys key functional consequences, as the variant may disrupt the inhibitory capacity of SST_2_ (Durán-Prado et al., 2012b). Indeed, a comparable situation occurs for truncated pig SST_5_ variants (Durán-Prado et al., 2012a). Heterodimerization of SST_5_ with a GPCR from a different family, the dopamine receptor D2 (D_2_ receptor), has also been reported (Rocheville et al., 2000a), and its potential pharmacological consequences in CNS are being explored (Szafran et al., 2012; Szafran-Pilch et al., 2017). SST_5_ formation of heteromers seems to be promiscuous as it has also shown to interact with the ghrelin receptor GHS-R1a, in a context enabling a fine, coordinated regulation of glucose-stimulated insulin secretion by SRIF and ghrelin (Park et al., 2012). In fact, SST_5_ may even interact physically and functionally with another receptor class, the tyrosine kinase receptors (Kumar, 2011).

### F. Somatostatin Receptor 5 Anatomic Framework

**Fig. 21.**
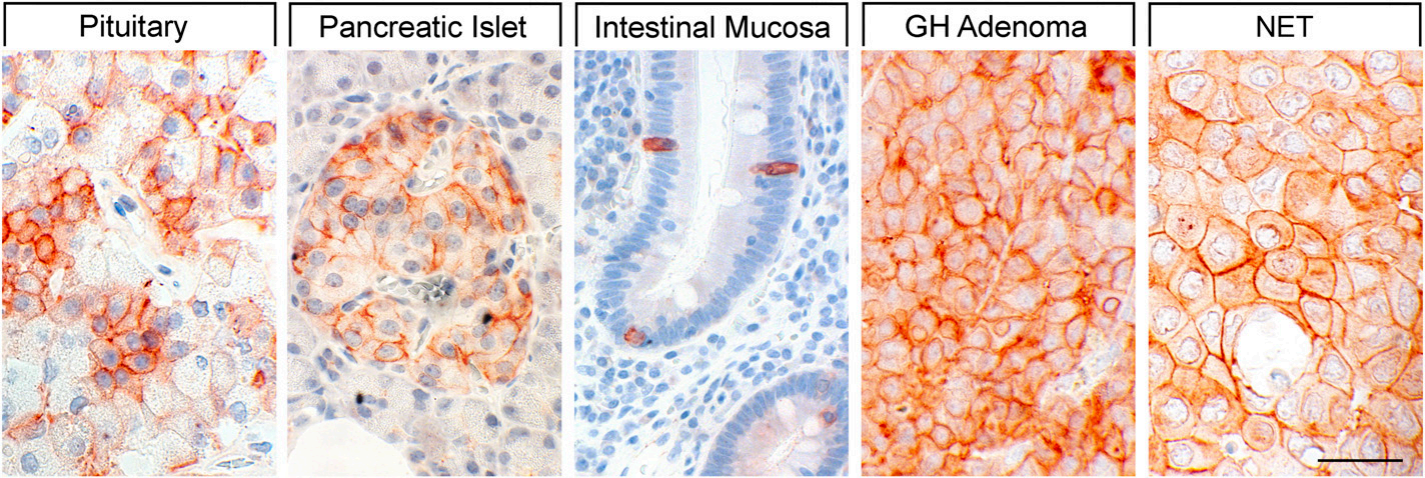
SST_5_ expression pattern in human normal and neoplastic tissues. Immunohistochemistry (red-brown color), counterstaining with hematoxylin; primary antibody: UMB-4; scale bar, 50 *µ*m. SST_5_ displays a predominant membranous expression.

By using rabbit mAb UMB-4, SST_5_ was detected both at the plasma membrane and in the cytoplasm of distinct cell populations of different normal human tissues such as GH- and ACTH-producing cells of the anterior pituitary, acinar cells, and striated ducts of the parotid glands, C cells of the thyroid, neuroendocrine- and enterochromaffin-like cells of the GI mucosa, insulin- and glucagon-secreting cells of the pancreas, cells in the reticular zone of the adrenal cortex and in adrenal medulla, glomerular endothelial cells and tubules of the kidney, luteinized granulosa cells of the ovary, luminal parts of testicular tubuli, lymphocytes in the germinal centers of lymph follicles, alveolar macrophages of the lung, singular cells scattered throughout the stroma of various organs, and single cells observed occasionally in the liver, most probably also representing macrophages (Fig. 21) (Lupp et al., 2011; Unger et al., 2012; Stollberg et al., 2016). Very limited expression of SST_5_ was detected in the brain of rodents, particularly in specific nuclei in the basal forebrain (Stroh et al., 1999).

Regarding neoplastic tissues, SST_5_ was observed at a high intensity of expression in all somatotroph and in most of corticotroph adenomas, whereas in gonadotroph and nonfunctioning pituitary adenomas SST_5_ expression was low (Fig. 21) (Lupp et al., 2011; Gabalec et al., 2015; Lee et al., 2015). Furthermore, the receptor was detected in 38%–57% of medullary and in most of (>75%) papillary and follicular thyroid carcinomas (Pazaitou-Panayiotou et al., 2012; Atkinson et al., 2013; Woelfl et al., 2014; Herac et al., 2016). To a variable extent SST_5_ was additionally detected in pheochromocytomas and paragangliomas (Lupp et al., 2011; Elston et al., 2015) and in functioning and nonfunctioning adrenocortical adenomas (Pisarek et al., 2011). Presence of SST_5_ was also noticed in lymphomas (Stollberg et al., 2016; Ruuska et al., 2018). Most notably and after SST_2_, SST_5_ represents the second most common SST subtype expressed in gastroenteropancreatic neuroendocrine neoplasms. SST_5_ was detected in 62%–93% of tumors overall (Lupp et al., 2011; Kaemmerer et al., 2015b; Qian et al., 2016; Song et al., 2016; Wang et al., 2017), with less frequent expression in pancreatic than in GI tumors, and higher expression rates in functioning than in nonfunctioning tumors (Song et al., 2016). Additionally, tumor grade correlates negatively with receptor abundance (Song et al., 2016; Wang et al., 2017), and, hence, a positive association with patient outcomes has been demonstrated for SST_5_ (Song et al., 2016; Wang et al., 2017). However, SST_5_ overexpression is also associated with vascular and nerve invasion and thus enhanced aggressiveness (Herrera-Martinez et al., 2017a). In some studies a positive correlation between SST_5_ expression and SST-based imaging was shown (Diakatou et al., 2015). Furthermore, SST_5_ was detected in 31%–45% of bronchopulmonary neoplasms (Kaemmerer et al., 2015a; Lapa et al., 2016) and occasionally also in other tumors with neuroendocrine differentiation (Mizutani et al., 2012). SST_5_ was observed in 15%–47% of GIST, and SST_2_ and/or SST_5_ immunoreactivity was associated with increased recurrence-free survival (Arne et al., 2013; Zhao et al., 2014). SST_5_ was detected in 39%–70% of colorectal cancers, and expression was higher in well to moderately differentiated tumors than in poorly differentiated ones, with a positive correlation with favorable patient outcomes (Qiu et al., 2006; Evangelou et al., 2012). SST_5_ was also detected in most breast, cervical, ovarian, and prostate carcinomas (Lupp et al., 2011), as well as in 45% of Merkel cell tumors (Gardair et al., 2015).

### G. Somatostatin Receptor 5 Function

The main physiologic functions of SST_5_ relate to control of pituitary and pancreatic endocrine secretions (Møller et al., 2003; Olias et al., 2004). SST_5_ abundance in pituitary explains its relevant role in the SRIF-mediated inhibition of GH secretion from somatotrophs, and its capacity to inhibit ACTH from corticotrophs and TSH from thyrotrophs (Kumar et al., 1997; Ren et al., 2003; Ben-Shlomo and Melmed, 2010). SST_5_ constitutive activity may also contribute to these actions (Ben-Shlomo and Melmed, 2010). Conversely, SST_5_ does not seem to participate relevantly in the physiologic control of prolactin release, although it can inhibit its secretion in prolactinomas; likewise, there is no evidence that SST_5_ contributes to regulate gonadotroph function (Møller et al., 2003; Olias et al., 2004; Ben-Shlomo and Melmed, 2010).

In the human endocrine pancreas, SST_5_ plays an important role in conveying the inhibitory actions of SRIF on glucose-stimulated insulin release, although there is also evidence for a role of SST_2_ (Zambre et al., 1999; Braun, 2014). Conversely, in rodent *β*-cells, SST_5_ is the most abundant and the predominant inhibitory receptor for glucose-induced insulin secretion, and also appears to be involved in *β*-cell development (Strowski et al., 2003; Strowski and Blake, 2008; Braun, 2014). Indeed, altered glucose and insulin regulation is the most prominent phenotype of SST_5_ KO mice, which are otherwise devoid of overt pathologic symptoms (Strowski et al., 2003; Ramirez et al., 2004; Wang et al., 2005). SST_5_ may also contribute to inhibit glucagon secretion from *α*-cells, primarily controlled by SST_2_ (Braun, 2014).

Presence and functional roles of SST_5_ in the CNS are relatively limited compared with other SSTs. In the brain, SST_5_ activation may inhibit stress-related stimulation of hypothalamic CRF and pituitary ACTH release (Stengel and Taché, 2017). SST_5_ may contribute to regulate sympathetic responses; likewise, SST_5_ may mediate gastric emptying through activation of vagal cholinergic pathways (possibly with the contribution of other receptors), as supported by its high expression in the dorsal motor nucleus of the vagus nerve (Martinez et al., 2000; Stengel et al., 2013; Stengel and Taché, 2017). Evidence for SST_5_ functions outside its endocrine and CNS actions is limited. SST_5_ is present in the rat retina, where its activation protects from AMPA–induced neurotoxicity (Kiagiadaki et al., 2010). SST_5_ is also present in cochlea, but its role and relevance are not yet known (Radojevic and Bodmer, 2014). In the reproductive tract, SST_5_ is present in Sertoli cells, where its expression is developmentally regulated (Riaz et al., 2013). In the vascular system, SST_5_ is present in smooth muscle cells of the human and mouse aorta, where it is coexpressed with truncated SST_5_TMD4 and SST_2_ and GHS-R1a to mediate protective actions of CST (Durán-Prado et al., 2013). The presence of SST_5_, either as mRNA or protein, has been described in a wide range of disorders, especially in tumors, where its precise role and potential value remain to be established (Møller et al., 2003; Barbieri et al., 2013). Of particular interest is the presence of SST_5_ in pituitary adenomas and NETs, for they already represent a valuable pharmacological target for SRIF analog treatment (van der Hoek et al., 2010; Veenstra et al., 2013).

### H. Somatostatin Receptor 5 Ligands

**Fig. 22.**
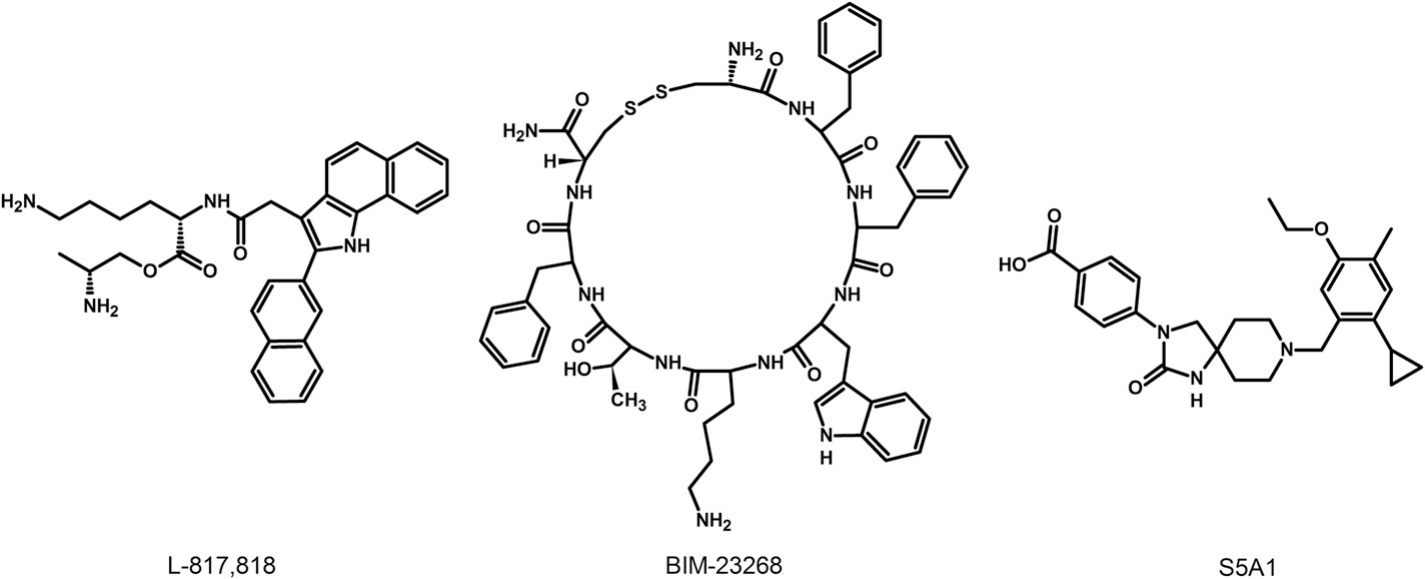
Structures of synthetic SST_5_ ligands. L-817,818 and BIM-23268, SST_5_ agonists; S5A1, SST_5_ antagonist.

Although pasireotide also exhibits affinity to SST_1_, SST_2_, and SST_3_, it binds with superior affinity to SST_5_. It also exhibits potent agonistic activity at SST_5_ and most likely mediates most of its pharmacological actions via SST_5_ (Petrich et al., 2013). The peptide agonist BIM-23268 displays moderate affinity to all SSTs (Fig. 22; Table 8); however, it appears to be unique among SST_5_ agonists in that it exhibits full agonistic activity (Shimon et al., 1997a; Petrich et al., 2013). Another peptide agonist is BIM-23206, which displays about 50-fold selectivity for SST_5_ over SST_2_ (Ren et al., 2003). L-817,818 is a moderate selective nonpeptidyl agonist (Fig. 22; Table 8) (Rohrer et al., 1998). However, it displays only partial agonistic activity at SST_5_ (Petrich et al., 2013). A series of benzoxazole piperidines was identified as high-affinity SST_5_ antagonists with virtual absence of binding to other SSTs (Martin et al., 2009). More recently, several SST_5_-selective antagonists such as S5A1 were evaluated as potential treatments for diabetes mellitus (Fig. 22). S5A1 displays a subnanomolar affinity for SST_5_ (Table 8) (Farb et al., 2017).

**TABLE 8:** Ligand-binding affinities of SST_5_-selective ligands

	SST_1_	SST_2_	SST_3_	SST_4_	SST_5_
L-817/818*^a^*	3.3	52	64	82	0.4
BIM-23268*^b^*	18.4	15.1	61.6	16.3	0.37
S5A1*^c^*	>5190	>10,000	>10,000	—	4.87

^a^ Data from Rohrer et al. (1998).

^b^ Data from Shimon et al. (1997a).

^c^ Data from Farb et al. (2017).

## IX. Multireceptor Somatotropin-Release Inhibitory Factor Analogs

### A. Evolution of Concepts

**Fig. 23.**
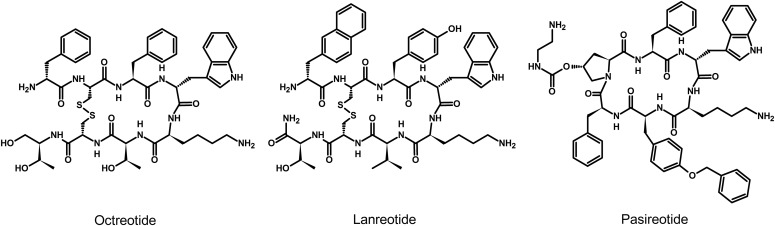
Structures of SRIF ligands currently used in clinical practice.

The rationale for desired characteristics of therapeutically useful SRIF analogs has evolved as knowledge of receptor subtypes and their interactions has become available. Following the discovery of SRIF as the hypothalamic factor responsible for suppression of GH secretion (Brazeau et al., 1973), it became apparent that it was involved in multiple additional physiologic functions (Reichlin, 1983a,b). Due to the rapid degradation and clearance of the native SRIF peptide, efforts were focused on creating analogs with increased metabolic stability that would be useful for treating conditions of excess GH secretion, most notably acromegaly. As the structure of native SST was modified, differences were observed in the ratio of GH-suppressing activity versus other actions, in particular the suppression of insulin, which was considered a potential problem for therapeutic application (Grant et al., 1976; Brown et al., 1977; Meyers et al., 1977; Coy et al., 1978). Through screening in rodents, analogs were identified with potent GH-suppressing activity with acceptably low insulin-suppressing activity (Bauer et al., 1982; Heiman et al., 1987), including the two SRIF analogs still most widely used clinically for treatment of acromegaly and NETs, octreotide (Sandostatin) and lanreotide (Somatuline) (Fig. 23; Table 9).

**TABLE 9:** Ligand-binding affinities for approved and investigational SRIF ligands

	SST_1_	SST_2_	SST_3_	SST_4_	SST_5_
Octreotide*^a^*	>1000	0.4	4.4	>1000	5.6
Lanreotide*^b^*	2129	0.75	98	1826	5.2
Pasireotide*^c^*	9.3	1	1.5	>100	0.16
Veldoreotide*^d^*	>1000	3	>100	7	6

^a^ Data from Reisine and Bell (1995), Patel (1999).

^b^ Data from Shimon et al. (1997b), Zatelli et al. (2001).

^c^ Data from Bruns et al. (2002).

^d^ Data from Afargan et al. (2001).

The reason that the GH and insulin-suppressing activities could be dissociated by structural modification of SRIF was later explained with the identification of five distinct SSTs (Reisine and Bell, 1995). With the realization that there were multiple receptor subtypes, the concept emerged that different subtypes controlled different, specific biologic activities, and that functional selectivity might be achieved by analogs with preference or, ideally, selectivity for one specific receptor subtype. The task then became determining which receptor subtype controlled which specific function. This was approached by creating libraries of SRIF analogs that were fully characterized for their preferences or selectivity for the different receptor subtypes. By screening panels of analogs with varying selectivities in different biologic models, it was hoped that the receptor subtype responsible for a particular biologic action could be identified, and could thus become the basis for therapeutically useful compounds targeting a specific function.

One of the first questions to be addressed by this approach was the involvement of the SSTs in suppressing GH secretion in humans. Although the two earlier discovered analogs, octreotide and lanreotide, are the most widely used medical therapy for acromegaly, they do not normalize GH and IGF-1 levels in a significant percentage of patients with acromegaly. A recent meta-analysis of clinical studies with sustained release octreotide and lanreotide indicated normalization of GH and IGF-1 in 56% and 55%, respectively, of treated patients with acromegaly (Carmichael et al., 2014); however, most of the included studies did not use the current normalization criteria of GH <1% and did not consider composite GH and IGF-1 normalization (Gadelha et al., 2017). The PRIMARYS study, which assessed treatment-naive, unselected acromegaly patients, found that only 30% of patients were fully controlled by these clinically approved somatostatin receptor ligands (Caron et al., 2014).

With characterization of their interaction with the five SSTs, both lanreotide and octreotide were found to have potent subnanomolar affinity for the SST_2_ subtype, with moderate affinity for SST_5_ (Table 9). Lanreotide and octreotide also have moderate affinity for SST_5_ receptors, but that does not translate functionally (Siehler et al., 1998; Siehler and Hoyer, 1999a,b). Not surprisingly, studies using subtype-selective analogs confirmed that the SST_2_ subtype was indeed responsible for suppression of GH in the rat, the species used for biologic optimization of lanreotide and octreotide (Raynor et al., 1993b; Briard et al., 1997). In contrast, however, when ligand panels were tested in cultures of human fetal pituitaries, it was found that activation of both SST_2_, as well as the SST_5_ subtype, induced suppression of GH secretion, and that activation of either of the two receptors was equally efficacious (Shimon et al., 1997a). Furthermore, when the two receptors were activated together, GH-suppressing activity was significantly enhanced, well beyond that observed with the clinically used SST_2_-selective analogs, lanreotide and octreotide (Shimon et al., 1997b). Considering that native SRIF potently interacts with all five receptor subtypes, it is reasonable to assume that the enhanced suppression of GH induced by dual receptor activation is the normal mechanism employed by SRIF in the physiologic control of GH. These observations also suggest that the lack of potent SST_5_ activity in the clinically used analogs may be the reason for the lack of full control of GH and IGF-1 in a significant number of patients with acromegaly.

The need for greater SST_5_ activation for a more complete suppression of GH was confirmed in studies of cultures of pituitary adenoma cells from patients with acromegaly classified as only partially responsive to SRIF analog therapy (Jaquet et al., 2000). In keeping with clinical experience, the SST_2_-preferring analog, octreotide, induced only a partial suppression of GH in the cultured adenoma cells. Treatment with more potent SST_2_- or SST_5_-selective analogs produced somewhat greater suppression. However, combined SST_2_- and SST_5_-selective analog treatment to activate both receptor subtypes produced greatly enhanced suppression of GH, thus supporting the concept that greater activity at SST_5_ is required to normalize GH in a greater percentage of acromegaly patients than is achieved with lanreotide and octreotide (Jaquet et al., 2000).

Based on these observations, the first multisubtype-selective analog, BIM-23244, was produced with 2× greater activity at SST_2_, and 20× greater activity at SST_5_, as compared with octreotide and lanreotide (Saveanu et al., 2001). To test the feasibility of biselective receptor activity in a single compound, GH-secreting tumors were collected from 10 patients with acromegaly that were clinically classified as either fully or only partially responsive to current SRIF analog therapy. Cultured adenoma cells from the fully responsive patients responded equally to treatment with either octreotide or the SST_2+5_ biselective analog, BIM23244; however, octreotide produced only a partial response in cells from partially responsive patients, whereas BIM-23244 produced a greatly enhanced response, similar to that observed in the adenoma cells from fully responsive patients (Saveanu et al., 2001). These results substantiate the benefit from dual activation of both receptor subtypes and validate the concept that both activities can be presented in a single compound and retain the enhanced biologic action.

These observations opened the possibility of receptor subtype interactions in other tissues and biologic functions. Studies of gene receptor expression had already demonstrated that various tissues contained multiple SSTs, and that expression of these subtypes could change depending on the physiologic conditions or as a result of pathology (Bruno et al., 1993; Patel et al., 1996; Reubi et al., 1997; Kimura et al., 2001). An example of the latter is the shift in SRIF suppression of prolactin secretion from SST_2_-mediated in normal human pituitary cells to SST_5_-mediated in cells from human prolactinomas (Saveanu et al., 2001). In addition, the expression of SSTs can change temporally during the course of a specific physiologic or pathologic process. Khare et al. (1999) demonstrated that, following damage to the endothelial lining of the aorta, the pattern of SSTs expressed changed over the subsequent days as the various stages of repair occurred. Observations such as these raised the possibility that multireceptor-interacting ligands could not only produce enhanced actions, but by targeting selected combinations of receptor subtypes, might also provide selectivity for specific physiologic or pathologic states.

### B. Potential Mechanisms

The mechanism by which SRIF action is enhanced by activating a combination of receptors remains uncertain. The simplest possibilities would be activation of two separate transduction pathways that have a common biologic endpoint, or greater activation of a single transduction pathway shared by two or more receptors. However, another possibility with a growing body of evidence is that various receptors can physically interact to form homo- or heterodimers, with resulting changes in activity. This phenomenon was first reported to occur for members of the opioid receptor family, which are structurally related to SSTs. Jordan and Devi (1999) reported that heterodimers formed by the *κ* and *δ* opioid receptors resulted in unique ligand-binding properties and, when activated, a potentiation of signal transduction. Formation of both homo- and heterodimers has subsequently been reported to occur among the SSTs as well, including SST_2_ and SST_5_. Dimerization of receptors with resulting alterations in ligand interactions suggests a unique opportunity to develop analogs that recognize only the specific homodimer of one receptor or the heterodimer of multiple receptor types, to achieve the highest level of functional specificity and efficacy.

The approach of using subtype-selective analogs to determine which SSTs are involved in a particular biologic action has continued using different models. In some cases, single-receptor subtypes do appear to be the dominant mechanism. Examples include suppression of insulin mediated by SST_5_ on human *β*-cells (Zambre et al., 1999), glucagon by SST_2_ on *α*-cells (Strowski et al., 2000), and vessel out-sprouting by SST_1_ from cultured human placental vein explants, a model of angiogenesis (Bocci et al., 2007). As direct suppression of insulin is mediated by SST_5_, the early observation that certain analogs could suppress GH while having minimal effect on insulin explains the modest amount of SST_5_ activity of the analogs selected for clinical use, lanreotide and octreotide.

As studies continued examining combinations of receptor subtype activation for potential enhancement, it was observed that receptor subtype interactions can also be antagonistic. Testing the effect of SST_2_ and 5 activation on proliferation of thyroid medullary carcinoma cells, it was observed that SST_2_-selective analogs induce dose-related inhibition of proliferation, whereas SST_5_-selective analogs cause an increase (Zatelli et al., 2001). When combined, increasing concentrations of the SST_5_-selctive analog prevents the suppression of proliferation by the SST_2_-selective analog, in a dose-related manner, such that at equimolar concentrations the effect of both is neutralized. In this instance, coactivation of SST_2_ and _5_ results in an antagonistic interaction, as opposed to the enhanced biologic effect observed by coactivation of these same receptors on GH secretion. These results indicate that the biologic consequence of receptor subtype interaction is not only a function of the receptors involved, but also the specific cell type or tissue in which they are expressed.

A partial explanation of the antagonistic effects of certain SSTs may be the inactivation of one or both receptors as a result of conformational changes following heterodimerization. In studies examining expression and function of SST_2_ and SST_3_ receptors expressed individually, Pfeiffer et al. (2001) demonstrated homodimerization of both subtypes and induction of specific transduction mechanisms when activated. When coexpressed, however, heterodimerization between the two subtypes was observed with the consequence of retained activation and signal transduction with SST_2_-selective ligands, but a complete loss of activation and signaling with SST_3_-selective ligands. These results clearly illustrate the exponential increase in complexity in moving from the initial targeting of individual receptor subtypes to affect a specific function to the targeting of various subtype combinations.

Although complex, the concept of targeting the interaction between multiple receptor subtypes remains attractive for enhanced efficacy; however, due to the widespread distribution of SSTs in different tissues, the original concern still remains that a metabolically stable compound able to activate multiple receptor subtypes could induce unwanted side effects. As an example, although the previously described biselective analog, BIM-23244, with selective, potent interaction with both SST_2_ and _5_, yields superior GH suppression, and potentially greater therapeutic benefit for a wider range of patients suffering from acromegaly, the direct suppression of insulin by SST_5_ raises the potential for unwanted pancreatic side effects. To examine the consequence of activating SST_2_ versus SST_5_, a study was conducted in healthy volunteers in which a SRIF analog with potent, selective SST_2_ activity was compared with a potent SST_5_ analog. Infusion of the SST_2_ analog resulted in a dose-related decrease in glucagon and insulin, but without effect on glucose levels. In addition, administration of an amino acid challenge during the SST_2_ analog infusion resulted in an appropriate insulin response, again maintaining normal glycemic control. Infusion of the SST_5_ analog, however, resulted in a dramatic suppression of insulin secretion, with resulting hyperglycemia, and failure of the *β*-cells to respond to the amino acid challenge. As a result of this potentially severe side effect mediated by SST_5_, BIM-23244 was not further developed, and design of subsequent analogs aimed for modest interaction with SST_5_. These results illustrate that the original concern of potential side effects when activating multiple receptors is a legitimate consideration.

### C. Pasireotide

A different concept from teasing out the involvement of specific SSTs in specific functions with the idea of creating subtype-specific, and therefore function-specific analogs, was to create clinically useful receptor ligands that are metabolically stable, but mimic the ability of native SRIF to interact with all five receptor subtypes. A theoretical advantage of this approach is that, assuming the activities are correctly proportioned, it should be possible to take advantage of receptor subtype interactions to produce different or enhanced responses from those achieved by activation of a single receptor subtype. The risk is that receptors mediating unwanted effects could also be activated. This approach was exemplified by development of the pan-receptor–specific ligand, pasireotide (also known as Signifor), a cyclohexapeptide with reasonably high affinity for SST_1_, _2_, and _3_, no interaction with SST_4_, but exceptionally high, subnanomolar affinity for SST_5_ (Table 9) (Bruns et al., 2002). In studies in rats, pasireotide produced comparable suppression of GH secretion as octreotide, but with much greater duration owing to a significantly greater circulating *t*_1/2_. In long-term infusion studies, pasireotide was considerably more effective in lowering IGF-1 than octreotide (Bruns et al., 2002). Because IGF-1 production is regulated by both GH-dependent and independent mechanisms, the enhanced action of pasireotide may be the result of interaction with receptor subtypes other than SST_2_, which is the primary mediator of octreotide action.

In a clinical trial directly comparing sustained release formulations of pasireotide and octreotide in a large cohort of randomly allocated, medically naive patients with acromegaly, pasireotide was found to control GH and IGF-1 in a greater percentage (31.3% pasireotide treatment versus 19.2% octreotide treatment) of subjects (Colao et al., 2014). Furthermore, in patients resistant to octreotide or lanreotide treatment, sustained-release pasireotide was found to achieve control in 15%–20% of subjects, depending on dosage (Gadelha et al., 2014). These results may be explained by the study of Gatto et al. (2017), in which pasireotide was compared with octreotide for their ability to suppress GH secretion from in vitro cultures of tumor cells from acromegalic patients. Overall, the two were equivalent; however, pasireotide was found to be more effective than octreotide in a subgroup of the cultures from tumors with a comparatively lower expression of SST_2_ and SST_2_/SST_5_ ratio (Gatto et al., 2017). In keeping with the high activity of pasireotide at SST_5_, however, a significantly higher percentage of patients experienced hyperglycemia-related adverse events in the two clinical studies with pasireotide treatment as compared with octreotide or lanreotide treatment (Colao et al., 2014; Gadelha et al., 2014). The potential of pasireotide has also been investigated in Cushing’s disease.

### D. Dopastatin

Extending further the concept of multireceptor SRIF ligands is the creation of compounds that interact with SSTs as well as receptors outside the SST family. This concept originally derived from clinical studies indicating that combined treatment of acromegalic patients with both SRIF and dopamine analogs resulted in greater control of GH and IGF-1 than the use of either agent alone (Fløgstad et al., 1994; Minniti et al., 1997; Marzullo et al., 1999; Li et al., 2000). This generated the idea to create chimeric compounds that contain structural elements of both SRIF and dopamine, and that retain the ability to bind to receptors of both. In initial studies in primary cultures of human GH-secreting adenoma cells, it was observed that whereas both pure SRIF and pure dopamine analogs were able to induce dose-related suppression of GH secretion, the combination of the two individual agents produced no greater suppression of GH than the SRIF analog alone. However, when both activities were combined in a single, chimeric compound, able to interact with both the SST_2_ and D_2_ receptor, significantly enhanced potency as well as efficacy is observed (Saveanu et al., 2002; Jaquet et al., 2005). The mechanism for this enhanced activity remains unknown; however, one possible explanation is the reported demonstration of heterodimer formation between both SST_2_ and _5_, and D_2_ receptor (Rocheville et al., 2000a).

Further refinement of the ratio of activities in the chimeric compound resulted in the production of BIM-23A760, which binds to SST_2_ (0.03 nM), SST_5_ (42 nM), and D_2_ receptor (16 nM) (Jaquet et al., 2005). The modest affinity at SST_5_ is intentional to avoid potential pancreatic effects, as previously discussed. The lack of glycemic side effects was confirmed in normal cynomolgous monkeys in which administration of BIM-23A760 produced potent, dose-related suppression of GH and IGF-1, but had no effect on either insulin secretion or circulating glucose. In addition to suppression of secretion, BIM-23A760 has been demonstrated to have potent antiproliferative effects, producing dose-related suppression of cultured primary human nonfunctioning pituitary adenoma cells (Florio et al., 2008) and somatotropinoma cells (Ibáñez-Costa et al., 2017a). Furthermore, complete arrest of spontaneously developing, aggressive, nonfunctioning pituitary adenomas was observed in vivo in proopiomelanocortin KO mice, an effect not observed with pure SRIF or dopamine analogs, either alone or in combination. These results suggest that chimeric compounds, such as BIM-23A760, may be effective in controlling pituitary diseases of hypersecretion, as well as impacting the growth of the underlying causative tumor.

Clinical development of BIM-23A760 was initiated and produced a clean safety profile in phase I and a significant demonstration of efficacy in a phase IIa, single-dose study in acromegalic subjects. Unfortunately, with repeated administration in humans, a long-acting, highly potent dopaminergic metabolite was produced that gradually accumulated and diminished the action of the parent compound, BIM-23A760. Subsequently, after further structure–activity studies, a second generation chimera, BIM-23B065, was produced with significantly greater potency and efficacy than BIM-23A760, as demonstrated by suppression of GH secretion from primary cultures of human GH-secreting adenoma cells from patients classified as both fully and only partially responsive to the currently used SRIF analogs, octreotide and lanreotide, and without formation of an interfering metabolite. BIM-23B065 is currently in early development.

From the initial discovery of SRIF and its receptor subtypes, the rationale for therapeutically useful analogs has evolved from targeting a single-receptor subtype to control a specific function and to limit potential side effects, to targeting specific combinations of receptors to induce enhanced effects for a specific function. Although progress has been made in terms of elucidating specific, disease-related combinations of receptors that act together, the initial concern of inducing side effects through activation of receptors in nontargeted tissues has been demonstrated to be a legitimate consideration. Future analogs that can specifically interact with targets, such as the unique binding pockets of homo- and heterodimers formed from the SST subtypes, as well as other receptor families, may yet achieve the full potency, selectivity, and safety potential envisioned for SRIF analogs.

## X. Somatotropin-Release Inhibitory Factor Analogs in Current Clinical Practice

Hypothalamic SRIF traverses the hypothalamic–pituitary portal vessels to impinge on anterior pituitary cells that express multiple SSTs. SRIF analogs show target selectivity for receptor subtype and functional selectivity in regulating GH, ACTH, and TSH secretion (Shimon et al., 1997b). Somatotroph cells predominantly express SST_2_ > SST_5_. SST_2_ signals to suppress GH secretion and may also regulate somatotroph tumor growth, whereas SST_5_ predominantly suppresses corticotroph ACTH release (Table 1). Studies with human GH-secreting tumor cell cultures showed a similar receptor profile and functional response to SRIF analogs (Shimon et al., 1997a). TSH-secreting pituitary adenomas (TSHomas) express SST_2_ and SST_5_ (Gatto et al., 2012). Subsequent studies revealed that anterior pituitary SSTs may also signal in a ligand-independent action (Ben-Shlomo et al., 2005; Vlotides et al., 2006). Thus, constitutive SST signaling may regulate ambient pituitary hormone secretion to maintain tonic hormone control in the absence of SRIF. These observations have supported the development of therapeutic molecules targeting different SSTs. SRIF analogs with higher affinity for SST_2_ are more efficacious for control of GH hypersecretion in acromegaly or TSH hypersecretion from thyrotropinomas (Melmed, 2003). In contrast, pasireotide, which exhibits an affinity-binding profile more similar to natural SRIF-14 (Weckbecker et al., 2002; Ben-Shlomo et al., 2009a), is particularly suitable for suppressing ACTH in patients with pituitary-dependent Cushing disease.

### A. Treatment

#### 1. Acromegaly

The SST_2_ subtype is preferentially expressed on somatotroph cell surfaces and regulates GH secretion by suppressing intracellular cAMP levels (Greenman and Melmed, 1994a,b; Shimon et al., 1997b). SRIF analog formulations with high SST_2_ affinity employed for treating acromegaly, namely octreotide and lanreotide, have proven safe and effective for long-term acromegaly management (Table 10). Octreotide, an octapeptide, inhibits GH secretion with a potency 45 times greater than endogenous SRIF, with minimal suppression of insulin release (Lamberts, 1988). As the molecule is relatively resistant to enzymatic degradation, the in vivo *t*_1/2_ is prolonged (up to 2 hours) after s.c. injection. Lanreotide is a structurally related eight-amino-acid cyclic peptide (Castinetti et al., 2009). Responsiveness to both compounds correlates with GH-secreting adenoma SST_2_ expression (Casarini et al., 2009). Rebound GH hypersecretion that occurs after SRIF infusion is not apparent after administration of either peptide, offering unique advantages for safe, long-term acromegaly therapy (Lamberts et al., 1996). Pasireotide exhibits a preferential high affinity to SST_5_ (39-fold higher than octreotide), and also binds to SST_1_, SST_2_, and SST_3_. Octreotide and pasireotide similarly inhibited free cytosolic calcium and GH release in vitro, in human somatotropinoma cell cultures, where they also comparably reduced GH mRNA levels and cell viability (Ibanez-Costa et al., 2016). Indeed, using cultures derived from 33 in vitro human pituitary tumors in a head-to-head study, octreotide and pasireotide exhibited equivalent antisecretory efficacy in suppressing GH (Gatto et al., 2017).

**TABLE 10:** Approved and investigational SRIF analogs blocking GH secretion Data adapted from Melmed (2016).

Agent	Description	Regulatory Status
Lanreotide autogel	Long-acting lanreotide	Available
	Administered via deep s.c. injection every 4–6 wk	
Octreotide LAR	Long-acting octreotide	Available
	Administered via i.m. injection every 4 wk	
Pasireotide LAR	Long-acting pasireotide	Available
	Administered via i.m. injection every 4 wk	
Octreotide capsules	Octreotide encapsulated with transient permeability enhancer	Completed phase 3
	Administered orally twice daily	
CAM2029	Octreotide bound in liquid crystal matrix	In phase II
	Administered via s.c. injection at a frequency not yet determined but likely to be every 4 wk	
Veldoreotide (COR-005)	SRIF analog highly selective for GH suppression	In phase II
	Administered via i.m. injection at a frequency not yet determined but likely to be every 4 wk	

##### a. Effects on biochemical control

Both lanreotide and octreotide exhibit similar clinical efficacy and side-effect profiles (Murray and Melmed, 2008). When defining disease outcomes, it is apparent that up to 40% of patients receiving SRIF analogs exhibit discordant GH and IGF-1 levels. Measuring IGF-1 levels, rather than GH levels, during an oral glucose tolerance test appears to more rigorously reflect disease control (Carmichael et al., 2009). Injectable depot SRIF analog formulations are safe and long-acting and enable maximal biochemical control. Drug levels peak 28 days after injection of sustained release i.m. microsphere preparation of octreotide long-acting release (LAR) (20–30 mg) (Fløgstad et al., 1997; Lancranjan et al., 1999), with concomitant GH levels suppressed for up to 49 days. In an open-label study, 70% of 151 patients responsive to octreotide showed GH levels suppressed to <2.5 ng/ml (Lancranjan et al., 1999). Similarly, GH <2 ng/ml and normal IGF-1 levels were achieved in 70% of 36 patients followed for up to 18 years (Maiza et al., 2007). The water-soluble lanreotide autogel (60, 90, or 120 mg) administered by deep s.c. injection every 28–42 days suppressed GH to <2.5 ng/ml in 130 patients at 1 year (Neggers et al., 2015). In a randomized 12-month study, 358 patients received pasireotide LAR (40 mg) or octreotide LAR (20 mg), and biochemical control was achieved in 31% and 19% of subjects, respectively (Colao et al., 2014). Among those resistant to maximal doses of octreotide or lanreotide, 15% and 20% of resistant patients subsequently achieved control when placed on 40 or 60 mg pasireotide, respectively (Gadelha et al., 2014). Control of GH and IGF-1 levels is more favorable in patients with GH-secreting microadenomas (Ezzat et al., 1992). Octreotide, lanreotide, and pasireotide fall short of maximal treatment goals (i.e., normalized GH and IGF-1) in a large subset of patients. Despite medication adherence rates approaching 90% (Gurel et al., 2017), a global meta-analysis showed ∼55% control rates for both GH and IGF-1 with SRIF analogs (Carmichael et al., 2014).

##### b. Effects on disease comorbidities

SRIF analogs used as first-line therapy administered prior to surgery in selected patients may ameliorate preoperative morbidity, including heart failure or respiratory or metabolic disorders, thus enabling safer anesthesia (Colao et al., 2004). Furthermore, preoperative treatment may enhance the success of postoperative outcomes by shrinking large tumor masses prior to debulking procedures (Carlsen et al., 2008; Shen et al., 2010; Giustina et al., 2014; Katznelson et al., 2014). A subset of patients with minimally or noninvasive macroadenomas is most likely to benefit from preoperative therapy (Jacob and Bevan, 2014). In a meta-analysis of 64 reports, SRIF analogs were shown to significantly reduce GH-secreting pituitary tumor size (Giustina et al., 2012). Moreover, a meta-analysis showed a modestly beneficial effect of preoperative SRIF analogs on postoperative biochemical control (Pita-Gutierrez et al., 2013), but subsequent longer-term follow-up has not borne out these results (Fougner et al., 2014). Up to 60%–80% of patients harboring microadenomas, macroadenomas, and locally invasive tumors experience a reduction of pituitary adenoma size (Bevan, 2005; Freda et al., 2005; Giustina et al., 2012), with tumor shrinkage seen by 6 months of therapy initiation (Colao et al., 2016). The magnitude of shrinkage is variable, but some patients respond with >50% decrease in tumor mass. Given these observations, the use of preoperative SRIF analogs to improve surgical outcomes has been debated. Although studies have shown that postoperative biochemical control is in fact improved by presurgical SRIF analog treatment (Carlsen et al., 2008; Nunes et al., 2015), the overall evidence is dampened by the short follow-up duration and insufficient prospective evidence.

The beneficial impact of SRIF analogs on acromegaly comorbidities is variable, especially for cardiovascular dysfunction, and is determined by age, disease duration, and degree of biochemical disease control. Clinical benefits of SRIF analogs are achieved both by ameliorating deleterious effects of chronic GH and IGF-1 exposure, as well as likely reversal of fluid retention and swelling. For example, GH-induced epithelial sodium channel–dependent sodium transport actively leads to volume expansion and soft-tissue swelling, effects largely reversed by SRIF analogs (Kamenický et al., 2014). Hypertension, likely arising from chronic vascular damage, is usually not reversible by SRIF analogs. However, with biochemical control, doses of antihypertensive drugs required to normalize blood pressure may be decreased (Annamalai et al., 2013). Structural cardiac abnormalities, including myocardial hypertrophy and heart failure, are improved with biochemical control, especially in younger patients and in those with a shorter disease duration (Annamalai et al., 2013). Features of obstructive sleep apnea are usually improved with SRIF analog therapy (Annamalai et al., 2013), but the disorder may persist despite satisfactory biochemical control. Although joint pain and arthropathy are markedly improved symptomatically by SRIF analogs, structural joint damage and associated arthritis are usually irreversible, despite achievement of biochemical control. Headache is particularly responsive to short-acting SRIF analog therapy (Williams et al., 1987; Musolino et al., 1990; Levy et al., 2003; Marina et al., 2015).

As GH is a potent antagonist of insulin action, uncontrolled acromegaly is associated with insulin resistance, hyperglycemia, and eventually diabetes. SRIF analogs exert a dual effect on glucose control. As SST_5_ is expressed on the pancreatic *β*-cells, SRIF analogs with higher SST_5_ affinity (pasireotide > octreotide and lanreotide) suppress insulin secretion, leading to hyperglycemia and diabetes. By contrast, the potent GH suppression achieved by SST_2_-preferential SRIF analogs (octreotide and lanreotide) leads to enhanced insulin sensitivity and lowering (or normalizing) blood glucose levels.

##### c. Side effects

As SST_2_ and SST_5_ are ubiquitously expressed, especially in the GIT, it is not surprising that several off-target side effects are experienced. Transient abdominal pain, bloating, nausea, and diarrhea are commonly encountered. Asymptomatic gallstones, likely due to suppressed CCK and decreased gallbladder contractility, occur in about 20% of patients. Prolonged QT intervals have been associated with bradycardia, although distinguishing disease-related from drug-related heart conduction effects may be difficult. Elevated fasting glucose and glycated hemoglobin levels are rarely encountered (Mazziotti et al., 2009). Pasireotide leads to reversible insulinopenia, hyperglycemia, and diabetes in 30% or more of patients (Gadelha et al., 2014; Silverstein, 2016).

#### 2. Cushing Disease

Pituitary-dependent Cushing disease is caused by a corticotroph cell adenoma hypersecreting ACTH thus leading to adrenal cortisol overproduction (Biller et al., 2008). As corticotroph cells abundantly express SST_5_, pasireotide may suppress ACTH and features of hypercortisolemia in a subset of patients (Silverstein, 2016). In vitro, pasireotide inhibits basal and induced ACTH release from ACTH-secreting pituitary adenomas (Hofland et al., 2005; Batista et al., 2006). A double-blind, randomized phase III trial in 162 Cushing disease patients treated with pasireotide 600–900 *µ*g twice daily showed that median urinary-free cortisol (UFC) levels were suppressed by 50%, whereas ∼24% exhibited normalized UFC levels for 6 months. Patients with mildly elevated UFC levels are most likely to respond (Colao et al., 2012). Most patients not controlled within 8 weeks did not achieve control by study end. Of 75 patients with a demonstrable pituitary mass receiving 900 *µ*g pasireotide, 44% exhibited decreased mean pituitary tumor size. Blood pressure, weight, and quality of life improved, and triglyceride and low-density lipoprotein levels were reduced. Blood glucose and glycated hemoglobin levels increased in 118 of 162 patients, despite suppression of cortisol levels (Colao et al., 2012). As Cushing disease hypercortisolism is associated with insulin resistance and heart failure, monitoring of blood sugar and electrocardiograms for corrected QT interval prolongation and bradycardia is important.

#### 3. Thyroid-Stimulating Hormone-Secreting Pituitary Adenomas

Central hyperthyroidism is caused by a TSHoma, a rare disease occurring both in children and adults. TSHomas typically have strong SST_2_ and often SST_5_ expression and show a good response to first-generation SRIF analogs, with about 10% of cases showing resistance (Beck-Peccoz et al., 2013). There is a single case in which cure was achieved (Fliers et al., 2012). Pasireotide has also been used successfully in TSHoma (van Eersel et al., 2017).

#### 4. Neuroendocrine Tumors

Carcinoid, GI, and pancreatic NETs express cell surface SST_2_ (Öberg and Lamberts, 2016). These tumors exhibit significant morbidity and mortality, and at diagnosis fewer than 50% are surgically resectable (Kim et al., 2010). These tumors secrete 5-hydroxytryptamine or peptide hormones with significant clinical sequelae, including GI, bronchial, and cardiac dysfunction. Accordingly, SRIF analog therapy is aimed at decreasing or stabilizing tumor mass, as well as ameliorating adverse symptoms due to circulating hormones. Overall, survival of NET patients has improved about threefold since the introduction of SRIF analog therapy (Anthony et al., 1996; Yao et al., 2008).

In randomized double-blind trials, octreotide LAR and lanreotide autogel were shown to significantly ameliorate diarrhea or flushing in up to 80% of patients with carcinoid syndrome (Rubin et al., 1999; Modlin et al., 2006). In the placebo-controlled PROMID trial, when 85 patients with metastatic midgut NET received octreotide LAR, median time to tumor progression was extended from 6 to 14.3 months (Rinke et al., 2009). Furthermore, the disease was stabilized in two-thirds of patients receiving the SRIF analog therapy. In a 96-week trial by the Controlled Study of Lanreotide Antiproliferative Response in NET (CLARINET) of 204 patients randomized to receive placebo or lanreotide autogel (120 mg), prolonged disease-free survival was demonstrated (Caplin et al., 2014). Interestingly, combination treatment of octreotide with everolimus, a mechanistic target of rapamycin kinase inhibitor, exhibited additive efficacy benefit, i.e., tumor volume reduction, in 75% of patients versus 45% for those receiving placebo plus octreotide (Pavel et al., 2011). Based on these results, SRIF analogs appear to offer both symptomatic improvement as well as direct antitumor effects in patients harboring NET.

### B. Factors Influencing Somatotropin-Release Inhibitory Factor Analog Resistance

SRIF analog therapeutic efficacy rates vary depending on individual patient and tumor characteristics (Melmed, 2016). Understanding mechanisms driving SRIF analog responsiveness and resistance has enabled a personalized approach to acromegaly classification and management (Table 11) (Cuevas-Ramos et al., 2015). Retrospective studies have suggested predictors of acromegaly therapeutic responses, as well as markers of aggressive disease resistant to SRIF analogs that also correlate with adverse long-term outcomes. Increasing age, levels of GH and IGF-1, and tumor size are adverse determinants of SRIF analog responsiveness. As therapy is required to be open-ended, treatment duration is an important determinant of therapeutic sensitivity and control rates improve over years of treatment (Ayuk et al., 2002; Maiza et al., 2007).

**TABLE 11:** Markers of somatostatin receptor ligand responsiveness in GH-secreting pituitary adenomas Data adapted from Cuevas-Ramos et al. (2015).

GH Granulation	Dense vs. Sparse Using CAM5.2 Cytokeratin Immunostaining
SST_2_, SST_5_	Positive vs. negative expression
SST_2_:SST_5_	High vs. low ratio of average SST_2_ to SST_5_
SST_5_TMD4	Low vs. high expression
AIP	Lack vs. presence of mutation or high vs. low protein expression
*β*-arrestin	Low vs. high score based on intensity and expression pattern
Filamin A	High vs. low score based on intensity and expression pattern
*Gsp*	Presence vs. absence of mutation
*E-cadherin*	High vs. low score based on intensity and expression pattern

In general, SST_2_ tumor expression correlates with SRIF analog responsiveness. Several studies have correlated efficacy in GH-secreting adenomas with SST_2_ immunostaining (Takei et al., 2007; Fougner et al., 2008b; Casarini et al., 2009; Casar-Borota et al., 2013; Gatto et al., 2013b). Choice of rabbit mAbs (Lupp et al., 2011; Chinezu et al., 2014; Iacovazzo et al., 2016) to assess patterns and distribution of membrane staining is also associated with SRIF analog responsiveness (Iacovazzo et al., 2016). In acromegaly patients resistant to octreotide, tumors lacking SST_5_ immunoreactivity were resistant to pasireotide, whereas those with SST_5_ staining using the rabbit mAb UMB-4 had superior biochemical response (Iacovazzo et al., 2016). Cell culture responses to octreotide and pasireotide correlated with SST_2_ and SST_5_ expression, and lower SST_2_ expression was associated with superior pasireotide effects (Gatto et al., 2017). However, in other in vitro studies on unselected pituitary tumor cell culture, responses to octreotide and pasireotide did not show an evident correspondence with the SST_1_–SST_5_ profile (Ibanez-Costa et al., 2016). Nevertheless, tumors immunopositive for SST_2_ expression are more likely to respond to octreotide and lanreotide (Brzana et al., 2013), and those with a higher SST_2_ to SST_5_ ratio show improved outcomes (Casar-Borota et al., 2013). SST mutation has only been described in a single patient with acromegaly, in which there was a missense (Arg240Trp) variant in the *SSTR5* gene. The patient displayed resistance to octreotide (Ballare et al., 2001). The cytoskeletal actin-binding scaffolding protein filamin A regulates SST_2_ trafficking and stability (Treppiedi et al., 2017). Lack of filamin A is associated with reduced cell surface expression of SST_2_ in neuroendocrine cell lines (Najib et al., 2012), although this was not the case in somatotroph cells that could be associated with SRIF analog resistance (Peverelli et al., 2014; Treppiedi et al., 2017).

Disrupted receptor recycling also alters SST signaling on the cell surface. Although GH-secreting adenoma SST_2_ expression may be less abundant following pretreatment with SRIF analogs (Casar-Borota et al., 2013), this does not appear to result in drug resistance in acromegaly and TSH-secreting adenomas, whereas tachyphylaxis has been observed in patients with NETs (Toumpanakis and Caplin, 2013).

Tumors with large, dense GH granules diffusely distributed throughout the cytosol are typically more responsive to SRIF analogs than are those containing small, uniform GH granules (Melmed et al., 1983). Sparsely GH granulated somatotrophinomas express less SST_2_ and more SST_5_ and are more resistant to SRIF analogs than those that are densely granulated (Fougner et al., 2012; Kato et al., 2012; Brzana et al., 2013; Larkin et al., 2013). They are larger and more invasive and occur in younger patients (Mayr et al., 2013). Low E-cadherin expression also correlates with sparsely granulated adenomas and SRIF analog resistance (Fougner et al., 2010). On magnetic resonance imaging, T2-weighted hyperintense GH-cell adenomas are frequently sparsely granulated and associated with a poor response to SRIF analogs (Hagiwara et al., 2003; Puig-Domingo et al., 2010; Heck et al., 2016a,b). Hypointense adenomas are smaller and less invasive than hyper- and isointense adenomas, but interestingly exhibit higher IGF-1 levels (Potorac et al., 2015).

Molecular markers, including low aryl hydrocarbon receptor-interacting protein (AIP) expression (Jaffrain-Rea et al., 2013; Ritvonen et al., 2017), high *β*-arrestin expression (Gatto et al., 2013a), and presence of somatic mutation of the Gsp oncogene (Efstathiadou et al., 2015), have been associated with poor response to SRIF analogs, thus contributing to more adverse outcomes.

Octreotide stimulates *Zac1* mRNA expression, whereas *Zac1* knockdown renders cells unresponsive to SRIF analogs (Theodoropoulou et al., 2006). SRIF analogs upregulate AIP expression, and *Aip* mRNA correlates with *Zac1* expression (Chahal et al., 2012), establishing a novel pathway (Gadelha et al., 2013). As reduced AIP expression may be associated with reduced G_i*α*2_ levels (Tuominen et al., 2015; Ritvonen et al., 2017), SRIF analog resistance may be encountered in *AIP* mutation–positive patients (Leontiou et al., 2008; Daly et al., 2010; Oriola et al., 2012). Two truncated SST_5_ variants, SST_5_TMD4 with four TMD and sst_5_TMD5 with five TMD (Durán-Prado et al., 2010), may inhibit SST_2_ functions. SST_5_TMD4 correlates inversely with GH and IGF-1 reductions in response to octreotide LAR therapy (Durán-Prado et al., 2010; Luque et al., 2015).

Somatic guanine nucleotide–binding protein G_s_ subunit *α* gene mutations, occurring in about 20%–30% of somatotrophinomas, result in smaller, less invasive, and more densely granulated tumors, are more often seen in older patients, and respond more favorably to SRIF analogs (Landis et al., 1990; Barlier et al., 1998; Larkin et al., 2013). Guanine nucleotide–binding protein G_s_ subunit *α* gene-positive patients have an approximately 10% greater reduction in GH levels in response to octreotide (Efstathiadou et al., 2015). In contrast, low levels of rapidly accelerated fibrosarcoma kinase inhibitory protein correlated with reduced octreotide responsiveness (Fougner et al., 2008a).

About 20% of patients with Cushing disease achieve biochemical normalization with pasireotide (Colao et al., 2012). Corticotroph adenomas express high levels of SST_5_, followed by SST_2_ (Batista et al., 2006; de Bruin et al., 2009; Tateno et al., 2009; Lupp et al., 2011; van der Pas et al., 2013). As glucocorticoids may suppress SST_2_ expression (de Bruin et al., 2009), corticotrophinomas are usually resistant to octreotide or lanreotide. Although NETs usually express SST_2_, insulinomas have reduced expression of SST_2_ compared with other NETs (Hofland and Lamberts, 2003). SST_2_ is expressed in 90% of GI NETs, except insulinomas, where 50% of tumors express the receptor (Toumpanakis and Caplin, 2013). Tachyphylaxis has been described at variable time intervals after commencement of treatment, with reports ranging from 3 to 27 months (Toumpanakis and Caplin, 2013).

Assessment of clinical, imaging, biochemical, and histopathological markers therefore offers a personalized approach to predict biochemical outcomes with SRIF analogs (Puig Domingo, 2015; Melmed, 2016). Accordingly, rigorous phenotypic classification of acromegaly biomarkers for disease staging has been applied to further refine treatment approaches (Cuevas-Ramos et al., 2015; Giustina et al., 2016).

### C. Somatotropin-Release Inhibitory Factor–Based Radiopharmaceuticals

#### 1. Radiolabeled Agonists

**Fig. 24.**
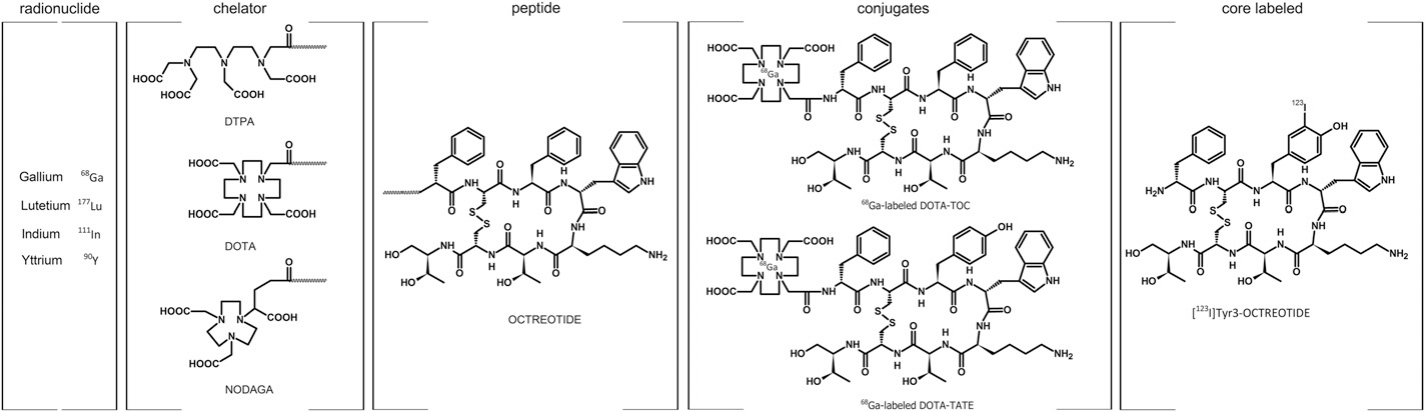
Structures of SST ligands used for scintigraphy. [^123^I]Tyr3-octroeotide, the very first compound for SST-targeted scintigraphy. Conjugation of DTPA to octreotide and labeling with indium-111 resulted in Octreoscan (Mallinckrodt), the first approved SST agent for SPECT imaging. Advanced Accelerator Application recently received market authorization for ^68^Ga-labeled DOTA-TOC (SomatoKit TOC) by the European Medicines Agency and for ^68^Ga-DOTA-TATE (Netspot) by the FDA. It is expected that [^177^Lu]DOTATATE will soon be approved by FDA and European Medicines Agency as first agent for peptide receptor radiotherapy.

Based on the metabolically stabilized synthetic octapeptide octreotide [d-Phe-Cys-Phe-d-Nal-Lys-Thr-Cys-Thr(ol)], which displays high affinity for SST_2_ and moderate affinity for SST_5_ and SST_3_, Krenning et al. (1989) synthetized the ^123^I-radioiodinated Tyr^3^ analog of octreotide ([^123^I]Tyr^3^-SMS 201-995, [^123^I]Tyr^3^-octreotide, [^123^I]TOC) (Fig. 24; Tables 12 and 13) and exploited this targeted radioligand for the first successful noninvasive single-photon emission computed tomography (SPECT) imaging of SST receptor–rich tumors in humans. This initial study on 10 patients is considered as pioneering work in the field of SST imaging, and also as general proof-of-concept for the usefulness of peptide receptor imaging (PRI) and as a starting point for development of radiolabeled ligands for targeted PRRT. With the aim to overcome the unsuitable biodistribution of this first tracer, which was caused by high lipophilicity, predominant hepatobiliary excretion, and thus undesirably high abdominal background activity, the same group successfully developed 2 years later in collaboration with a group at Sandoz Research Institute a new derivative, [^111^In]diethylenetriaminepentaaceticacid-D-Phe^1^-octreotide ([^111^In]DTPA-D-Phe^1^-octreotide), named [^111^In]pentetreotide (Bakker et al., 1991a,b) (Fig. 24). Subsequently, the favorable properties of this agent (e.g., ease of preparation, appropriate *t*_1/2_, and absence of major accumulation in the upper abdominal region due to its renal clearance) were demonstrated in a comparative evaluation in humans (Krenning et al., 1992). For the first time, these studies introduced radiometals into the concept of PRI and PRRT, which significantly simplified the preparation of SST-targeted radiopharmaceuticals by exploiting fast and simple complexation procedures using chelator-conjugated peptide precursors, e.g., DTPA conjugated to the N-terminal d-Phe^1^-amino acid of the peptide as in [^111^In]pentetreotide. Data on [^111^In]pentetreotide imaging in more than 1000 patients were published in 1993 (Krenning et al., 1993), and this is still the most frequently cited paper from the *European Journal of Nuclear Medicine*. Because the sensitivity and specificity of [^111^In]pentetreotide SPECT in patients with GEP-NETs were higher than those obtained with the classic imaging modalities computer tomography or magnetic resonance imaging, [^111^In]pentetreotide (OctreoScan; Mallinckrodt, Staines-Upon-Thames, U.K.) was approved by the Food and Drug Administration (FDA) in 1994 as the first peptide-based imaging radiopharmaceutical on the basis of a dataset obtained in 350 European patients.

**TABLE 12:** Ligand-binding affinities of SRIF-based radiochemicals

	SST_1_	SST_2_	SST_3_	SST_4_	SST_5_	Regulatory Status
Agonists with Predominant SST_2_ Affinity						
DTPA-octreotide*^a^*	>10,000	12 ± 2	376 ± 84	>1000	299 ± 50	
DOTA-lanreotide*^a^*	>10,000	26 ± 3.4	771 ± 229	>10,000	73 ± 12	
In-DTPA-OC*^a^*	>10,000	22 ± 3.6	182 ± 13	>1000	237 ± 52	FDA approved
DOTA-TOC*^a^*	>10,000	14 ± 2.6	880 ± 324	>1000	393 ± 84	
Y-DOTA-TOC*^a^*	>10,000	11 ± 1.7	389 ± 135	>10,000	114 ± 29	Phase II studies
DOTA-OC*^a^*	>10,000	14 ± 3	27 ± 9	>1000	103 ± 39	
Y-DOTA-OC*^a^*	>10,000	20 ± 2	27 ± 8	>10,000	57 ± 22	
Ga-DOTA-TOC*^a^*	>10,000	2.5 ± 0.5	613 ± 140	>1000	73 ± 21	EMA approved
Ga-DOTA-OC*^a^*	>10,000	7.3 ± 1.9	120 ± 45	>1000	60 ± 14	
DTPA-TATE*^a^*	>10,000	3.9 ± 1	>10,000	>1000	>1000	
In-DTPA-TATE*^a^*	>10,000	1.3 ± 0.2	>10,000	433 ± 16	>1000	
DOTA-TATE*^a^*	>10,000	1.5 ± 0.4	>1000	453 ± 176	547 ± 160	
Y-DOTA-TATE*^a^*	>10,000	1.6 ± 0.4	>1000	523 ± 239	187 ± 50	
In-DOTA-TOC*^b^*	>10,000	4.6 ± 0.2	120 ± 26	230 ± 82	130 ± 17	
Ga-DOTA-TATE*^a^*	>10,000	0.2 ± 0.04	>1000	300 ± 140	377 ± 18	FDA approved
Lu-DOTATATE*^c^*	>1000	2.0 ± 0.8	162 ± 16	>1000	>1000	Phase III completed
I-Gluc-TOC*^d^*	—	2.2 ± 0.7	357 ± 22	—	64 ± 24	
I-Gluc-TTE*^d^*	—	2.0 ± 0.5	>1000	—	521 ± 269	
I-Gluc-S-TATE*^d^*	—	2.0 ± 0.7	398 ± 19	—	310 ± 156	
I-Gal-S-TATE*^d^*	—	2.0 ± 0.8	491 ± 63	—	413 ± 167	
Gluc-Lys(FP)-TATE*^e^*	>10,000	2.8 ± 0.4	>1000	437 ± 84	123 ± 8.8	
Agonists with Pansomatostatin-Like Binding Profile						
Ga-DOTA-NOC*^f^*	>10,000	1.9 ± 0.4	40.0 ± 5.8	260 ± 74	7.2 ± 1.6	Phase II studies
In-DOTA-NOC*^g^*	>1000	3.3 ± 0.3	26 ± 1.9	>1000	10.4 ± 1.6	
In-DOTA-BOC*^g^*	>10,000	3.1 ± 0.3	12 ± 1.0	455 ± 65	6 ± 1.8	
NOC-ATE*^b^*	>1000	3.6 ± 1.6	302 ± 137	260 ± 95	16.7 ± 9.9	
BOC-ATE*^b^*	>1000	0.8 ± 0.4	33 ± 5.5	80 ± 20	3.6 ± 1.5	
In-DOTA-NOC-ATE*^b^*	>10,000	2 ± 0.35	13 ± 4	160 ± 3.8	4.3 ± 0.5	
Lu-DOTA-NOC-ATE*^d^*	—	3.6 ± 0.3	31 ± 2	—	15 ± 1	
In-DOTA-BOC-ATE*^b^*	>1000	1.4 ± 0.37	5.5 ± 0.8	135 ± 32	3.9 ± 0.2	
Lu-DOTA-BOC-ATE*^d^*	—	2.4 ± 0.3	11 ± 1	—	8.3 ± 0.4	
KE108*^h^*	0.96 ± 0.15	0.4 ± 0.04	0.44 ± 0.06	0.6 ± 0.03	0.26 ± 0.04	
KE121*^h^*	1.6 ± 0.7	0.5 ± 0.2	0.3 ± 0.1	0.4 ± 0.2	0.2 ± 0.1	
Y-DOTA-K121*^h^* (Y-KE88)	2 ± 0.8	4.3 ± 0.8	0.7 ± 0.2	0.5 ± 0.2	0.7 ± 0.2	
Ga-DOTA-K121*^h^* (Ga-KE88)	3.5 ± 1.6	1.8 ± 1.6	0.8 ± 0.3	1.8 ± 0.5	0.9 ± 0.2	
Y-DOTAGA-KE121*^h^* (Y-KE87)	6.7 ± 2.1	2.7 ± 2.4	0.6 ± 0.1	1.6 ± 0.6	1.3 ± 0.4	
Antagonists						
In-DOTA-BASS*^i^*	>1000	9.4 ± 0.4	>1000	380 ± 57	>1000	Pilot study
In-DOTA-JR11*^j^*	>1000	3.8 ± 0.7	>1000	>1000	>1000	Pilot study
Ga-DOTA-JR11*^j^* (Ga-OPS201)	>1000	29 ± 2.7	>1000	>1000	>1000	Pilot study
Ga-NODAGA-JR11*^j^* (Ga-OPS202)	>1000	1.2 ± 0.2	>1000	>1000	>1000	Phase I/II study
Lu-DOTA-JR11*^j^* (Lu-OPS201)	>1000	0.73 ± 0.15	>1000	>1000	>1000	
sst_3_-ODN-8*^d^*	—	>1000	6.7 ± 2.6		>1000	
DOTA-sst_3_-ODN-8*^g^*	>1000	>1000	5.2 ± 1.3	>1000	>1000	
In-DOTA-sst_3_-ODN-8*^g^*	>1000	>1000	15 ± 5.2	>1000	>1000	

EMA, European Medicines Agency.

^a^ Data from Reubi et al. (2000a).

^b^ Data from Ginj et al. (2005).

^c^ Data from Schottelius et al. (2015).

^d^ Data from Cescato et al. (2006).

^e^ Data from Wester et al. (2003).

^f^ Data from Antunes et al. (2007).

^g^ Data from Ginj et al. (2006a).

^h^ Data from Ginj et al. (2008).

^i^ Data from Ginj et al. (2006b).

^j^ Data from Fani et al. (2012).

**TABLE 13:** Amino acid sequences of SRIF-based radiochemicals Amino acids: first letter capitalized: L-amino acid; first letter in lowercase: D-amino acid.

Ligand	Chelator/Prosthetic Group	AA1	AA2	AA3	AA4	AA5	AA6	AA7	AA8	AA9	AA10
Peptide Agonists with Predominantly SST_2_ Affinity											
Octreotide	—			phe	Cys	Phe	trp	Lys	Thr	Cys	Thr-ol
Tyr3-octreotide (TOC)				phe	Cys	Tyr	trp	Lys	Thr	Cys	Thr-ol
RC160 (Vapreotide)				phe	Cys	Tyr	trp	Lys	Val	Cys	Trp-NH_2_
Lanreotide (BIM-23014)				2-nal	Cys	Tyr	trp	Lys	Val	Cys	Thr-NH_2_
Radiopeptide Agonists and Precursors with Predominantly SST_2_ Affinity											
DTPA-octreotide	DTPA			phe	Cys	Phe	trp	Lys	Thr	Cys	Thr-ol
DOTA-lanreotide	DOTA			2-nal	Cys	Tyr	trp	Lys	Val	Cys	Thr-NH_2_
In-DTPA-OC	In-DTPA			phe	Cys	Phe	trp	Lys	Thr	Cys	Thr-ol
DOTA-TOC	DOTA			phe	Cys	Tyr	trp	Lys	Thr	Cys	Thr-ol
Y-DOTA-TOC	Y-DOTA			phe	Cys	Tyr	trp	Lys	Thr	Cys	Thr-ol
DOTA-OC	DOTA			phe	Cys	Phe	trp	Lys	Thr	Cys	Thr-ol
Y-DOTA-OC	Y-DOTA			phe	Cys	Phe	trp	Lys	Thr	Cys	Thr-ol
Ga-DOTA-TOC	Ga-DOTA			phe	Cys	Tyr	trp	Lys	Thr	Cys	Thr-ol
Ga-DOTA-OC	Ga-DOTA			phe	Cys	Phe	trp	Lys	Thr	Cys	Thr-ol
DTPA-TATE	DTPA			phe	Cys	Tyr	trp	Lys	Thr	Cys	Thr
In-DTPA-TATE	In-DTPA			phe	Cys	Tyr	trp	Lys	Thr	Cys	Thr
DOTA-TATE	DOTA			phe	Cys	Tyr	trp	Lys	Thr	Cys	Thr
Y-DOTA-TATE	Y-DOTA			phe	Cys	Tyr	trp	Lys	Thr	Cys	Thr
In-DOTA-TOC	In-DOTA			phe	Cys	Tyr	trp	Lys	Thr	Cys	Thr-ol
Ga-DOTA-TATE	Ga-DOTA			phe	Cys	Tyr	trp	Lys	Thr	Cys	Thr
Lu-DOTATATE	Lu-DOTA			phe	Cys	Tyr	trp	Lys	Thr	Cys	Thr
I-Gluc-TOC	Glucosyl			phe	Cys	3-I-Tyr	trp	Lys	Thr	Cys	Thr-ol
I-Gluc-TATE	Glucosyl			phe	Cys	3-I-Tyr	trp	Lys	Thr	Cys	Thr
I-Gluc-S-TATE	Glucosyl-S-			phe	Cys	3-I-Tyr	trp	Lys	Thr	Cys	Thr
I-Gal-S-TATE	Galactosyl-S-			phe	Cys	3-I-Tyr	trp	Lys	Thr	Cys	Thr
Gluc-Lys(FP)-TATE	Glucosyl-Lys(fluoropropionyl)			phe	Cys	3-I-Tyr	trp	Lys	Thr	Cys	Thr
Agonists toward Pansomatostatin-Like Binding Profile											
Ga-DOTA-NOC	Ga-DOTA			phe	Cys	1-Nal	trp	Lys	Thr	Cys	Thr-ol
In-DOTA-NOC	In-DOTA			phe	Cys	1-Nal	trp	Lys	Thr	Cys	Thr-ol
In-DOTA-BOC	In-DOTA			phe	Cys	BzThi	trp	Lys	Thr	Cys	Thr-ol
NOC-ATE				phe	Cys	1-Nal	trp	Lys	Thr	Cys	Thr
BOC-ATE				phe	Cys	BzThi	trp	Lys	Thr	Cys	Thr-ol
In-DOTA-NOC-ATE	In-DOTA			phe	Cys	1-Nal	trp	Lys	Thr	Cys	Thr
Lu-DOTA-NOC-ATE	Lu-DOTA			phe	Cys	1-Nal	trp	Lys	Thr	Cys	Thr
In-DOTA-BOC-ATE	In-DOTA			phe	Cys	BzThi	trp	Lys	Thr	Cys	Thr-ol
Lu-DOTA-BOC-ATE	Lu-DOTA			phe	Cys	BzThi	trp	Lys	Thr	Cys	Thr-ol
KE108	Y-DOTA	Tyr	dab	Arg	Phe	Phe	trp	Lys	Thr	Phe	
KE121			dab	Arg	Phe	Phe	trp	Lys	Thr	Phe	
Y-DOTA-K121 (Y-KE88)	Y-DOTA		dab	Arg	Phe	Phe	trp	Lys	Thr	Phe	
Ga-DOTA-K121 (Ga-KE88)	Y-DOTA		dab	Arg	Phe	Phe	trp	Lys	Thr	Phe	
Y-DOTAGA-KE121 (Y-KE87)	Y-DOTA		dab	Arg	Phe	Phe	trp	Lys	Thr	Phe	
Antagonists											
In-DOTA-BASS	In-DOTA			pNO_2_^−^Phe	cys	Tyr	trp	Lys	Thr	Cys	tyr-NH_2_
In-DOTA-JR11	In-DOTA			Cpa	cys	Aph(Hor)	Aph(Cbm)	Lys	Thr	Cys	tyr-NH_2_
Ga-DOTA-JR11 (Ga-OPS201)	Ga-DOTA			Cpa	cys	Aph(Hor)	Aph(Cbm)	Lys	Thr	Cys	tyr-NH_2_
Ga-NODAGA-JR11 (Ga-OPS202)	Ga-NODAGA			Cpa	cys	Aph(Hor)	Aph(Cbm)	Lys	Thr	Cys	tyr-NH_2_
Lu-DOTA-JR11 (Lu-OPS201)	Lu-DOTA			Cpa	cys	Aph(Hor)	Aph(Cbm)	Lys	Thr	Cys	tyr-NH_2_
sst_3_-ODN-8		NH_2_CO	cys	Phe	Tyr	DAgl8(Me,2-naphthoyl)	Lys	Thr	Phe	Cys	
DOTA-sst_3_-ODN-8	DOTA	NH_2_CO	cys	Phe	Tyr	DAgl8(Me,2-naphthoyl)	Lys	Thr	Phe	Cys	
DOTA-TOC	DOTA	d-Phe	Cys	Phe	d-Trp	Lys	Thr	Cys	Thr(ol)		

AgI, *α*-Aminoglycyl; Aph(Cbm), 4-aminocarbamoylphenylalanine; Aph(Hor), 4-amino-L-hydroorotylphenylalanine; BOC, [BzThi3]-octreotide; BOC-ATE, [BzThi3]-octreotate; BzThi, 3-benzothienylalanine; Cpa, 4-Cl-phenylalanine; Dab, *α*,*γ*-diaminobutyryl; DOTA, 1,4,7,10-tetraazacyclododecane-1,4,7,10-tetraacetic acid; DOTAGA, 1-(1-carboxy-3-carboxy-propyl)-4,7,10(carboxymethyl)-1,4,7,10-tetraazacyclo-dodecane; DTPA, diethylenetriaminepentaacetic acid; Gal-S, galactosyl-mercaptopropionyl; Gluc, glucosyl; Gluc-S, glucosyl-mercaptopropionyl; Gluc-Lys(FP), Nα-glucosyl-Nε-(2-fluoropropionyl)Lys; 1-Nal, 1-naphthylalanine; NOC, [1-Nal3]-octreotide; TATE, [Tyr3,Thr8]-octreotide; TOC, [Tyr3]-octreotide.

**Fig. 25.**
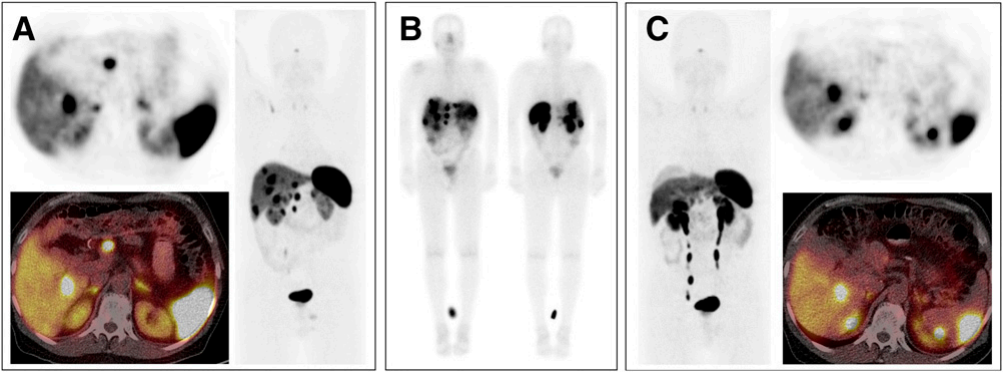
Representative examples of clinical PRRT images. PRRT in a 73-year-old patient with metastasized neuroendocrine cancer (G1). Pretherapeutic 68Ga-DOTANOC PET/computed tomography images show extensive metastases in the liver and additional abdominal lymph node metastases (A). Post-therapeutic whole-body scintigraphy after application of 177Lu-DOTATATE confirms uptake in metastatic lesions (B). After four cycles of 177Lu-DOTATATE, 68Ga-DOTANOC PET/computed tomography demonstrates considerable response of liver and abdominal lymph node metastases (C). Images courtesy of M. Eiber, Department of Nuclear Medicine, Technical University Munich, Germany.

After having established a noninvasive imaging methodology for NETs, the next logical step was the development of a treatment option, similar to the imaging and treatment of thyroid cancer with ^123^I- and ^131^I-iodide, respectively. Despite promising initial results after treatment of patients with very high doses of [^111^In]pentetreotide (Valkema et al., 2002) (up to 2.7 Ci in total) by means of the Auger and conversion electrons emitted by ^111^In, it became apparent that more efficient *β*-emitters, such as ^90^Y-yttrium (*t*_1/2_ = 64.1 hours, E*β*_max_= 2.28MeV), might be better suited for SST-targeted PRRT (Otte et al., 1997; Paganelli et al., 1999; Waldherr et al., 2001; Barone et al., 2005; Baum et al., 2012). These developments were based on the successful evaluation of 1,4,7,10-tetraazacyclododecane-1,4,7,10-tetraacetic acid (DOTA) as chelator for therapeutic radiometals with improved thermodynamic and kinetic stability, suitable for all commonly used M(III) radiometals, such as ^90^Y and ^177^Lu for PRRT, ^111^In for PRI with SPECT, and ^68^Ga for PRI with positron emisson tomography (PET), to mention only a few (Albert et al., 1998). Shortly after the introduction of ^90^Y-labeled SST ligands for peptide receptor therapy, it became apparent that methods to reduce the renal tracer uptake and thus to protect the kidneys were needed. Hammond et al. (1993) were the first who introduced the concept of coinfusion of Lys/Arg solutions to reduce the renal uptake of [^111^In]Octreoscan by >55%. Despite this nephron protection, some patients suffered from renal failure after ^90^Y-DOTA-d-Tyr^3^-octreotide (DOTATOC) treatment. To overcome these limitations, ^90^Y was substituted by ^177^Lu [*t*_1/2_ = 6.71 days, E*β*_max_= 497 keV, E*γ* = 113 keV (6.4%), and 208 keV (11%)], a *β*^−^ emitter with shorter penetration depth and coemission of low energy photons, thus allowing therapy monitoring by means of SPECT. Consequently, Lu-labeled SST ligands, e.g., [^177^Lu]DOTA-Tyr^3^-octreotate ([^177^Lu]DOTATATE), became the PRRT agent of choice (Fig. 25).

Concomitantly, and with the aim to increase the SST affinity, to modify the SST-binding profile, and to increase the tumor uptake of radiolabeled SST-binding peptides after administration of doses typically in the range of 15–30 nmol for imaging and 100–300 nmol for PRRT, various independent structural modifications of octreotide were introduced during the last 20 years, three of which should be mentioned in this work: 1) Tyr^3^, introduced into octreotide by Krenning et al. (1989) to be able to label the peptide with radioiodine, was found to significantly improve binding affinity and thus replaced d-Phe3 in a variety of subsequent octreotide analogs; 2) Thr^8^(ol), originally introduced to increase the in vivo stability of octreotide, was substituted by Thr^8^, leading to Tyr^3^-octreotate (TATE); and 3) substitution of Tyr^3^ by 1-naphthylalanin, resulting in DOTA-d-Nal^3^-octreotide (DOTANOC) ligands (i.e., [^68^Ga]DOTANOC) with somewhat increased lipophilicity but high affinity to SST_2_, SST_3_, and SST_5_ (see below). Until today, the above-mentioned three DOTA-conjugated peptides, DOTATOC, DOTATATE, and DOTANOC, are the most often clinically used SST ligands for imaging (e.g., ^68^Ga-labeled DOTATOC, DOTATATE, and DOTANOC) and therapy (e.g., ^177^Lu-labeled DOTATOC and DOTATATE) (Antunes et al., 2007; Schottelius et al., 2015).

The first clinical studies with [^177^Lu]DOTATATE started in 2000 in Rotterdam and formed the basis of a multinational neuroendocrine tumors therapy phase III trial named NETTER-1 (Strosberg et al., 2017) at 41 global sites. NETTER-1 demonstrated that [^177^Lu]DOTATATE significantly improved progression-free survival (compared with octreotide injection, Sandostatin LAR; 60 mg; Novartis, Basel, Switzerland) in patients with advanced midgut NETs.

Additional eight-amino-acid–containing peptide agonists such as lanreotide and vapreotide have been developed but never achieved relevance for PRI and PRRT (Breeman et al., 2001). In 2016, a “shake and bake” kit preparation of ^68^Ga-labeled (*t*_1/2_ = 68 minute) DOTATOC (Netspot; Advanced Accelerator Applications, Saint-Genis-Pouilly, France) was approved by the FDA for PET imaging of NETs, whereas the corresponding ^68^Ga-kit of the Thr^8^-analog [^68^Ga]DOTATATE was approved by the European Medicines Agency (SomaKIT TOC; Advanced Accelerator Applications). In addition, attempts were undertaken to optimize targeting properties of the first SST-imaging agent, [^123^]Tyr^3^-octreotide, and to transfer this tracer methodology to other radiohalogens, i.e., to the most commonly used PET radioisotope, fluorine-18 (*t*_1/2_ = 109.7 minutes). Unfortunately, until recently, methods for direct radiofluorination were not available, and complex multistep preparations, which are inherently hard to automate, were developed. To overcome the limitations accompanied by radioiodination and conjugation of peptides with typically used ^18^F-labeled prosthetic groups, i.e., a significant increase in lipophilicity and the resulting suboptimal imaging characteristics, Schottelius et al. (2004) adapted an interesting approach developed in the early 1990s by Albert et al. (1993) to improve oral availability of SST-binding peptides by glycosylation. Compensation of the negative influence of radiohalogenation by the use of carbohydrated peptides was highly effective, both for radioiodinated and ^18^F-SST ligands (Wester et al., 2003, 2004; Schottelius et al., 2004), resulting in radiopharmaceuticals with excellent imaging properties. In a pilot study in 25 patients, Nα-(1-deoxy-D-fructosyl)-Nε-(2-[^18^F]fluoropropionyl)-Lys^0^-Tyr^3^-octreotate PET (Gluc-Lys[^18^F]FP-TATE) allowed fast, high-contrast imaging of SST-positive tumors. The biokinetics and diagnostic performance of Gluc-Lys([^18^F]FP)-TATE was superior to [^111^In]DTPA-octreotide and comparable with [^68^Ga]DOTATOC (Meisetschläger et al., 2006).

To improve imaging quality and the availability of a suitable SPECT imaging agent, ^99m^Tc-labeled analogs of octreotide (*t*_1/2_ = 6 hours), such as ^99m^Tc-tricine-HYNIC-Tyr^3^-octreotide (Bangard et al., 2000), have been developed and successfully established for routine clinical use (Trejtnar et al., 2000; Gabriel et al., 2003; Hicks, 2010). Furthermore, [^99m^Tc]ethylenediamine-N,N′-diacetic acid/HYNIC-Tyr^3^-octreotide (Tektrotyd, NCBJ RC POLATOM) has been approved in a number of European and non-European countries.

#### 2. Pan Somatotropin-Release Inhibitory Factor–Like Peptides

Radiolabeled ligands that bind with similar high affinity to all five SSTs, so-called panSRIF-like ligands, are expected to expand the clinical indications of currently applied predominantly SST_2_-targeted ligands and to significantly improve tumor targeting, imaging sensitivity, and therapeutic efficacy by crossreactivity to coexpressed SST_1_, SST_3_, SST_4_, and SST_5_. One of the first developments in that direction was [^111^In]DOTANOC (Wild et al., 2003), which showed high affinity to SST_2_, SST_3_, and SST_5_, and finally resulted in [^68^Ga]DOTANOC (Wild et al., 2005), one of the most frequently used PET-imaging agents (already mentioned above), whereas the use of other compounds, such as DOTA-1-naphthylalanin,Thr^8^-octreotide (DOTA-NOC-ATE) or DOTA-BzThi^3^,Thr^8^-octreotide (DOTA-BOC-ATE) with even higher and broader affinity remained limited in use (Ginj et al., 2005, 2006a). Fani et al. (2010) developed bicyclic analogs, such as DOTA-Tyr-cyclo(DAB-Arg-cyclo(Cys-Phe-D-Trp-Lys-Thr-Cys)) (AM3; affinity for SST_2_, SST_3_, and SST_5_). The authors concluded that due to its rapid background clearance and high tumor to nontumor ratios, ^68^Ga-AM3 might be an ideal PET-imaging agent (Fani et al., 2010). The first such peptide with high-affinity binding for all five receptor subtypes was KE108 [Y-DOTA-Tyr-cyclo(D-Dab-Arg-Phe-Phe-D-Trp-Lys-Thr-Phe)] (Reubi et al., 2002) and its DOTA analog [^111^In]KE88 (Ginj et al., 2008). Unfortunately, this peptide was only efficiently internalized in SST_3_-expressing cells and did not offer pan-receptor–imaging properties. Another cyclic peptide, DOTA-pasireotide with affinity for four SST subtypes (SST_1_, SST_2_, SST_3_, and SST_5_), has also been evaluated, with limited success. For both pasireotide- and KE108-based radioligands, the absence of SST_2_ internalization may turn out to be a serious disadvantage and compromise their accumulation in target cells, because in most cases SST_2_ overexpression prevails.

Recently, native SRIF-14 and its d-Trp^8^ analog were considered for ligand development. Not unexpectedly, [^111^In]DOTA-SS14 and [^111^In]DOTA-DTrp^8^-SS14 showed high affinity to all human SST subtypes, and [^111^In]DOTA-DTrp^8^-SS14 localized in experimental tumors, which selectively expressed rat SST_2_, human SST_2_, SST_3_, and SST_5_ (Tatsi et al., 2012). Furthermore, Maina et al. (2014) evaluated the SRIF mimic [^111^In]DOTA-LTT-SS28 [(DOTA)Ser^1^,Leu^8^,D-Trp^22^,Tyr^25^-SS28]. DOTA-LTT-SS28 exhibited a panSRIF-like binding profile (IC_50_ values for all SST subtypes in the low nanomolar range); behaved as an agonist at human SST_2_, SST_3_, and SST_5_; and efficiently stimulated internalization of the three receptor subtypes. In addition, significant and specific uptake was observed in HEK293-SST_2_–, HEK293-SST_3_–, and HEK293-SST_5_–expressing tumors. The authors concluded that [^111^In-DOTA]LTT-SS28 might be a promising ligand for multi-SST_1_–SST_5_–targeted tumor imaging.

Taking into account that high in vivo stability of a peptide radiopharmaceutical is of utmost importance for successful tumor imaging and PRRT, the re-evaluation of native or slightly modified SRIF-14 and SRIF-28 needs to be considered in the context of peptidase activity in vivo. Neutral endopeptidase is responsible for rapid breakdown of i.v. administered SRIF-, bombesin-, and gastrin-derived peptides, and activity of neutral endopeptidase can be overcome through the mere coinjection of a protease inhibitor, such as phosphoramidone (Nock et al., 2014). This approach may result in enhanced supply and accumulation of these radiopeptides at tumor sites and in increased clinical diagnostic sensitivity and therapeutic efficacy (Kaloudi et al., 2015, 2016).

#### 3. Antagonists

Radiolabeled antagonists for imaging of cerebral receptor systems were established early in the application of noninvasive SPECT and PET imaging (Wagner et al., 1983). Internalization of the receptor after radioligand binding has been assumed to be critical for efficient retention of peptide radiopharmaceuticals in tumor cells and a prerequisite for efficient PRI and PRRT imaging. Almost all SST-binding peptide lead structures exploited for radiopharmaceutical development originate from development of SST-targeting drugs (e.g., octreotide) and exhibited agonistic behavior. The first results indicating that high-affinity SST antagonists that poorly internalize into tumor cells perform more effectively than corresponding agonists that are highly internalized into tumor cells were considered to be at the forefront of a paradigm shift in nuclear oncology imaging (Ginj et al., 2006b).

Motivated by a study of Bass et al. (1996), who found that inversion of the chirality at positions 1 and 2 of the octreotide peptide family converts an agonist to an antagonist, and by structure activity relationship studies of Hocart et al. (1998, 1999), the first radiolabeled SST antagonists were evaluated (Cescato et al., 2008). Ginj et al. (2006b) showed that two peptides with high affinity to SST_2_ ([^111^In]DOTA-sst_2_-ANT) and SST_3_ ([^111^In]DOTA-sst_3_-ODN-8), respectively, did not trigger SST_2_- or SST_3_-mediated internalization and prevented agonist-stimulated internalization. Subsequent biodistribution studies in mice bearing SST_3_-expressing tumors revealed strong accumulation of [^111^In]DOTA-SST_3_-ODN-8 at 1 hour with up to 60% of injected radioactivity per gram of tissue and maintained at a high level for >72 hours, whereas [^111^In]DOTA-NOC, with strong SST_3_-binding and internalization properties, showed a much lower and shorter-lasting uptake in SST_3_-expressing tumors. The same tendency was seen for SST_2_-binding ligand [^111^In]DOTA-SST_2_-ANT when compared with the highly potent SST_2_-selective agonist [^111^In]DTPA-TATE, suggesting that this observation may be valid for more than just one particular GPCR.

A pilot study in five patients with NETs or thyroid cancer provided the first evidence that SST imaging with [^111^In]DOTA-sst_2_-ANT ([^111^In]DOTA-BASS) is significantly more sensitive and effective than that employing the FDA-approved radiotracer ^111^In-DTPA-octreotide (OctreoScan; Mallinckrodt) (Wild et al., 2011). In a comprehensive preclinical study, three different SST_2_ antagonists, LM3 (*p*-Cl-Phe-cyclo(D-Cys-Tyr-D-Aph(Cbm)-Lys-Thr-Cys)D-Tyr-NH_2_), JR10 (*p*-NO_2_-Phe-c[D-Cys-Tyr-D-Aph(Cbm)-Lys-Thr-Cys]-D-Tyr-NH_2_), and JR11 (Cpa-c[D-Cys-Aph(Hor)-D-Aph(Cbm)-Lys-Thr-Cys]-D-Tyr-NH_2_), were evaluated in combination with two chelators [DOTA and 1,4,7-triazacyclononane,1-glutaric acid-4,7-acetic acid (NODAGA)] and various (radio)metals [In(III), Y(III), Lu(III), Cu(II), and Ga(III)]. Although the antagonists were found to be very sensitive to chelator modifications and complexation with distinct radiometals (Fani et al., 2012), the study illustrated the potential of the antagonists, because even a low-affinity antagonist was shown to be slightly superior to a high-affinity agonist, outweighing the affinity differences. This is due to the fact that a neutral antagonist labels receptors in all states (active or inactive), whereas an agonist only labels receptors in an active conformation. The active conformation may represent a limited proportion of the whole population, because most GPCRs show low levels of constitutive activity.

JR11 was selected for clinical development as a PET-imaging agent labeled with ^68^Ga using the chelator NODAGA (^68^Ga-NODAGA-JR11 or ^68^Ga-OPS202) and as a therapeutic agent labeled with ^177^Lu using the chelator DOTA (^177^Lu-DOTA-JR11 or ^177^Lu-OPS201). In a preclinical comparison of the antagonist [^177^Lu]OPS201 (DOTA-JR11; DOTA-[Cpa-c(DCys-Aph(Hor)-DAph(Cbm)-Lys-Thr-Cys)-DTyr-NH_2_]) and the SST_2_ agonist [^177^Lu]DOTATATE, the antagonist showed 2.5-times higher tumor dose, longer tumor residence time, and 1.3-fold higher tumor-to-kidney dose ratio (Nicolas et al., 2017). A phase I/II PET/computed tomography study for interindividual comparison of ^68^Ga-NODAGA-JR11 (^68^Ga-OPS202) and ^68^Ga-DOTATOC indicated increased image contrast, sensitivity, and diagnostic accuracy of ^68^Ga-OPS202 for staging of gastroenteropancreatic NETs (Nicolas et al., 2018). The theranostic pair ^68^Ga-DOTA-JR11 and ^177^Lu-DOTA-JR11 has also been investigated in NET patients, and ^177^Lu-DOTA-JR11 (^177^Lu-OPS201) is being evaluated in phase I/II.

More than 20 years after approval of [^111^In]Octreoscan, recent regulatory approvals of [^68^Ga]DOTATATE and [^68^Ga]DOTATOC and the expected authorization of [^177^Lu]DOTATATE will significantly advance the field and stimulate further peptide receptor-based imaging and therapy options. With respect to tracer development, recent studies with radiolabeled antagonists have generated high expectations that require verification in detailed clinical studies. Combination of [^177^Lu]DOTATATE radiotherapy with chemotherapy, targeted agents, or immunotherapies has been initiated. Data of a first phase III study comparing the combination of ^177^Lu PRRT and capecitabine (Xeloda; Roche, Basel, Switzerland), an oral chemotherapy agent, with ^177^Lu PRRT alone started at Erasmus MC (Rotterdam, Netherlands) are expected in 2017 (van Essen et al., 2008). Further studies on combination therapies, named peptide receptor chemoradionuclide therapy, have recently been published (Kong et al., 2017).

## XI. Conclusions

The SRIF system comprises seven genes encoding two peptide precursors, SRIF and CST, and five receptors. Compared with many other regulatory peptides, this is a relatively high number of receptors. It remains an intriguing question why this system needs five different receptors to transduce the SRIF signal. To date, far few disease-associated mutations have been identified. KO mice for any of the SSTs exhibit rather mild phenotypes. This suggests a high functional redundancy with potential that loss of one SST can be compensated by another SST subtype. Although SSTs often show overlapping distributions, they exhibit striking differences in their subcellular localizations and trafficking. SST_2_ and SST_5_ receptors are primary targets for pharmacological treatment of pituitary adenomas and NETs. In addition, SST_2_ is a prototypical GPCR for development of peptide-based radiopharmaceuticals for diagnostic and therapeutic intervention. Consequently, the localization, regulation, and function of the five SSTs have been studied extensively in vitro and in vivo. However, open questions remain:

- Are there additional receptors for SRIF and/or CST?
- Does heterodimerization among SSTs and other GPCRs occur in vivo?
- Can SSTs signal from within intracellular compartments?
- What is the exact mechanism involved in the SST and AIP–Zac1 pathway?
- Why do sparsely granulated GH-secreting adenomas not respond well to SRIF analogs?
- Why do cell lines not respond well to SRIF analogs?
- What is the molecular mechanism underlying tumor imaging using SST antagonists?
- What is the therapeutic potential for development of biased SST agonists?
- Is there a potential for SST ligands that can penetrate the blood brain barrier and enter the CNS?
- How are targeting and membrane trafficking of SST_1_ regulated?
- What is the precise physiologic role of truncated SST_5_ variants?

The future challenges include deciphering crystal structures for the five SSTs to facilitate discovery of novel SST subtype-selective agonists and antagonists, which are both safe and effective. It is expected that novel delivery ligand systems including oral formulations and longer-acting injectables will offer enhanced patient convenience for long-term therapies. In the future, SRIF-based therapies may become available for novel indications, such as treatment of type 2 diabetes with SST_5_ antagonists or treatment of neuropathic pain with SST_4_ agonists.

## Abbreviations

ACTH: adrenocorticotropic hormone
AIP: aryl hydrocarbon receptor-interacting protein
AP: activator protein
ARC: arcuate nucleus
BBS: Bardet–Biedl syndrome
CA: Cornu Ammonis
CCK: cholecystokinin
CD26: dipeptidyl peptidase-4/cluster of differentiation 26
CHO: Chinese hamster ovary
CNS: central nervous system
CRF: corticotropin-releasing factor
CRH: corticotropin-releasing hormone
CST: cortistatin
D_2_ receptor: dopamine receptor D2
DOTA: 1,4,7,10-tetraazacyclododecane-1,4,7,10-tetraacetic acid
DOTANOC: DOTA-d-Nal^3^-octreotide
DOTATATE: DOTA-Tyr^3^-octreotate
DOTATOC: DOTA-d-Tyr^3^-octreotide
DRG: dorsal root ganglion
DTPA: diethylenetriaminepentaacetic acid
ECE-1: endothelin-converting enzyme 1
ECL: extracellular loop
ERK: extracellular signal-regulated kinase
FDA: Food and Drug Administration
FGF: fibroblast growth factor
FLNA: filamin A
GEP: gastroenteropancreatic
GH: growth hormone
GHRH: GH-releasing hormone
GHS-R1a: ghrelin receptor 1a
GI: gastrointestinal
GIST: GI stromal tumor
GIT: GI tract
GPCR: G protein–coupled receptor
GRK: G protein–coupled receptor kinase
HCC: hepatocellular carcinoma
HEK: human embryonic kidney
ICL: intracellular loop
IGF-1: insulin-like growth factor 1
IL: interleukin
ITIM: immunoreceptor tyrosine-based inhibition motif
IUPHAR: International Union of Basic and Clinical Pharmacology
JAK2: Janus kinase 2
KO: knockout
LAR: long-acting release
LNPEP: leucyl-cysteinyl aminopeptidase
mAb: monoclonal antibody
MAPK: mitogen-activated protein kinase
MIBP1: c-myc intron binding protein 1
mTOR: mammalian target of rapamycin
MUPP1: multi PDZ-domain protein 1
NET: neuroendocrine tumor
NHE1: sodium/hydrogen exchanger 1
NHERF: sodium/hydrogen exchanger regulatory factor 1
NODAGA: 1,4,7-triazacyclononane,1-glutaric acid-4,7-acetic acid
NOS: nitric oxide synthase
PDZ: PSD-95/discs large/ZO-1
PET: positron emission tomography
PI3K: phosphatidylinositol-4,5-bisphosphate 3-kinase
PIST: protein interacting specifically with Tc10
PKA: protein kinase A
PLC: phosphoinositide-specific phospholipase C
PNGase F: peptide N-glycosidase F
PP1: protein phosphatase 1
PRI: peptide receptor imaging
PRRT: peptide-receptor radiotherapy
PSD: postsynaptic density
PTP: protein tyrosine phosphatase
PTP*_η_*: protein tyrosine phosphatase *η*
PTX: pertussis toxin
Rab: Ras-related in brain
Ras: rat sarcoma
Rho: Ras homolog
SEF-2: helix-loop-helix transcription factor
SHP: Src homology region 2 domain-containing phosphatase
SPECT: single-photon emission computed tomography
SRIF: somatotropin-release inhibitory factor
SST: somatostatin receptor
SST_5_TMD: SST_5_ transmembrane domain
*t*_1/2_: half-life
T3: triiodthyronine
TATE: Tyr^3^-octreotate
TGN: trans-Golgi network
TMD: transmembrane domain
TSH: thyroid-stimulating hormone
TSHoma: TSH-secreting pituitary adenoma
UFC: urinary-free cortisol
UTR: untranslated region
VOCC: voltage-operated calcium channel
Zac1: zinc finger protein 1
